# Accelerated Zeolite Defect Healing Suppresses Oxygen-Containing Species Trapping for Tandem Propene Epoxidation

**DOI:** 10.1021/jacs.6c17355

**Published:** 2026-10-16

**Authors:** Dong Lin, Xin Zhou, Yang Xu, Richard J. Lewis, Zongzhuang Sun, Defu Yin, Yongxiang Wang, Zhenyi Liu, Jingyuan Shang, Zhe Ma, Qiang Wang, Feng Deng, Chaohe Yang, De Chen, Graham J. Hutchings, Xiang Feng

**Affiliations:** † State Key Laboratory of Heavy Oil Processing, China University of Petroleum, Qingdao 266580, China; ‡ Max Planck-Cardiff Centre on the Fundamentals of Heterogeneous Catalysis FUNCAT, Cardiff Catalysis Institute, School of Chemistry, 2112 Cardiff University, Cardiff CF24 4HQ, U.K.; § College of Chemistry and Chemical Engineering, Ocean University of China, Qingdao 266100, China; ∥ National Centre for Magnetic Resonance in Wuhan, State Key Laboratory of Magnetic Resonance and Atomic and Molecular Physics, Wuhan Institute of Physics and Mathematics, Innovation Academy for Precision Measurement Science and Technology, 12381 Chinese Academy of Sciences, Wuhan 430071, China; ⊥ Department of Chemical Engineering, Norwegian University of Science and Technology, Trondheim 7491, Norway

## Abstract

Selective oxidation of chemical feedstocks with in situ-generated H_2_O_2_ offers a sustainable route to high-value oxygenates, yet such tandem catalysis is often fundamentally limited by the trapping of oxygen-containing species, including H_2_O_2_-derived intermediates and oxygenated products in zeolite microenvironments, where they undergo nonproductive decomposition and side reactions. Herein, we overcome this limitation by accelerating titanosilicalite hydroxyl defect healing through intensified nucleation and subsequent accelerated crystal growth. The resulting defect-healed microenvironment facilitates the transport of in situ-generated H_2_O_2_ between bifunctional active sites while suppressing its nonproductive decomposition to H_2_O. Moreover, it accelerates product propylene oxide (PO) desorption by weakening hydrogen bond interactions between PO and hydroxyl defects, thereby suppressing ring-opening and overoxidation side reactions. Consequently, the defect-healed Au/TS-1 catalyst exhibits a 1.8-fold enhancement in the PO formation rate and a 2.1-fold increase in Au-based productivity. This defect-healing strategy is broadly applicable to titanosilicates, aluminosilicates, and pure-silica zeolites and can be readily extended to a 10 L scale process, delivering reductions of 21% in production cost and 54% in carbon emissions compared with conventional TS-1 synthesis. Overall, this work establishes a sustainable, scalable, and cost-effective route for zeolite defect healing and provides a general strategy to overcome the long-standing transport-decomposition trade-off in tandem catalysis with in situ-generated H_2_O_2_.

## Graphical abstract

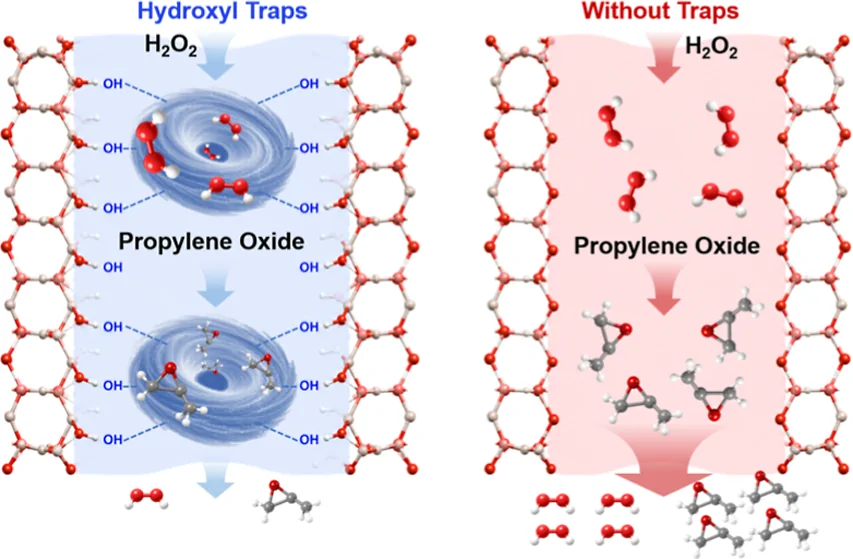

## Introduction

Propene oxide (PO) is a key bulk chemical intermediate widely used in the production of polyether polyols, propylene glycol, dimethyl carbonate, and numerous value-added chemicals for the polyurethane and fine chemical industries.^1–4^ Driven by the rapid growth of these sectors, global PO demand reached approximately 14 million tons in 2025 and is projected to increase at a compound annual rate of 5–6% over the next decade, highlighting the need for more sustainable and efficient production technologies.^5^ Conventional industrial processes, such as the chlorohydrin and hydroperoxide routes, are associated with some drawbacks, including waste generation, coproduct dependence, and complex separation steps.^6–8^ In this context, the direct epoxidation of propene using H_2_ and O_2_ has emerged as a promising alternative, as it enables the in situ generation of H_2_O_2_ as a clean oxidant, offering simplified process integration, improved atom economy, and clear economic and environmental advantages.^9–12^

Since the pioneering work of Haruta and co-workers, Au/TS-1 has become the benchmark catalyst for tandem propene epoxidation with H_2_ and O_2_ because Au nanoparticles, unlike conventional Pd and Pt catalysts, selectively generate H_2_O_2_ in situ while effectively suppressing propene hydrogenation.^13–15^ In this tandem system, catalytic performance is determined not only by the in situ H_2_O_2_ generation over active metal sites and epoxidation over framework Ti sites but also by the often-overlooked preservation and transport of oxygen-containing species, including the intermediate H_2_O_2_ and the product propylene oxide (PO), in the zeolite microenvironment.^16–18^ Efficient coupling requires H_2_O_2_ or its associated active species (e.g., OOH) generated on Au to reach Ti sites and form Ti–OOH intermediates before decomposition, while the PO product must desorb and diffuse out of the channels before secondary reactions occur. Local interactions between oxygen-containing species and the zeolite microenvironment can induce molecular trapping effects, prolonging the residence time of H_2_O_2_-derived intermediates and PO products.^19–21^ These trapping effects can further perturb their chemical stability and redirect them toward nonselective transformation pathways.^22–24^ Therefore, weakening such microenvironment–molecule interactions provides a promising strategy to suppress molecular trapping effects and improve overall tandem epoxidation efficiency.

In this work, molecular trapping was suppressed by accelerating the defect healing in the titanosilicalite microenvironment. Healing hydroxyl-associated defects via surface reconstruction driven by intensified nucleation and subsequent accelerated crystal growth markedly weakens hydrogen-bonding interactions with oxygen-containing species, namely, the H_2_O_2_ intermediate and the PO product. This weakened microenvironment–molecule interaction reduces the retention and destabilization of oxygen-containing species, thereby preventing H_2_O_2_ from being trapped and decomposed to H_2_O near Au sites while also facilitating PO release from hydroxyl traps before ring-opening and overoxidation around Ti sites. As a result, molecular trapping is effectively suppressed, thereby synergistically improving H_2_O_2_ transport and the coupled epoxidation reaction, leading to an enhanced tandem propene epoxidation. The resulting defect-healed titanosilicalite loaded with Au (Au/L-TS-1) exhibits a 1.8-fold enhancement in the PO formation rate compared to the conventional Au-loaded unhealed titanosilicalite catalyst, reaching approximately 330 g_PO_ h^–1^ kg_cat_^–1^, together with a 2.1-fold increase in Au-specific productivity to about 485 g_PO_ h^–1^ g_Au_^–1^, while maintaining PO selectivity above 80%. This defect-healing strategy is broadly applicable to titanosilicates, aluminosilicates, and pure-silica zeolites and is readily scalable to a 10 L process, reducing production costs by 21% and carbon emissions by 54% relative to conventional TS-1 synthesis.

## Results and Discussion

### Suppressing Trapping of Oxygen-Containing Species by Accelerated Zeolite Defect Healing

In tandem propene epoxidation, catalytic performance depends not only on H_2_O_2_ generation over metal sites and further epoxidation over framework Ti species, but also on the often-overlooked preservation and transport of oxygen-containing species (H_2_O_2_ and PO) in the zeolite microenvironment. Interactions between these species and the trap-rich microenvironment prolong their residence time and promote nonselective transformation pathways. Suppressing such molecular trapping effects in the zeolite microenvironment offers an effective strategy to enhance tandem epoxidation, as shown in Figure 1a.

**Figure 1:**
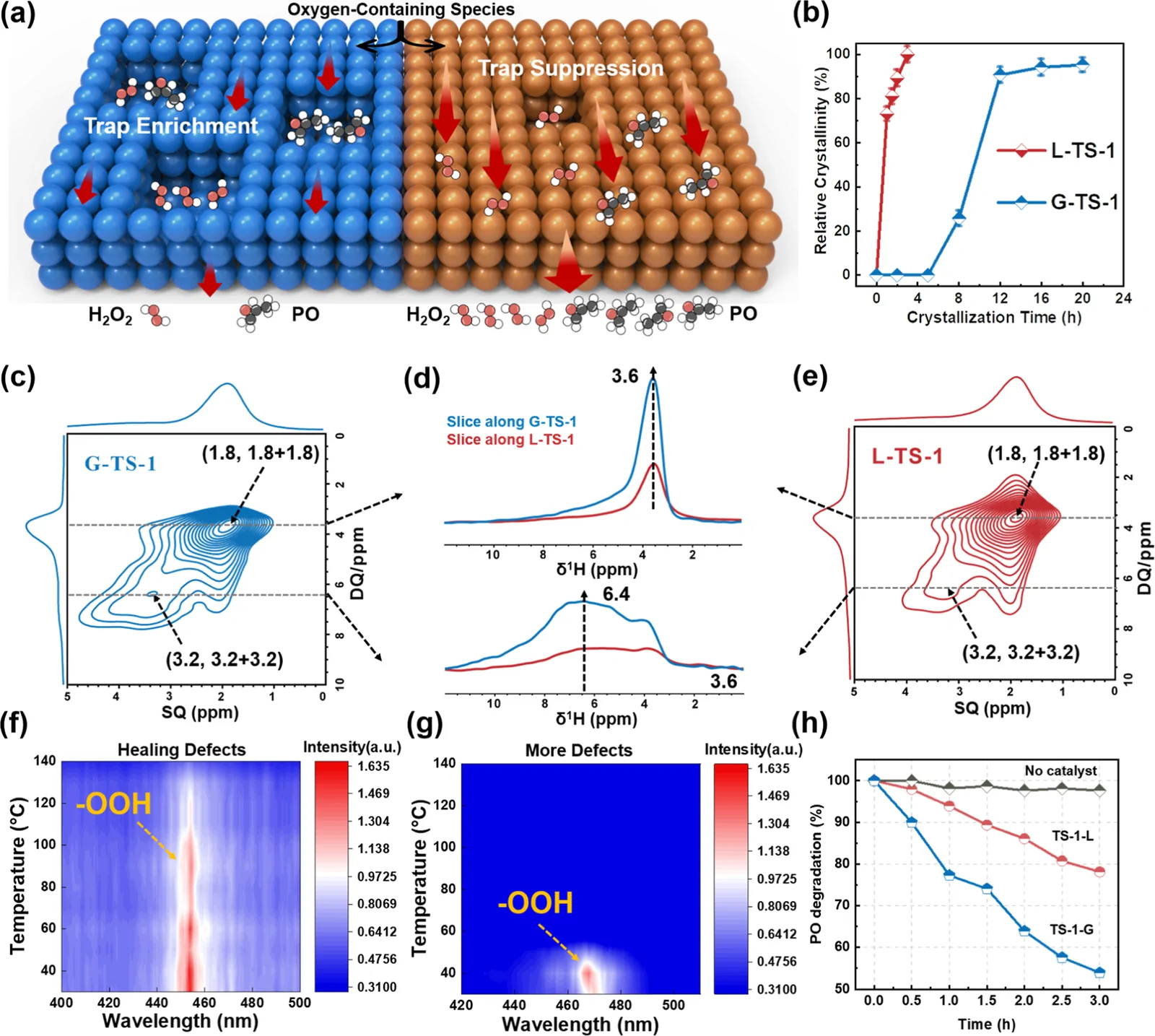
(a) Schematic illustration of trapping effects on oxygen-containing species for H_2_O_2_ and PO in propene epoxidation with H_2_ and O_2_. (b) Relative crystallinity of titanosilicalites synthesized in different heat transfer media at different crystallization times. The 2D ^1^H–^1^H double quantum-single quantum (DQ-SQ) MAS NMR spectra of (c) G-TS-1 and (e) L-TS-1 titanosilicalites. (d) Slice of 2D ^1^H–^1^H DQ-SQ MAS NMR spectra of L-TS-1 and G-TS-1 titanosilicalites. In situ UV–vis spectra of (f) L-TS-1 and (g) G-TS-1 upon exposure to preformed H_2_O_2_. Experimental conditions: 0.2 g of TS-1 with 1.2 g of H_2_O_2_, measured from 30 to 140 °C. (h) Time-dependent PO degradation over L-TS-1 and G-TS-1 (60 °C, 1.2 g of PO, 1.2 g of 30 wt % H_2_O_2_, 20 mL of acetonitrile, and 0.2 g of catalyst).

In zeolites, such trap-rich microenvironments are closely associated with structural defects, which primarily originate from Brønsted acid sites and silanol groups.^25,26^ The Brønsted acid sites arise from the substitution of Si atoms in the zeolite framework, forming specific T–OH species (T = Ti, Al, etc.), whereas silanol groups (Si–OH) are typically associated with the framework structure.^27,28^ In this study, the healing of framework defects within the TS-1 porous microenvironment was regulated by controlling the thermal environment during crystallization using different heating media (dimethyl silicone oil and air). X-ray diffraction (XRD) analysis (Figures 1b and S1 and S2) revealed that TS-1 crystallized under a liquid-phase thermal environment (L-TS-1, dimethyl silicone oil, 170 °C, autogenous pressure) and exhibited a significantly accelerated crystallization process compared with that synthesized under gas-phase conditions (G-TS-1, air, 170 °C, autogenous pressure). As zeolite crystallization generally involves an initial induction (nucleation) stage followed by rapid crystal growth,^29,30^ the L-TS-1 sample displayed both a higher nucleation rate and an enhanced crystal growth rate than G-TS-1.

To compare the defects of TS-1 synthesized using different thermal environments, L-TS-1 obtained after 3 h and G-TS-1 obtained after 72 h, both exhibiting comparable crystallinity, were selected for investigation. The titanosilicalite defects typically exist in the form of silanols (Si–OH) and Brønsted acid sites (Ti–OH).^25^ ^29^Si MAS NMR (^29^Si magic angle spinning nuclear magnetic resonance) was used to determine the Si–OH species present in the titanosilicalites.^31^ As shown in Figure S3, the peaks at −104 and −113 ppm were attributed to the Si­(OSi)_3_OH (Q_3_) and Si­(OSi)_4_ (Q_4_) species, respectively.^32^ The Q_3_/Q_4_ ratio of L-TS-1 (5.3%) is considerably lower than that of G-TS-1 (10.9%), indicating that the L-TS-1 analogue had fewer Si­(OSi)_3_OH defects. Fourier transform infrared spectroscopy, using pyridine as a probe molecule (Py-IR), was further utilized to estimate the proportion of Brønsted and Lewis acid sites in the titanosilicalite (Figures S4 and S5).^33^ Both L-TS-1 and G-TS-1 showed intense peaks of Lewis acid sites at 100 °C, while only the G-TS-1 showed a weak Brønsted acid peak at 1550 cm^–1^, which could be attributed to small amounts of Ti–OH defect species. ^1^H MAS NMR was used to establish the presence of acidic defect sites, with those present in the L-TS-1 sample at 1.8 and 2.0 ppm assigned to the terminal hydroxyls.^34^ Notably, the concentration of these species in the L-TS-1 sample was considerably lower than that in the G-TS-1 analogue (Figure S6). Moreover, the resonance at 3.2 ppm, attributed to hydrogen-bonded silanol groups associated with framework defects, was significantly less intense in L-TS-1 than in G-TS-1. To provide spatial insights into various defect sites, two-dimensional (2D) ^1^H–^1^H MAS NMR was employed, as it offers superior resolution of proton resonances compared to one-dimensional (1D) NMR. In the 2D DQ-SQ ^1^H–^1^H MAS NMR spectra (Figure 1c–e), L-TS-1 exhibited weaker self-correlation signals at (1.8, 3.6) and (3.2, 6.4) ppm relative to G-TS-1, indicating a lower density of spatially proximate defect sites.^35^

Given the positive correlation between defect site concentration and acidity,^36^ ammonia temperature-programmed desorption (NH_3_-TPD) analysis was further used to characterize the concentration of acid sites in titanosilicalites. Figure S7 shows two peaks at 125 and 270 °C in the NH_3_-TPD spectra of both samples, which may be attributed to weak acid sites.^37^ Quantitative analysis revealed that L-TS-1 possesses 10% fewer acid sites than its G-TS-1 counterpart, which can be attributed to a lower density of defect sites. This finding is consistent with previous results obtained from ^1^H MAS NMR, ^29^Si MAS NMR, and Py-IR analyses. Additionally, high-resolution transmission electron microscopy (HRTEM) imaging further showed that the L-TS-1 had a smoother surface with fewer defects than that of G-TS-1 (Figure S8). These results demonstrate that accelerated defect healing creates a low-defect microenvironment in L-TS-1 relative to that in G-TS-1.

Further analyses were conducted to elucidate the structural characteristics of these two titanosilicalites. UV–vis analysis confirmed that both G-TS-1 and L-TS-1 feature tetrahedrally coordinated Ti species (∼210 nm) with no detectable extra-framework anatase (∼330 nm) in Figure S9. This is attributed to ultraviolet (UV) irradiation during the sol–gel preparation, which suppresses the formation of extra-framework Ti species by promoting the deoligomerization of Ti oligomers into monomeric species via hydroxyl radicals^38^ (Figures S10 and S11). HAADF-STEM and EDS mapping (Figures S12 and S13) revealed uniform elemental distributions (Ti, O, and Si), and nitrogen adsorption–desorption analysis showed comparable surface areas (∼450 m^2^/g) and pore structures (Table S1). Both G-TS-1 and L-TS-1 predominantly expose the (020) facet, with crystal growth preferentially occurring along the [100] direction (Figure S14). The unit cells of L-TS-1 (a = 20.10 Å, b = 19.91 Å, c = 13.40 Å) and G-TS-1 (a = 20.09 Å, b = 19.90 Å, c = 13.39 Å) exhibit similar orthorhombic structures (a ≠ b ≠ c), as shown in Figure S15. Overall, these analyses demonstrate that both defect-healed and non-healed TS-1 possess identical Ti active sites and similar crystal structures, while exhibiting distinct defect densities in the porous microenvironment.

The microenvironment with distinct defect densities further determines whether oxygen-containing species (key H_2_O_2_ intermediates and the PO product) can be transported or trapped and further consumed in side reactions. To investigate the trapping of H_2_O_2_-derived intermediates, in situ UV–vis spectroscopy was performed (Figure 1f,g). During the reaction, H_2_O_2_ interacts with framework Ti sites to form the key intermediate Ti–OOH. Trapping of H_2_O_2_ at defect sites restricts its diffusion to Ti sites, thereby suppressing downstream Ti–OOH formation. Consistently, the characteristic Ti–OOH absorption band (300–500 nm)^39^ is markedly stronger on L-TS-1 than on G-TS-1 below an analysis temperature of 50 °C. Above 50 °C, the Ti–OOH signal on G-TS-1 nearly disappears, whereas that on L-TS-1 remains pronounced up to 120 °C, indicating that defect healing suppresses H_2_O_2_ trapping and sustains Ti–OOH formation over a broader temperature range. PO trapping was further evaluated by PO degradation experiments (Figure 1h). Prolonged residence of PO at defect sites promotes ring-opening and overoxidation, whereas suppressing molecular trapping facilitates PO release from the catalyst and mitigates degradation. Negligible PO degradation occurs in the absence of the catalyst, while G-TS-1 (∼44%) exhibits approximately twice the degradation of L-TS-1 (∼22%). Together, these results indicate that defect healing suppresses the trapping of oxygen-containing species, preserving Ti–OOH intermediates and facilitating PO release.

### Effect of Defect Healing on Propene Epoxidation with H_2_ and O_2_

These TS-1 samples, which differ in defect states (healed vs non-healed) while maintaining comparable Si/Ti ratios (∼200), were loaded with Au nanoparticles (Table S2) and evaluated in the direct propene epoxidation with H_2_ and O_2_.

The propylene oxide (PO) formation rate was normalized per gram of Au and per gram of catalyst to quantify the Au utilization efficiency and overall catalytic performance, respectively. The defect-healed Au/L-TS-1 catalyst exhibited a 1.8-fold higher initial PO formation rate (340 vs 185 g_PO_ h^–1^ kg_Cat_^–1^) together with a 2.1-fold enhancement in Au efficiency (485 vs 231 g_PO_ h^–1^ g_Au_^–1^) compared with Au/G-TS-1 (Figure 2a). This defect-healed Au/L-TS-1 simultaneously delivers high PO formation rates on both Au and catalyst mass bases, outperforming previously reported systems without promoters (Figure 2b and Table S3). The defect-healed Au/L-TS-1 catalyst exhibits a 10% increase in epoxide selectivity (Figure 2c,d) and a 13% relative improvement in initial hydrogen efficiency (Figure S16) relative to the Au/G-TS-1 analogue, indicating suppressed side reactions from PO and more efficient H_2_O_2_ utilization. As defect sites are progressively healed, hydroxyl-mediated molecular trapping in the zeolite microenvironment is suppressed, thereby restraining unproductive pathways and limiting the formation of byproducts, such as propanal and acetone.

**Figure 2:**
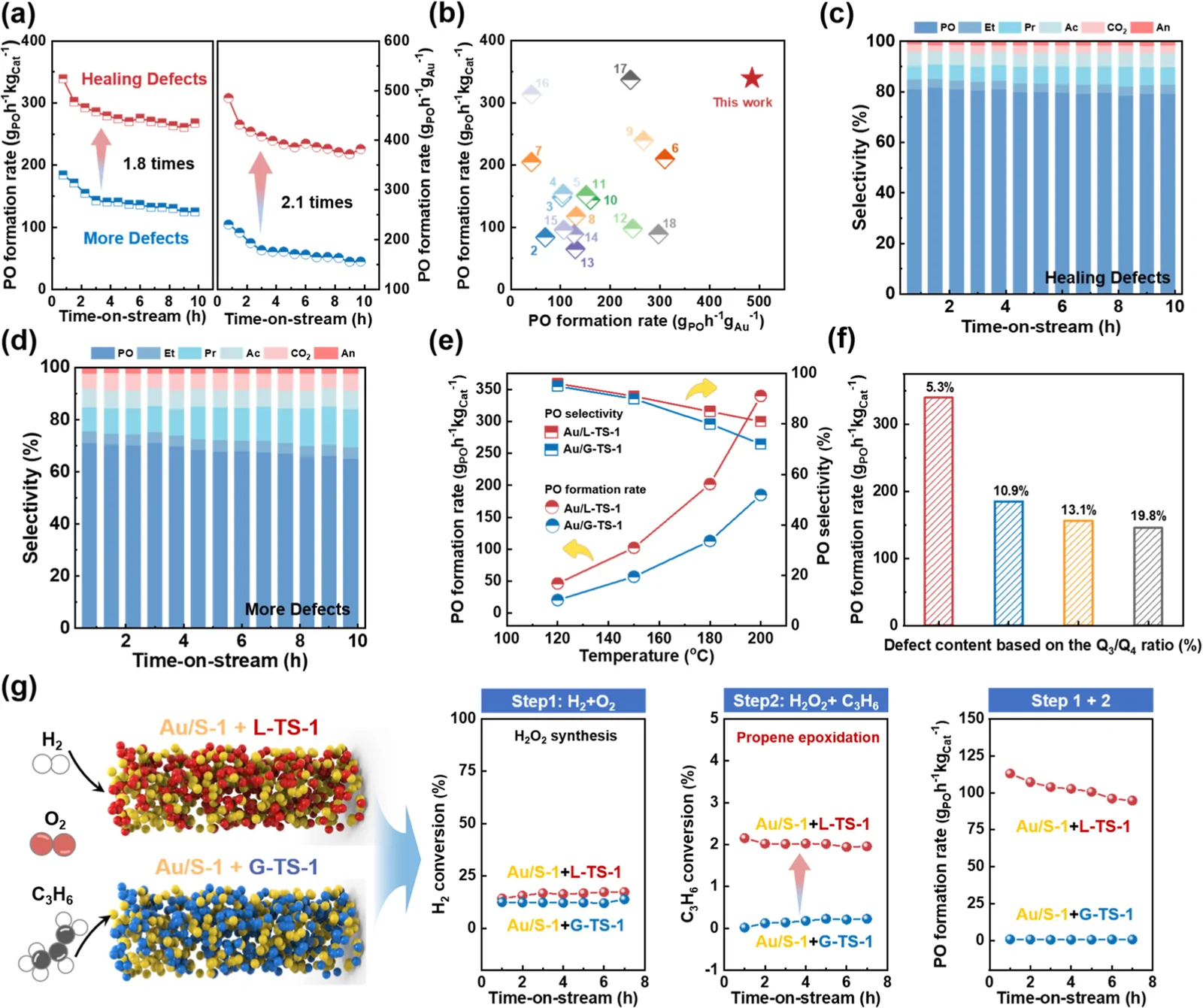
(a) PO formation rate per gram of Au and the PO formation rate per gram of catalyst for Au/L-TS-1 and Au/G-TS-1. (b) Comparison between previously reported catalytic performances without promoters and the present work (denoted by a red star). Reaction selectivity of (c) Au/L-TS-1 and (d) Au/G-TS-1. (e) PO formation rate per gram of catalyst and PO selectivity for Au/L-TS-1 and Au/G-TS-1 at different temperatures. (f) Relationship between defect contents based on the Q_3_/Q_4_ ratio and PO formation rate of tandem propene epoxidation with H_2_ and O_2_. (g) PO formation rate and H_2_/C_3_H_6_ conversion with mixed Au/S-1 and L-TS-1/G-TS-1.

The reaction temperature was further varied to evaluate the effect of the hydroxyl defects of TS-1 on propene epoxidation with H_2_ and O_2_. As the reaction temperature increased from room temperature to 200 °C, PO formation was undetectable below 100 °C, above which the formation rate gradually increased (Figure 2e). Across the entire temperature range of 100–200 °C, Au/L-TS-1 consistently exhibited a higher catalytic performance, with PO formation rates increasing from 47 to 340 g_PO_ h^–1^ kg_Cat_^–1^, compared to 21 to 185 g_PO_ h^–1^ kg_Cat_^–1^ for Au/G-TS-1. Although PO selectivity decreased with increasing temperature for both catalysts, the decline was significantly less pronounced over Au/L-TS-1 (96% to 81%) than that over Au/G-TS-1 (95% to 72%). This behavior indicates that defect healing in Au/L-TS-1 effectively suppresses temperature-induced side reactions, thereby preserving higher selectivity under elevated-temperature conditions. In addition, the Au/L-TS-1 exhibited a slightly lower apparent activation energy (38 < 42 kJ/mol) compared with Au/G-TS-1, which can be attributed to the defect-healed microenvironment in TS-1 that stabilizes key intermediates and the PO product for a higher PO formation rate (Figure S17). The TS-1 samples synthesized at different crystallization times with varied defect contents were further employed to establish the relationship between the defect content and catalytic performance (Figure 2f). As the framework defect content increased from 5.3% to 19.8% based on the Q_3_/Q_4_ ratio in the ^29^Si MAS NMR spectra (Figure S18), the initial PO formation rate progressively decreased from 340 to 145 g_PO_ h^–1^ kg_Cat_^–1^, indicating that a defect-healed zeolite microenvironment enhances tandem propene epoxidation with H_2_ and O_2_.

Tandem propene epoxidation with H_2_ and O_2_ is governed by three key factors: the ability of Au nanoparticles to generate H_2_O_2_ in situ, the microenvironment influencing the intermediate H_2_O_2_ and the PO product, and the ability of Ti active sites to facilitate the epoxidation reaction using the generated H_2_O_2_.^40–42^ To evaluate the role of Au nanoparticles in the initial H_2_O_2_ formation step, H_2_O_2_ synthesis experiments were performed in the absence of propene. Both Au/L-TS-1 and Au/G-TS-1 catalysts, featuring comparable Au nanoparticle sizes (∼2.0 nm, Figure S19), exhibited similar H_2_O_2_ production rates (∼13 mol_H2O2_·kg_cat_^–1^·h^–1^) and H_2_ selectivities (∼50%) (Figure S20), indicating equivalent activity in H_2_O_2_ generation. Furthermore, ex situ propene epoxidation tests with preformed H_2_O_2_ were conducted to probe the intrinsic activity of the Ti sites, where both catalysts showed comparable H_2_O_2_ conversion (∼10%) and PO formation rates (∼0.73 mmol·h^–1^) (Figure S21), demonstrating similar epoxidation performance. Collectively, these results confirm that both catalysts possess comparable activities in H_2_O_2_ synthesis and epoxidation, thereby excluding intrinsic Au and Ti active site differences and highlighting the dominant role of the transport of oxygen-containing species in the zeolite microenvironment.

Mixtures of Au/S-1 with L-TS-1 and G-TS-1 were further employed to elucidate the role of defect-controlled microenvironments (Figure 2g). It was found that the mixture of Au/S-1 and G-TS-1 exhibited a very low PO formation rate of ∼0.6 g_PO_h^–1^kg_Cat_^–1^, whereas the mixture of Au/S-1 and L-TS-1 achieved a higher rate of ∼100 g_PO_h^–1^kg_Cat_^–1^. Notably, both systems showed comparable H_2_ conversion (∼12%), while the propene conversion over the Au/S-1 + L-TS-1 mixture was markedly higher than that over Au/S-1 + G-TS-1 (2% vs ∼0.2%). These results further demonstrate that, in the absence of a defect-healing microenvironment, the in situ generated H_2_O_2_ cannot be efficiently transported from Au nanoparticles to Ti active sites, thereby severely limiting the tandem catalytic performance.

H_2_O_2_ decomposition experiments show a substantially lower decomposition rate over defect-healed Au/L-TS-1 (5.1%) than over Au/G-TS-1 (10.4%) (Figure S22), further confirming that defect healing effectively suppresses unproductive H_2_O_2_ decomposition. The PO DRIFTS results (Figure S23) reveal stronger adsorption and degradation of PO on defect-rich Au/G-TS-1, thereby leading to a lower PO selectivity. These findings highlight that healing hydroxyl-rich defect sites suppresses the molecular trapping of oxygen-containing species, thereby restraining H_2_O_2_ decomposition and PO ring-opening or overoxidation, ultimately facilitating tandem propene epoxidation.

To gain molecular-level insight into how defect healing modulates molecular trapping in the porous zeolite microenvironment, DFT calculations were performed to investigate the interactions of H_2_O_2_ and PO with defect-containing (TS-1-OH) and defect-healed (TS-1) porous microenvironments. For H_2_O_2_, both the O–O bond (1.473 vs 1.469 Å) and O–H bond (1.019 vs 0.991 Å) are elongated in the defect-rich TS-1-OH (Figure 3a) relative to the defect-healed TS-1, which can be attributed to hydrogen-bonding interactions between H_2_O_2_ and surface hydroxyl groups, with a characteristic H···O distance of 1.766 Å. A similar trapping effect is observed for PO. The C–O bond lengths are also slightly elongated in TS-1-OH (1.453 and 1.469 Å) compared to TS-1 (1.448 and 1.452 Å) in Figure 3b, consistent with hydrogen-bond formation (1.722 Å) between PO and hydroxyl defects. Both H_2_O_2_ (−0.651 eV vs −0.386 eV) and PO (−0.746 eV vs −0.508 eV) exhibit stronger adsorption on hydroxyl-rich TS-1-OH than on defect-healed TS-1 in Figure 3c. These results indicate that hydroxyl-rich defects behave as molecular traps for oxygen-containing intermediates and products.

**Figure 3:**
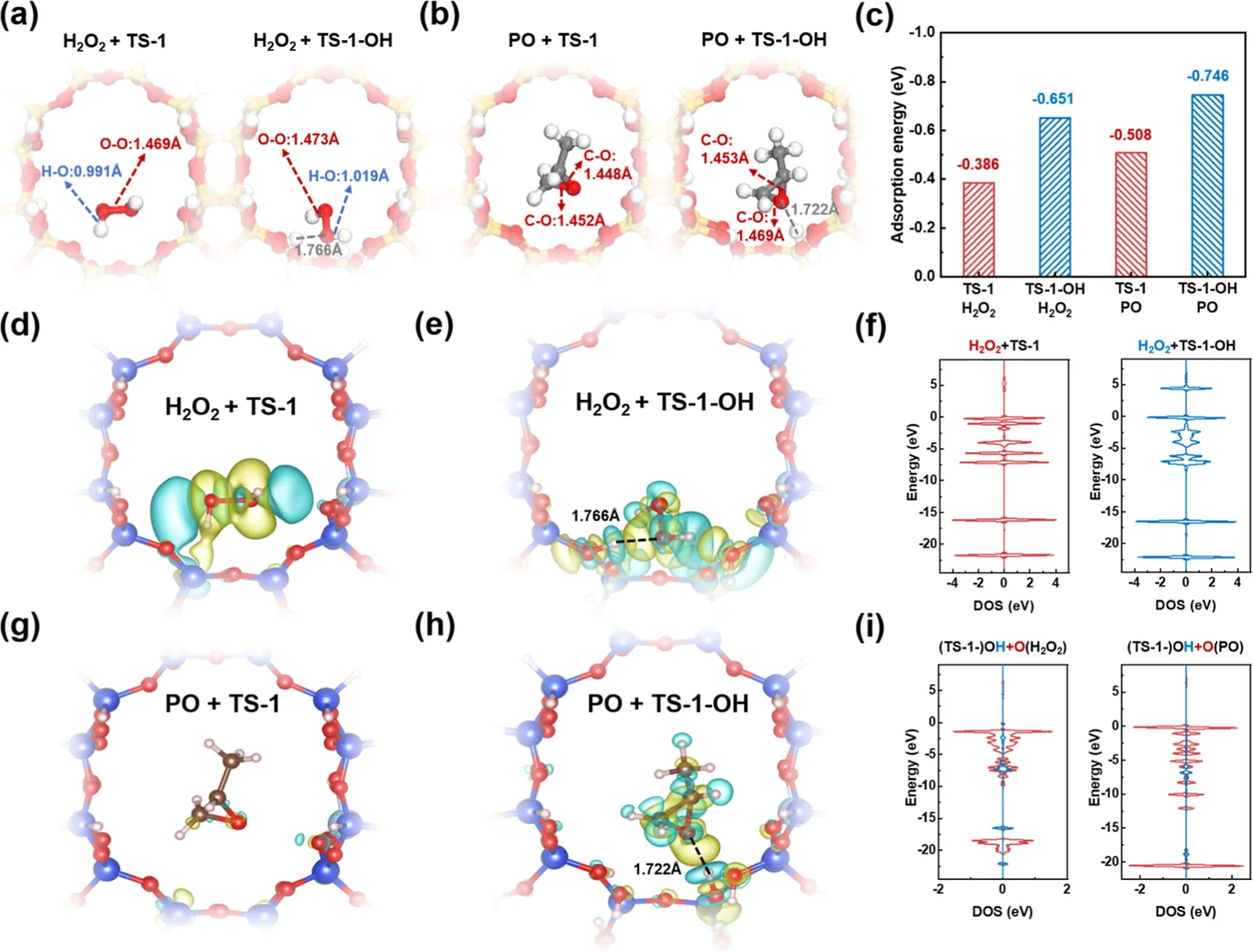
Optimized adsorption configurations and bond lengths of (a) H_2_O_2_ and (b) PO on defect-healed TS-1 and hydroxyl-rich TS-1-OH. (c) Calculated adsorption energies of H_2_O_2_ and PO on defect-healed TS-1 and hydroxyl-rich TS-1-OH. Differential charge density plots of H_2_O_2_ adsorption on (d) defect-healed TS-1 and (e) hydroxyl-rich TS-1-OH, where light blue and yellow isosurfaces represent charge depletion and accumulation (±0.001 e Å^–3^), respectively. (f) Total density of states (DOS) of H_2_O_2_ adsorbed on defect-healed TS-1 and hydroxyl-rich TS-1-OH. Differential charge density plots of PO adsorption on (g) defect-healed TS-1 and (h) hydroxyl-rich TS-1-OH, where light blue and yellow isosurfaces represent charge depletion and accumulation (±0.001 e Å^–3^), respectively. (i) Partial density of states (PDOS) showing interactions between H atoms from TS-1 defect sites and O atoms from H_2_O_2_ and PO.

Electronic structure analyses were further conducted to gain deeper insight into these molecular-trapping mechanisms. Differential charge density plots reveal stronger electron redistribution between H_2_O_2_ and hydroxyl groups than with the defect-healed framework in Figure 3d,e. Bader charge analysis shows that the charges of the two hydrogen atoms from H_2_O_2_ in defect-healed TS-1 (0.366 and 0.390 e) are higher than those in hydroxyl-rich TS-1-OH (0.322 and 0.370 e), further supporting the presence of hydrogen-bonding interactions between the H atom from H_2_O_2_ and the O atom from defects. Moreover, the total density of states (DOS) of H_2_O_2_ on TS-1-OH shifts toward lower energy levels, with more pronounced splitting of bonding orbitals below the Fermi level, indicating stronger interaction between H_2_O_2_ and hydroxyl groups (Figure 3f). Similar behavior is observed for PO adsorption, where enhanced charge accumulation on the oxygen atom (6.928 vs 6.905 e) of PO (Figure 3g,h) and increased DOS peak splitting on TS-1-OH (Figure S24) further confirm stronger PO-defect interactions. Additionally, PDOS analysis shows partial overlap between the H states of hydroxyl groups and the O states of both H_2_O_2_ and PO (Figure 3i), indicating electronic coupling characteristic of hydrogen-bond-mediated molecular trapping.

Taken together, these results demonstrate that hydroxyl-rich defect microenvironments act as molecular traps for both H_2_O_2_ intermediates and PO products through hydrogen-bonding interactions. Such trapping activates H_2_O_2_ toward unproductive decomposition and retains PO in the porous microenvironments, promoting ring opening or overoxidation. In contrast, defect-healed microenvironments simultaneously suppress these undesired interactions originating from coupled pathways in the porous microenvironment of titanosilicalites, stabilizing H_2_O_2_ to facilitate Ti–OOH formation while enabling rapid PO desorption. This dual effect ultimately enhances both Au utilization efficiency (485 g_PO_ h^–1^ g_Au_^–1^) and PO formation rate (340 g_PO_ h^–1^ kg_cat_^–1^), leading to superior tandem catalytic performance.

### Accelerated Defect Healing Mechanism and Sustainability Assessment

To elucidate the mechanism of defect healing induced by thermal-environment modulation, ANSYS Fluent simulations were performed to investigate the thermal environments during TS-1 crystallization in liquid (dimethyl silicone oil, L-TS-1) and gas (air, G-TS-1) media at 170 °C under autogenous pressure. The temperature–time profiles at three representative positions (points I–III) show that oil provides a significantly higher heating rate than air (Figures 4a and S25). This trend is further supported by two-dimensional temperature evolution maps across the tube cross-section (Figures 4b and S26), which clearly illustrate the enhanced heat transfer under liquid conditions. Quantitatively, the average heat-transfer rate prior to reaching 443 K is much higher in oil than in air (λ_L_: 2.84 kJ/s vs λ_G_: 0.71 kJ/s). These results demonstrate that the oil medium substantially improves heat transfer to the titanosilicalite gel, thereby accelerating its heating process.

**Figure 4:**
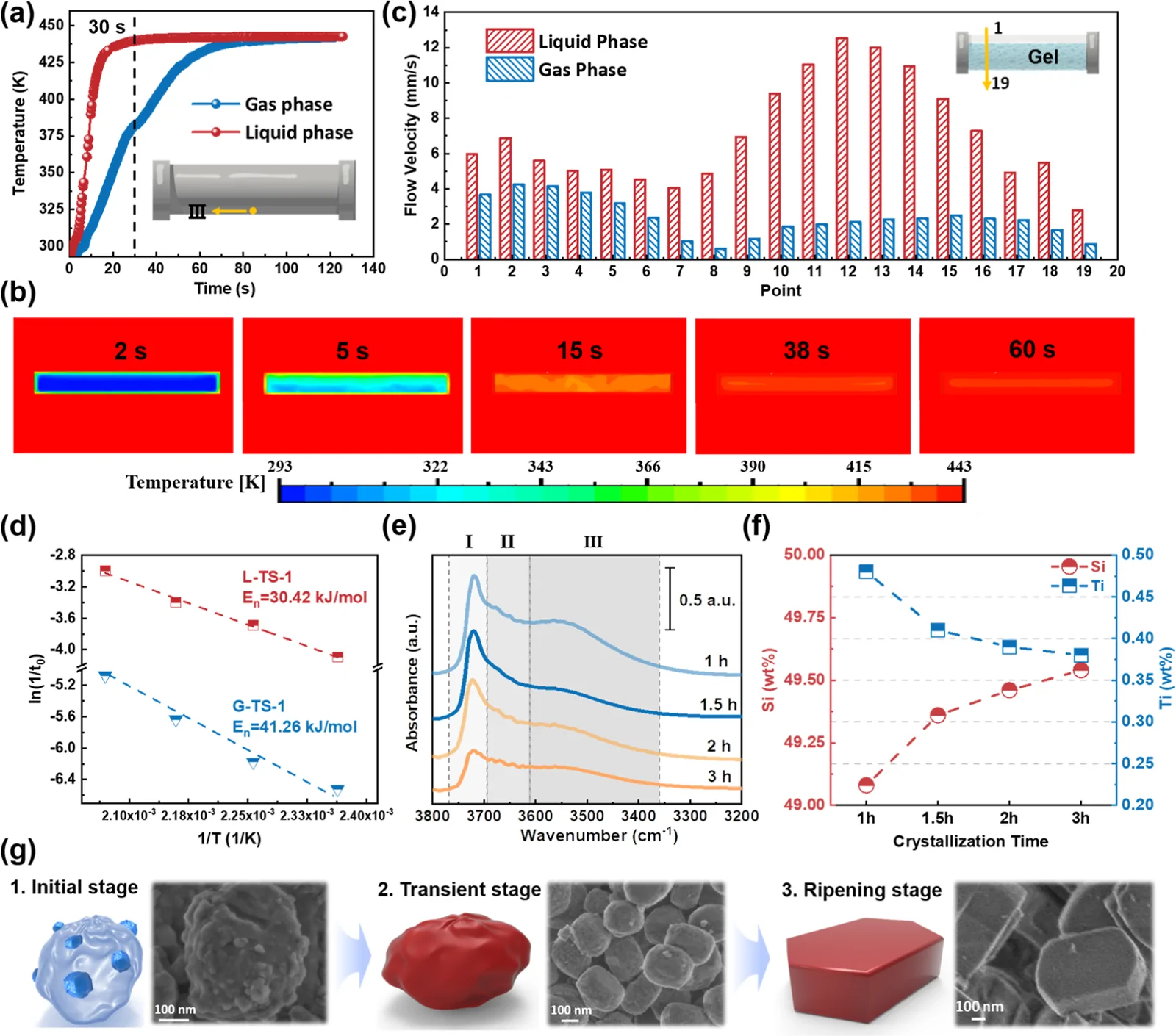
(a) Temperature–time curves in oil (liquid phase) or air (gas phase) medium at point III. (b) Two-dimensional diagram of temperature over time for the cross section of the tube in heat transfer media of oil. (c) Flow velocity at different points in the heat transfer medium of oil and air. (d) Activation energy for the nucleation of G-TS-1 and L-TS-1. (e) FT-IR spectra of titanosilicalites crystallized at different times in the hydroxyl stretching region. The hydroxyl stretching regions I, II and III could be ascribed to the isolated external silanols, internal silanols, and hydrogen-bonded silanol nets, respectively. (f) Ti and Si contents in the titanosilicalite at different crystallization times. (g) SEM images and the schematic diagram of titanosilicalites crystallized after 0.5, 1, and 3 h.

The enhanced heating rate in the liquid-phase environment also accelerates mass transport of Si/Ti species, as evidenced by higher flow velocities of the titanosilicate gel in the stainless-steel tube. Simulations show that flow velocities at all measured positions are consistently higher under oil heating than in air, particularly in the gel-filled region (points 7–18) (Figures 4c and S27). According to classical nucleation theory, the nucleation rate depends on a kinetic prefactor proportional to the self-diffusion coefficient and is exponentially sensitive to temperature via the nucleation energy barrier.^43–45^ Therefore, the combined effect of elevated temperature and accelerated diffusion under liquid-phase heating significantly promotes nucleation and subsequent crystal growth. This is supported by DLS analysis after 30 min of crystallization (Figure S28), where G-TS-1 exhibits a single population (∼6.5 nm), while L-TS-1 shows additional larger species (68 and 1484 nm), indicating enhanced nucleation and aggregation. Consistently, L-TS-1 displays a lower nucleation activation energy than G-TS-1 (30.42 vs 41.26 kJ/mol) in Figure 4d, confirming that the liquid-phase thermal environment facilitates nucleation kinetics and further accelerates the crystallization pathway.

The mechanism of defect healing in L-TS-1 was further elucidated by time-resolved crystallization studies under liquid-phase thermal conditions. XRD analysis (Figures S1 and S29) shows typical diffraction peaks characteristic of the MFI structure, with crystallinity increasing over 3 h. Nitrogen physisorption (Figure S30 and Table S4) reveals that the surface area and micropore volume of L-TS-1 remain relatively stable over the time period studied. UV–vis analysis (Figure S31) confirms the presence of tetrahedrally coordinated Ti sites only, with no anatase species present. FT-IR analysis (Figure 4e) shows a gradual decrease in hydroxyl group intensity over 3 h, indicating the progressive healing of framework defects, including isolated external silanols, internal silanols, and hydrogen-bonded silanol networks. In parallel, NH_3_-TPD demonstrates a decrease in acid site density from 6.24 × 10^–2^ to 4.63 × 10^–2^ mmol/g, further confirming progressive defect healing (Figure S32 and Table S5).

XRF analysis shows an increase in Si content accompanied by a decrease in Ti content with crystallization time (Figure 4f), suggesting that the Ti concentration gradually decreases from the crystal core toward the shell. This structural evolution is supported by DFT calculations (Figures S33 and S34), which indicate that Ti incorporation into the MFI framework is energetically favorable at low substitution levels (up to two Ti atoms) but becomes unfavorable at higher loadings due to framework distortion.^46^ This implies that Ti species are preferentially incorporated during the early stages of nucleation. Cross-sectional SEM-EDS analysis further confirms the formation of a Si-enriched shell (Figure S35), in agreement with the XRF results. SEM imaging (Figure 4g) reveals that the accelerated defect healing proceeds through distinct stages, including particles with small crystallites attached and a highly rough surface (initial stage), followed by the dissolution of these attached particles leading to a less rough surface (transient stage) and ultimately the formation of well-defined crystals with smooth surfaces, resulting from Ostwald ripening (ripening stage). In summary, the enhanced heat transfer efficiency in the liquid-phase environment promotes Brownian motion and mass transport of Si and Ti species, thereby lowering the nucleation barrier. Then, Ostwald ripening facilitates the condensation of surface silanol groups into Si–O–Si linkages, leading to surface reconstruction and effective defect elimination.

This defect-healing strategy was further extended to other zeolite frameworks, including TS-2, zeolite-Y, ZSM-5, and silicalite-2, all of which exhibit a reduction in defect density (Figures 5a and S36–S44). This demonstrates the general applicability of the accelerated defect-healing approach across titanosilicates, aluminosilicates, and pure-silica zeolites. The synthesis was then scaled up to 10 L to evaluate industrial feasibility (Figure 5b). The material shows a similarly low defect content, with a Q_3_/Q_4_ ratio of 5.32% in ^29^Si NMR and a defect density (4.15 × 10^–2^ mmol/g) comparable to that at small scale (Figure S45). TS-1 was obtained with an 80% yield, with no detectable extra-framework Ti species (Figure S46). These results confirm that the strategy enables both general defect elimination for different kinds of zeolites and scalable synthesis.

**Figure 5:**
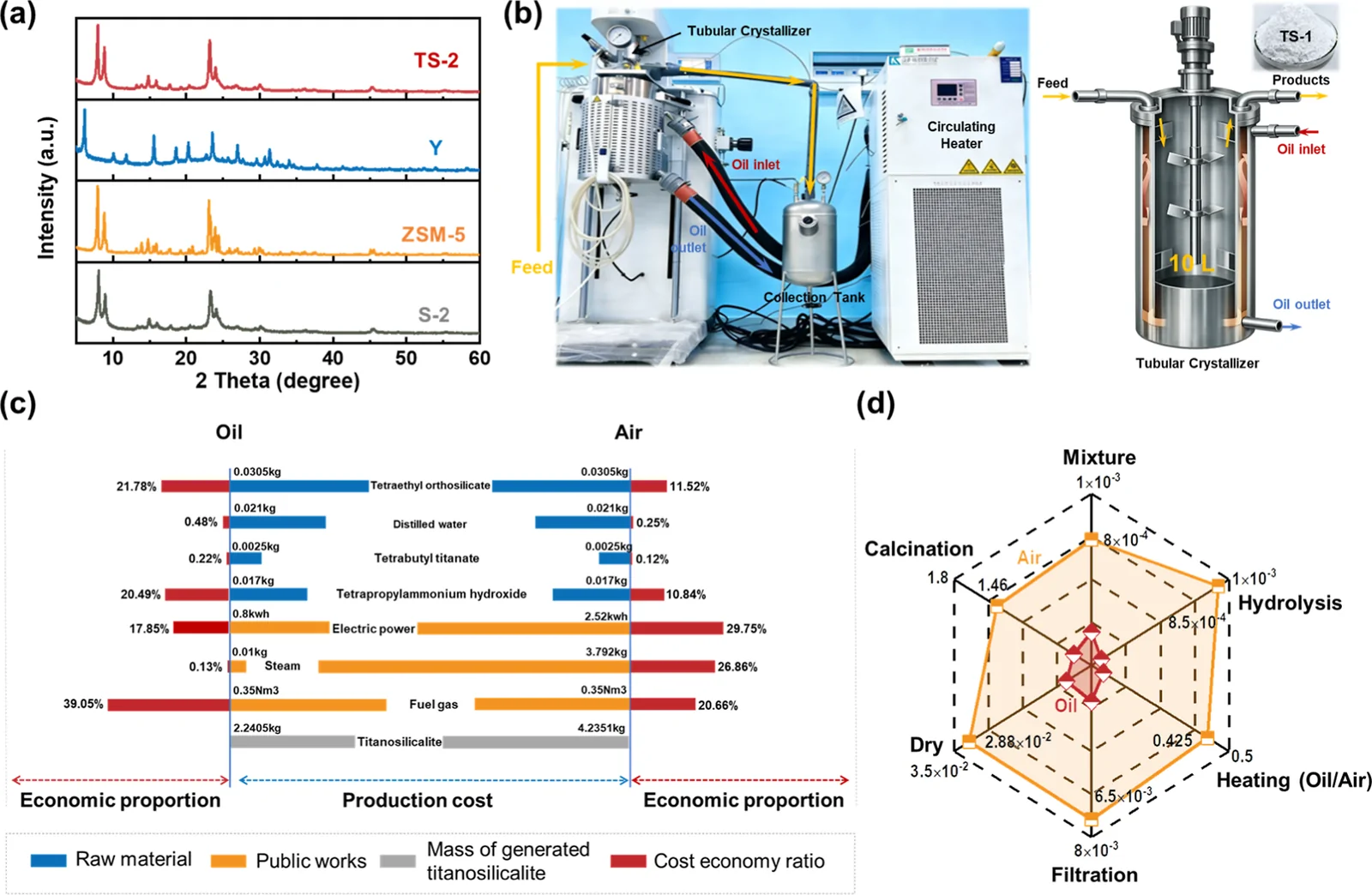
(a) XRD patterns of different zeolites synthesized via the defect-healing strategy. (b) Photograph of the scale-up process for zeolite defect healing and schematic illustration of the 10 L tubular reactor. (c) Production cost analysis and (d) comparison of carbon emissions (kg) for two different routes.

The industrial relevance and environmental impact of the synthesis were evaluated through a comparative techno-economic and carbon-emission analysis of liquid-phase (Route 1, Table S6) and gas-phase (Route 2, Table S7) TS-1 synthesis using Aspen Plus (Figures S47 and S48). The process model included feed preparation, hydrolysis, distillation, crystallization, filtration, drying, and calcination. Both routes were simulated at a TEOS feed rate of 3048 kg/h, equivalent to approximately 24,000 t per year based on 8000 operating hours. TS-1 products with comparable crystallinity were obtained after 3 and 20 h of crystallization for Routes 1 and 2, respectively. Costs and carbon emissions were normalized to one metric ton of TS-1. The cost analysis included raw materials and utilities (electricity, steam, and fuel gas) using identical unit prices for both routes, while carbon emissions were evaluated across the modeled production steps. Route 1 shows significantly lower utility consumption, particularly in electricity and steam, because of accelerated crystallization. Cost analysis (Tables S8 and S9) indicates that Route 1 is dominated by raw material costs, whereas Route 2 incurs higher utility costs arising from prolonged operation in Figure 5c. Overall, Route 1 achieves a lower production cost (10,980.84 vs 13,837.67 USD per ton). Carbon-emission analysis reveals that calcination is the primary contributor in both routes (>70%), while Route 1 exhibits substantially lower total emissions than Route 2 (868 vs 1874 kg/ton), attributed to improved process efficiency in Figures 5d and S49. These results demonstrate that the defect-healing crystallization strategy not only improves material quality for efficient propene epoxidation by suppressing oxygen-containing species trapping but also enhances process efficiency and reduces emissions from an industrial standpoint.

## Conclusion

In summary, an accelerated defect-healed microenvironment in TS-1 is realized through an improved thermal environment, which enhances Brownian motion and intensifies interactions between Si and Ti species. This accelerates nucleation and boosts Ostwald ripening, driving surface reconstruction, and eliminating hydroxyl defects. The elimination of hydroxyl-rich defects reduces oxygen-containing species trapping in the zeolite microenvironment, enabling more efficient transport of in situ-generated H_2_O_2_ between bifunctional active sites and minimizing its unproductive conversion to H_2_O. At the same time, the diminished interaction between PO and residual hydroxyl species promotes rapid product transport, thereby mitigating the ring-opening and overoxidation pathways. The resulting defect-healed Au/L-TS-1 exhibits a 1.8-fold enhancement in the PO formation rate to 340 g_PO_ h^–1^ kg_Cat_^–1^ and a 2.1-fold increase in Au-based productivity to 485 g_PO_ h^–1^ g_Au_^–1^. Notably, this strategy exhibits general applicability in defect reduction across multiple zeolite frameworks (TS-1, TS-2, Y, ZSM-5, and silicalite-2) and can be extended to a 10 L scale while maintaining a comparable defect density. Compared with conventional approaches, this route reduces the production cost by 21% and carbon emissions by 54% per ton of titanosilicalite. Overall, this work highlights defect regulation as a powerful approach for controlling the utilization and transformation of intermediate species in zeolite microenvironments, providing new opportunities for the design of efficient tandem catalytic systems with in situ-generated H_2_O_2_ and the scalable manufacture of high-performance zeolites.

## Supplementary Material



## Notes

Experimental section, sample synthesis, catalytic testing, density functional theory calculation, ANSYS fluent simulation, Aspen Plus V11 simulation, characterization, nitrogen-adsorption-desorption results of titanosilicalites, comparison of catalytic performance, nitrogen-adsorption-desorption results, mass data, technical and economic performance, XRD patterns, ^29^Si MAS NMR spectra, Py-IR characterization, ^1^H MAS NMR spectra, NH_3_-TPD, TEM images, UV-vis spectra, HAADF-STEM, Py-IR spectra, and FT-IR spectra (PDF)

## Notes

The data supporting the findings of this study are provided in the article and its Supporting information. The underlying research data are available through the Cardiff University Research Data Repository at doi.org/10.17035/cardiff.34335897.
