# Supplementary material for: Accelerated Zeolite Defect Healing Suppresses Oxygen-Containing Species Trapping for Tandem Propene Epoxidation
Source: J Am Chem Soc. 2026 Oct 16;148(42):46332–42. doi: 10.1021/jacs.6c17355 (PMC13721175; doi:10.1021/jacs.6c17355)
Supplement: Supplementary file 1 [file ja6c17355_si_001.pdf]

Supporting Information

Accelerated Zeolite Defect Healing Suppresses Oxygen-Containing Species Trapping for Tandem Propene Epoxidation

Dong Lin[a],[b], ‡, Xin Zhou[c], ‡, Yang Xu[a], ‡, Richard J. Lewis[b], *, Zongzhuang Sun[a], Defu Yin[a], Yongxiang Wang[d], Zhenyi Liu[a], Jingyuan Shang[a], Zhe Ma[a], Qiang Wang[d], Feng Deng[d], Chaohe Yang[a], De Chen[e], Graham J. Hutchings[b], * and Xiang Feng[a], *

[a]State Key Laboratory of Heavy Oil Processing, China University of Petroleum, Qingdao 266580, China

[b]Max Planck-Cardiff Centre on the Fundamentals of Heterogeneous Catalysis FUNCAT, Cardiff Catalysis Institute, School of Chemistry, Cardiff University, Cardiff, CF24 4HQ, United Kingdom

[c]College of Chemistry and Chemical Engineering, Ocean University of China, Qingdao 266100, China

[d]National Centre for Magnetic Resonance in Wuhan, State Key Laboratory of Magnetic Resonance and Atomic and Molecular Physics, Wuhan Institute of Physics and Mathematics, Innovation Academy for Precision Measurement Science and Technology, Chinese Academy of Sciences, Wuhan 430071, China

[e]Department of Chemical Engineering, Norwegian University of Science and Technology, Trondheim 7491, Norway

[‡]These authors contribute equally to this work

Corresponding author

Correspondence to: Richard J. Lewis (lewisr27@cardiff.ac.uk); Xiang Feng (xiangfeng@upc.edu.cn); Graham J. Hutchings (hutch@cardiff.ac.uk)

Contents

1. Experimental section
Sample synthesis S4
Catalytic testing S6
Density functional theory (DFT) calculation S7
ANSYS Fluent simulation S7
Aspen Plus V11 simulation S8
2. Characterization S9
3. Supplementary Tables
Nitrogen-adsorption-desorption results of titanosilicalites S11
The relative content of Si/Ti, bulk Si/Ti ratios and Au contents S11
A comparison of catalytic performance towards the in-situ propene epoxidation S12
Nitrogen-adsorption-desorption results of titanosilicalites after different crystallization time S13
The content of acid site in titanosilicalites after different crystallization time S13
Mass data of synthesizing titanosilicalites using oil heating derived from Aspen Plus V11 S14
Mass data of synthesizing titanosilicalites using air heating derived from Aspen Plus V11 S14
Technical and Economic Performance of Route 1- Production Cost Analysis S15
Technical and Economic Performance of Route 2- Production Cost Analysis S16
4. Supplementary Figures
The XRD patterns of titanosilicalites crystallized in the oil (L-TS-1) S17
The XRD patterns of titanosilicalites crystallized in the air (G-TS-1) S18
The ^{29}Si MAS NMR spectra of (a) G-TS-1 and (b) L-TS-1 titanosilicalites S19
The Py-IR characterization of L-TS-1 S20
The Py-IR characterization of G-TS-1 S21
The ^{1}H MAS NMR spectra of L-TS-1 and G-TS-1 titanosilicalites S22
The NH_3-TPD of L-TS-1 and G-TS-1 titanosilicalites S23
The typical TEM images of (a) (b) G-TS-1 and (c) (d) L-TS-1 S24
The UV-vis spectra of different titanosilicalites S25
UV-vis spectra of Ti species under UV irradiation S26
EPR spectra of titanosilicalite synthesis gel containing the spin-trapping agent DMPO under UV irradiation S27
The HAADF-STEM mapping of G-TS-1 S28
The HAADF-STEM mapping of L-TS-1 S29
Typical HRTEM images of (a) L-TS-1 and (d) G-TS-1. Line intensity profiles corresponding to (a) and (d) are shown in (b) and (e), respectively. SAED patterns of (c) L-TS-1 and (f) G-TS-1 S30
Experimental and calculated XRD patterns of (a) L-TS-1 and (b) G-TS-1 after crystallization at 170 ^{o}C. The unit cell of the (c) L-TS-1 and (f) G-TS-1 calculated by the Rietveld refinement S31
The hydrogen efficiency of different titanosilicalites in the propene epoxidation with H_2 and O_2 S32
Comparison of activation energies for Au/L-TS-1 and Au/G-TS-1 S33
The ^{29}Si MAS NMR spectra of TS-1 synthesized in air after crystallization for (a) 24 h and (b) 36 h S34
The typical STEM images of (a) Au/G-TS-1 and (b) Au/L-TS-1 S35
The (a) productivity of H_2O_2, (b) conversion of H_2 and selectivity of H_2O_2 by direct synthesis between H_2 and O_2 on different Au/TS-1 catalysts S36
The (a) conversion of H_2O_2, PO selectivity and (c) PO formation rate by ex-situ propene epoxidation on different Au/TS-1 catalysts S37
The degradation of H_2O_2 on different Au/TS-1 catalysts S38
The PO DRIFT on different Au/TS-1 catalysts S39

Total density of states (DOS) of PO adsorbed on (a) defect-healed TS-1 and (b) hydroxyl-rich TS-1-OH S40
The temperature-time curves in oil (liquid phase) or air (gas phase) medium at different representative points (a) I, (b) II S41
The two-dimensional diagram of temperature over time for the cross section of the tube in heat transfer media of air S42
The two-dimensional diagram of flow velocity after heating 5 s for the cross section of the tube in the heat transfer media of (a) oil and (b) air S43
The dynamic light scattering-particle size distributions (DLS-PSD) of G-TS-1 and L-TS-1 at the crystallization time of 30 minutes S44
The relative crystallinity of titanosilicalites crystallized at different time in the heat transfer medium of oil S45
The nitrogen adsorption and desorption isotherms of titanosilicalites crystallized at (a) 1 h, (b) 1.5 h, (c) 2 h and (d) 3 h in the heat transfer medium of oil S46
The UV-vis spectra of titanosilicalites crystallized at different time in the heat transfer medium of oil S47
The NH_3-TPD patterns of titanosilicalites crystallized at different time in the heat transfer medium of oil S48
The MFI cluster with (a) one, (b) two, (c) three, (d) four and (e) five Ti atoms in the framework S49
The substitution energy of different numbers of Ti atoms in the MFI framework. The inset shows the 12 inequivalent T sites of Si atoms in the MFI framework, represented by different colored spheres S50
(a) The cross-section SEM images of titanosilicalites after crystallization of 3 h. (b) The SEM-EDS line-scan analysis of titanosilicalites after crystallization of 3 h S51
The XRD patterns of different titanosilicalite-2 (L-TS-2 crystallized in the heat transfer medium of oil for 3 h, G-TS-2 crystallized in the heat transfer medium of air for 72 h) S52
The FT-IR spectra of titanosilicalite-2 (L-TS-2 crystallized in the heat transfer medium of oil for 3 h, G-TS-2 crystallized in the heat transfer medium of air for 72 h) in the hydroxyl stretching region S53
The Py-IR spectra of titanosilicalite-2 (L-TS-2 crystallized in the heat transfer medium of oil for 3 h, G-TS-2 crystallized in the heat transfer medium of air for 72 h) S54
The XRD patterns of different Y zeolites (L-Y crystallized in the heat transfer medium of oil, G-Y crystallized in the heat transfer medium of air) S55
The FT-IR spectra of Y zeolites (L-Y crystallized in the heat transfer medium of oil, G-Y crystallized in the heat transfer medium of air) in the hydroxyl stretching region S56
The XRD patterns of different ZSM-5 zeolites (L-ZSM-5 crystallized in the heat transfer medium of oil, G- ZSM-5 crystallized in the heat transfer medium of air) S57
The FT-IR spectra of ZSM-5 zeolites (L-ZSM-5 crystallized in the heat transfer medium of oil, G-ZSM-5 crystallized in the heat transfer medium of air) in the hydroxyl stretching region S58
The XRD patterns of different silicalite-2 (L-S-2 crystallized in the heat transfer medium of oil, G-S-2 crystallized in the heat transfer medium of air) S59
The FT-IR spectra of silicalite-2 (L-S-2 crystallized in the heat transfer medium of oil, G-S-2 crystallized in the heat transfer medium of air) in the hydroxyl stretching region S60
The (a) ^{29}Si MAS NMR and (b) NH_3-TPD of the scale-up titanosilicalite S61
The (a) XRD and (b) UV-vis spectrum of the scale-up titanosilicalite S62
Flow diagrams of two routes for synthesizing TS-1 based on oil or air heating S63
The detailed process simulation flowsheet of synthesizing titanosilicalites by Aspen Plus V11 S64
Comparison of carbon emission percentages for different steps in two different routes S65

1. Experimental Section

Sample synthesis

For the synthesis of titanosilicalite-1 (TS-1), 30.48 g of tetraethyl orthosilicate, 17.00 g of tetrapropylammonium hydroxide (25 wt%), 0.25 g of tetrabutyl titanate, and 21.00 g of deionized water were mixed directly and under irradiation by ultraviolet (500w, mercury lamp) for 1h, with stirring at 500 r/min at room temperature. The obtained sol gel was crystallized at 170 °C with a specific time. The sol gel was heated by air or dimethicone oil in a stainless-steel tube with an external diameter of 12 mm, a thickness of 1.5 mm, and a length of 10 cm. The heating medium, which could be either air or oil, was preheated to 170 °C. After crystallization, the as-synthesized TS-1 was separated from the mother liquor by centrifugation and washed with deionized water of 30 mL for three times. Then these samples were dried at 80 °C for 8 h and calcined at 550 °C with a speed of 2°C/min for 6 h in static air. The obtained white powders heated by dimethicone oil and air were referred as L-TS-1 and G-TS-1, respectively. In a typical synthesis of Au/TS-1, 4.50 g of deionized water was mixed with 0.009 g of chloroauric acid ($HAuCl_4 \cdot 3H_2O$, 99.99%), and the solution of sodium carbonate (1M and 0.1M) was added dropwise into the Au-containing solution until the pH reached 7.3-7.6 for 0.5h. Then deionized water was added dropwise to ensure that the volume of solution reached 5 mL. After that, the 0.4 mL of the above-mentioned solution was added into 0.5 g of TS-1 and dried in vacuum oven at room temperature for 8 h.

For the synthesis of TS-2, 30.45 g of tetraethyl orthosilicate, 17.00 g of tetrapropylammonium hydroxide (25 wt.%), 0.25 g of tetrabutyl titanate, and 21.45 g of deionized water were mixed directly and under irradiation by ultraviolet (500w, mercury lamp) for 1h, with stirring at 500 r/min at room temperature. The obtained sol gel was crystallized at 170 °C. The sol gel was heated by air or dimethicone oil in a stainless-steel tube with an external diameter of 12 mm, a thickness of 1.5 mm, and a length of 10 cm. After crystallization, the as-synthesized TS-2 was separated from the mother liquor by centrifugation and washed with deionized water (3×30 mL). The sample was then dried at 80 °C for 8 h and calcined at 550 °C with a speed of 2°C/min for 6 h in static air. The obtained white powder, heated by dimethicone oil and air were referred as L-TS-2 and G-TS-2, respectively.

For the synthesis of ZSM-5, a total of 31.74 g of tetrapropylammonium hydroxide (TPAOH, 25 wt.%) were added to a 500 mL beaker, followed by 2.76 g of deionized water. The mixture was stirred in a water bath at 35 °C for 20 minutes (500 r/min). Subsequently, 33.45 mL of tetraethyl orthosilicate (TEOS) was added dropwise to the TPAOH solution. After the addition was complete, the mixture was maintained at 35 °C for 2 h until a clear and transparent solution was

obtained, indicating complete hydrolysis of TEOS. In parallel, 0.824 g of aluminum isopropoxide was dissolved in 75 g of deionized water and hydrolyzed at 85 ^{o}C in a water bath for 1.5 h to yield a clear aluminum precursor solution. The hydrolyzed aluminum solution was then added dropwise to the silicon precursor solution. The resulting mixture was vigorously stirred at 35 ^{o}C for 1 hour and 40 minutes. The mixture was then heated to 85 ^{o}C and maintained at this temperature for 2 h to remove ethanol and other alcohols formed during hydrolysis, while deionized water was periodically added to maintain a total liquid volume of approximately 150 mL. The resulting sol-gel was transferred into a stainless-steel tube (12 mm outer diameter, 1.5 mm wall thickness, 10 cm length) and heated at 170 ^{o}C using either air or dimethicone oil to induce crystallization. Upon completion, the reactor was cooled to room temperature. The solid product was collected by centrifugation, washed thoroughly with deionized water three times (3×30 mL), and dried in an oven at 100 ^{o}C for 12 h. The dried sample was calcined in a muffle furnace at 550 ^{o}C for 6 hours with a heating rate of 2 ^{o}C/min. The resulting white powders obtained using dimethicone oil and air as heating media were denoted as L-ZSM-5 and G-ZSM-5, respectively.

For the synthesis of Y zeolite, 41 g of deionized water was added to a 250 mL beaker and stirred. Subsequently, 3.73 g of sodium aluminate was added and dissolved under stirring, followed by the addition of 3.64 g of sodium hydroxide. The mixture was stirred for an additional 30 minutes before 28.64 g of silica sol (30 wt.%) was introduced. Stirring was continued for 1.5 h to ensure homogeneity. The resulting sol-gel was transferred into a stainless-steel tube (12 mm outer diameter, 1.5 mm wall thickness, 10 cm length) and heated at 100 ^{o}C using either air or dimethicone oil to induce crystallization. After crystallization, the system was cooled to room temperature, and the solid product was collected by centrifugation, thoroughly washed with deionized water, and dried to obtain NaY zeolite. Ion exchange was performed by treating 1 g of NaY zeolite with 10 mL of 1 mol/L ammonium chloride solution at 60 ^{o}C for 1.5 h; this process was repeated three times. The exchanged product was dried and then calcined at 550 ^{o}C for 6 h to yield the final HY zeolite. The resulting white powders obtained using dimethicone oil and air as heating media were designated as L-Y and G-Y, respectively.

The precursor solution was prepared by adding 393.4 g of TPAOH solution (25 wt%) into 496 g of deionized water under stirring (Solution A). A designated amount of tetrabutyl titanate (TBOT) was slowly added into 704 g of tetraethyl orthosilicate (TEOS) to form Solution B, which was then rapidly mixed with Solution A to obtain a homogeneous mixture. The resulting solution, together with 1500 g of water, was pumped into a pilot-scale pretreatment unit and aged for ~1 h to yield a clear solution. The aged solution was fed into a pilot-scale crystallizer (~10 L) and crystallized at 170 °C for 3

h under stirring (200 rpm) using a silicone-oil heating system. The product was recovered, washed, dried (90 °C, 12 h), and calcined at 550 °C for 5 h to obtain TS-1.

Catalytic testing

The Au/L-TS-1 and Au/G-TS-1 catalysts were tested in the propene epoxidation with H_2 and O_2 at 200 °C with a space velocity of 14000 $mL \cdot h^{-1} \cdot g_{Cat}^{-1}$. The quartz tubular reactor, shown in Figure S49, has an outer diameter of 10 mm and an inner diameter of 8 mm. The reactants contain propene (3.5 mL/min), oxygen (3.5 mL/min), hydrogen (3.5 mL/min), and nitrogen (24.5 mL/min). 0.15 g of catalysts were loaded into the silica tube and further heated from room temperature to 200 °C with a rate of 1 °C/min under the atmosphere of H_2 and N_2, followed by the introduction of feed gas at 200 °C. C_3 moles refers to the molar amount of species containing three carbon atoms. The calculations of hydrogen efficiency, selectivity, and conversion were based on the following equations:

Hydrogen efficiency:

$$=\frac{\text{propylene oxide moles}}{\text{H}_2\text{ moles in the feed - H}_2\text{ moles in the product}}\times 100\%$$

Conversion of propene:

$$=\frac{\text{C}_3\text{ moles + (acetaldehyde moles}\times 2)/3 + \text{(carbon dioxide moles)/3}}{\text{propene moles}}\times 100\%$$

Selectivity of propylene oxide:

$$=\frac{\text{propylene oxide moles}}{\text{C}_3\text{ moles + (acetaldehyde moles}\times 2)/3+\text{(carbon dioxide moles)/3}}\times 100\%$$

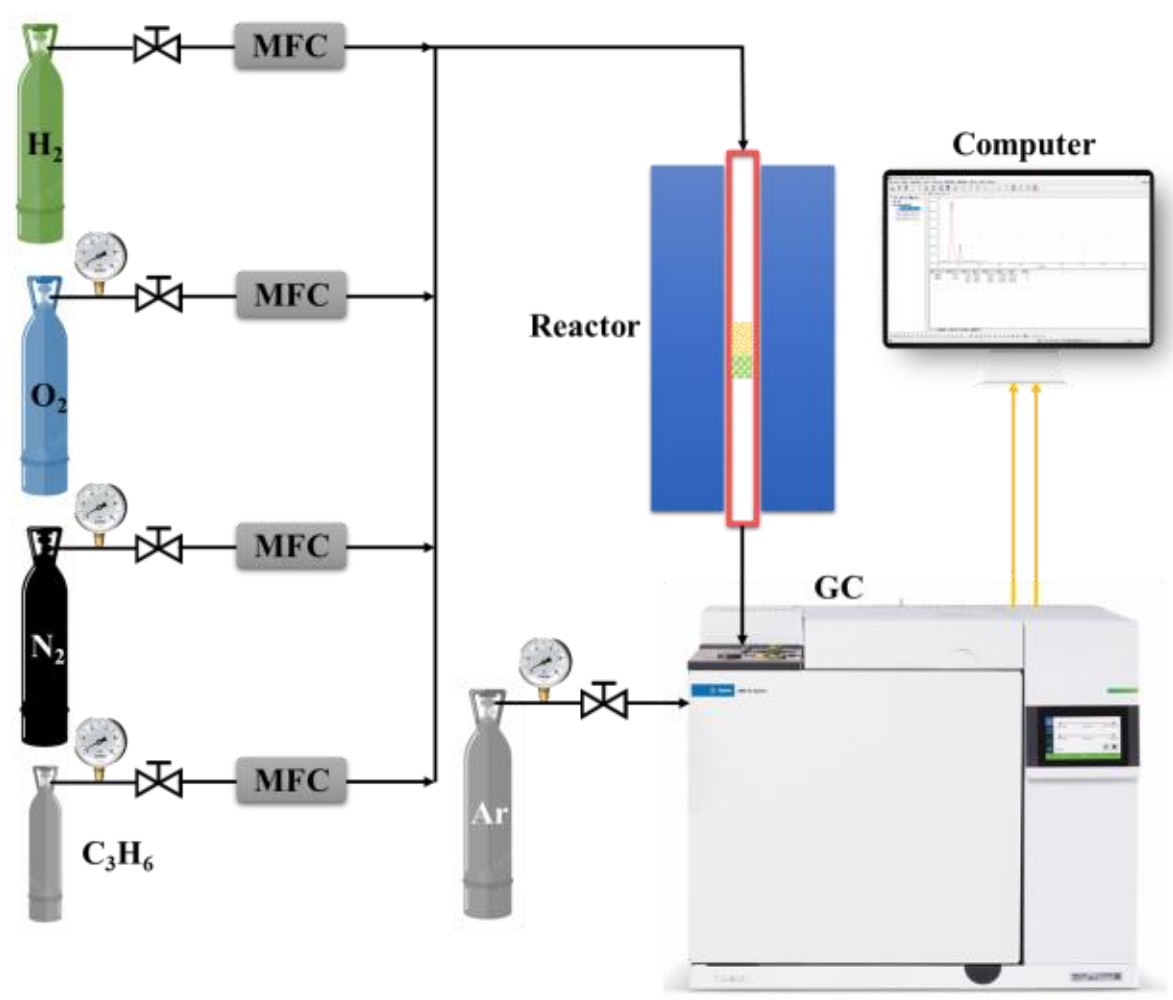

Figure S0 Schematic diagram of the equipment for the propene epoxidation with H_2 and O_2.

The synthesized TS-1 catalyst was evaluated for H_2O_2 decomposition in a 50 mL microreactor equipped with magnetic

stirring. The reaction conditions included 0.2 g of catalyst, 1.6 g of H_2O_2 (30 wt.%), and 20 mL of acetonitrile (CH_3CN), with the reaction conducted at 60°C for 0.5 hours. Upon completion, the microreactor was rapidly cooled to room temperature using an ice-water bath. The remaining H_2O_2 concentration was quantified using the iodometric method. Specifically, 0.30 g of the reaction mixture was weighed, followed by the addition of 3 mL of a 20 wt.% H_2SO_4 solution and 1 mL of saturated ammonium molybdate solution. After thorough mixing, 4 mL of a 10% KI solution was added. The container was sealed and kept in a light-proof environment for 20 minutes. Subsequently, 2 mL of starch solution was introduced as an indicator, and the mixture was titrated with a 0.1 M $Na_2S_2O_3$ aqueous solution until a discernible colour change signified the endpoint.

Density functional theory (DFT) calculation

All density functional theory (DFT) calculations were based on the Vienna ab initio simulation (VASP). The projected augmented wave potentials were used to describe the relationship between the core and valence electrons. The plane-wave basis sets were further used to expand Kohn-Sham orbitals. The generalized gradient approximation (GGA) Perdew-Burke-Ernzerhof (PBE) was used as the functional with a cut-off energy of 400 eV. The k points were set as 1×1×1 in the Brillouin zone. The convergence criterions of force and energy were 0.03 eV/Å for ion relaxation and 1×10^{-5} eV for electronic relaxation. The dispersion effects were considered by using the DFT-D3 method to correct the energy. A 62 T ($Si_{62-n}Ti_nO_{92}H_{64}$) zeolite model was construed based on the MFI zeolite structure. The H atoms were used to coordinate the terminal Si/Ti atoms, and these H atoms had consistent orientations with the replaced O atoms. The zeolite model was optimized when the terminal H atoms were fixed, and other Si/O/Ti atoms were released. The Ti atoms were used to replace the Si atoms at 12 different T sites to construct the TS-1 model with different Ti contents.

ANSYS Fluent simulation

ANSYS Fluent was used to simulate heat transfer process at different media. The stainless-steel tube with a length of 10 cm and external diameter of 12 mm was constructed according to experiment. Due to the large amounts of water and ethanol present in the titanosilicalite gel, a mixture of water and ethanol corresponding to the composition of the titanosilicalite gel, was prepared and heated using air or dimethicone oil to simulate heat transfer during the crystallization process. The multiphase flow module VOF and Evaporation-Condensation were used to realize the phase change process of water and ethanol in a stainless-steel tube heated by air and dimethicone oil. ANSYS Fluent Transient option was chosen, and Gravitational Acceleration gravity direction was defined, then the energy control equation started. In order to calculate the multiphase flow, the Multiphase Model (VOF) module of ANSYS Fluent was selected and Explicit was

used to implement the Geo-Reconstruct discretization method for higher accuracy. The Evaporation-Condensation module was chosen for the phase-change mechanism. The Evaporation-Condensation could be used to simulate the transfer from liquid mass to gaseous state, and Lee Model could import the phase change into ANSYS Fluent with a setting of saturation temperatures (water: 373.15 K and ethanol: 351.15K).

$$\lambda_G = \frac{\int_0^t E_G(t)dt}{t-0} \tag{1}$$

$$\lambda_L = \frac{\int_0^t E_L(t)dt}{t-0} \tag{2}$$

where $E_L(t)$ and $E_G(t)$ represent the system energy in the transfer heat transfer media as a function of crystallization time, respectively. The $E_L(t)$ and $E_G(t)$ were simulated using ANSYS Fluent software. The t is the crystallization time after reaching 443 K.

Aspen Plus V11 simulation

Aspen Plus software was utilized to simulate the process for two different heating methods, both of which were based on comparable crystallinity levels. The simulation calculations were based on the PENG-ROB physical property method. The simulation was scaled up proportionally according to experimental data, with tetraethyl orthosilicate (3048 kg/h) and water (2100 kg/h) as the main raw material inputs. The RStoic reaction module was used to simulate the orthosilicate conversion, with the conversion rate of the tetraethyl orthosilicate component set to 0.99. The RadFrac module was utilized as the purification unit, with a reflux ratio of 0.6 and a distillate feed ratio of 0.2. The guessed distillate rate at the top of the column was 98%.For the hydrolysis unit, the RStoic module was also used, with a conversion rate set to 0.66. HeatX was applied to simulate two heating scenarios: oil bath heating and air heating. The heat loads for these two methods were 1665.50 MJ/hr and 1252.64 MJ/hr, respectively.

2. Characterization

The X-ray diffraction (XRD) analysis of all samples was conducted using an X'pert PRO MPD diffractometer (PANalytical, Netherlands) with Cu Kα radiation (40 kV, 40 mA) in Bragg-Brentano geometry. The TOPAS-Academic V6 software was used to calibrate all X-ray diffraction peaks. The Rietveld refinement was further used to fit the experimental data to obtain related parameters. The lattice parameters were calculated by the Rietveld refinement using the powder X-ray diffraction (XRD) data. The sample with the highest degree of crystallization was selected as the reference and assigned a relative crystallinity of 100%. The relative crystallinity of other samples was determined by comparing the total integrated intensity of five characteristic diffraction peaks within the 2θ range of 5°-35° against that of the reference. The calculation of activation energy for the nucleation was performed using the following equation:

$$\ln(1/t_0) = \ln A - E_n/RT$$

where t_0 is the induction period, A is the pre-exponential factor for the nucleation stage, R is the universal gas constant, and T is the absolute temperature. The ultraviolet visible (UV-vis) spectroscopy was performed on UV-2700 to characterize the coordination mode of Ti species with a background of pure $BaSO_4$. The reaction intermediate of Ti-OOH at various temperatures was unveiled through in situ spectroscopy using the AvaSpec-ULS4096CL-EVO from AVANTES. The spectra were calibrated with the aid of the commercial diffuse reflection reference containing Teflon supplied by AVANTES. The Fourier transform infrared spectroscopy (IS 30, FT-IR) was used to record the structural information of titanosilicalites with a reference of KBr. To ensure analysis of the hydroxyl region was not influenced by the presence of physiosorbed species, the titanosilicalites was pre-treated at 500°C for 1 h under vacuum condition. The sample mass was strictly controlled to 25 mg and the sample was analysed under the same conditions as those outlined above. Pyridine infrared spectroscopy (Py-IR) was used to characterize the Brønsted and Lewis acid sites. The sample was compressed into sheets using a tablet press and subsequently placed in an in-situ infrared cell. The sample mass was strictly controlled to 25 mg. After treatment at 500°C for 1 hour, background spectra at various temperatures were recorded. Pyridine was then adsorbed onto the sample under vacuum conditions at room temperature for 45 minutes. The sample was then heated to various temperatures, and the Py-IR spectra were recorded. Diffuse reflectance infrared Fourier transform (DRIFT) spectra were recorded on a Nicolet iS20 FT-IR spectrometer equipped with an MCT-A detector, KBr windows, and a HARRICK in situ reaction chamber. Approximately 0.2 g of sample was loaded into the chamber. Prior to PO adsorption, the sample was pretreated in situ under a flow of N_2 (30 mL/min) at 200 °C for 1 h. The background spectrum was collected under continuous N_2 flow. Subsequently, a gas stream of N_2 (30 mL/min) bubbled through PO was introduced into the chamber, until no further changes were observed. After adsorption equilibrium was reached, pure N_2 was reintroduced into the chamber for varying durations until the PO-related signals

became stable. The Micromeritics ASAP 2460 was used to determine textural properties of the titanosilicalites. The samples were pre-treated at 300°C for 6 h prior to nitrogen absorption at -196 °C. The bulk content of Ti/Si elements was given by the X-ray fluorescence (EDX-7000, XRF). The size and morphology of titanosilicalites were observed by the high-resolution transmission electron microscope (JEM-2100UHR) and scanning electron microscope (JSM7900F). For the cross-sectional view of the titanosilicalites, the samples were embedded in epoxy resin, and after the resin had hardened, they were sectioned to obtain precise cross-sections. The small crystals observed in the image are not part of the titanosilicates but are artifacts originating from the resin used to embed the sections. The temperature programmed desorption of NH_3 was tested on the Micromeritics Autochem II 2920. The sample was preheated to 200°C in a helium (He) atmosphere. After cooling to 50°C, ammonia (NH_3) was introduced and allowed to adsorb until saturation. The sample was then purged with He for 30 minutes to remove both free and mechanically adsorbed NH_3. Finally, the catalyst was heated to 650°C at a rate of 10°C/min, and the signal was recorded throughout the process. The Si chemical environment was recorded on the ^{29}Si magic angle spinning nuclear magnetic resonance spectroscopy (Varian Unity/Inova). The particle size distribution in liquid was determined by the dynamic light scattering (Malvern Zetasizer Nano ZS90, DLS). 1D one-pulse ^{1}H MAS NMR and 2D ^{1}H-^{1}H DQ-SQ MAS NMR experiments were performed on a 9.4T Bruker AVANCE-III spectrometer using 4 mm rotors at a spinning frequency of 12.5 kHz (operating at a Larmor frequency of 399.3 MHz for 1H). The one-pulse ^{1}H MAS NMR spectra were collected with a pulse width of 3.8 μs corresponding to a π/2 flip-angle, a recycle delay of 3.0 s, and a total of 384 scans. To ensure reliable quantitative comparison across different samples, all ^{1}H MAS NMR measurements were performed under strictly consistent conditions, including identical sample loading, rotor dimensions, spinning rate, and pulse sequence settings. In addition, all samples were evacuated at 80 °C overnight to remove adsorbed water. Furthermore, sufficiently long relaxation delays were applied to achieve accurate signal quantification of the proton species. The 2D ^{1}H-^{1}H DQ-SQ MAS NMR experiments were collected with a REDR (radio frequence-driven recoupling) recoupling pulse and recoupling time of 1.28 ms was used, a total of 80 scans were collected for each of the 40 μs rotor-synchronized t1 increments with a recycle delay of 1.0 s.

2. Supplementary Tables

Table S1 Nitrogen-adsorption-desorption results of titanosilicalites.

	L-TS-1	G-TS-1
[a]S_{BET}/m^2g^{-1}	448.9	450.8
[b]Total pore volume of pores/cm^3g^{-1}	0.22	0.21
[c]Micropore volume/cm^3g^{-1}	0.19	0.20

[a]The surface area was determined by BET model. [b]Total pore volume of pores were determined by single point method.

[c]Micropore volume was determined by t-Plot method.

Table S2 The relative content of Si/Ti, bulk Si/Ti ratios and Au contents.

	SiO_2 (wt%)	TiO_2 (wt%)	Si/Ti molar ratio[a]	Au loading (wt%)[b]
Au/L-TS-1	99.32	0.66	201	0.07
Au/G-TS-1	98.84	0.63	207	0.08

[a]The bulk Si/Al ratios were determined by XRF analysis.

[b]The bulk Au loadings were determined by ICP analysis of digested samples.

Table S3 A comparison of catalytic performance towards the in-situ propene epoxidation.

	Catalyst	PO formation rate ($g_{PO}h^{-1}g_{Au}^{-1}$)	PO formation rate ($g_{PO}h^{-1}kg_{Cat}^{-1}$)	Reference
1	Au/L-TS-1	485	340	This work
2	0.122Au/TS-1(99)	69	84	J. Catal. 2012, 287, 178-189
3	0.143Au/TS-1(99)	103	148	J. Catal. 2012, 287, 178-189
4	0.146Au/TS-1(99)	105	154	J. Catal. 2012, 287, 178-189
5	Au/HTS-1(NIMG)	150.3	150.3	ACS Sustainable Chem. Eng. 2022, 10, 9515-9524
6	0.23Na-Au/TS-1	310	210	Appl. Catal. B: Environ. 2022, 319, 121837
7	Au-Ti@MFI	41	205	J. Mater. Chem. A 2020, 8, 4428-4436.
8	Au/TS-2-B	131	118	AIChE J. 2020, 66, e16815
9	Au/TS-1-B	267	240	Ind. Eng. Chem. Res. 2023
10	Au/TS-1-B(4)	160	144	Chem. Eng. Sci. 2020, 227, 115907
11	Au/TS-1-B	151.5	151.5	ACS Catal. 2023, 13, 2069-2085
12	0.04Au/TS-1(121)Cs	245	98	J. Catal. 2013, 308, 98-113
13	0.05Au/U-TS-1(119)	130	65	J. Catal. 2014, 313, 104-112
14	0.07Au/U-TS-1(119)	127.1	89	J. Catal. 2014, 313, 104-112
15	0.09Au/U-TS-1(119)	106.7	96	J. Catal. 2014, 313, 104-112
16	0.76Au/TS-1(77)Cs	41.3	314	J. Catal. 2013, 308, 98-113
17	0.14Au/TS-1(121)Cs	240.7	337	J. Catal. 2013, 308, 98-113
18	Au/TS-1-B (S-Na)	297	89.1	Appl. Catal. B: Environ. 2022, 319, 121837

Table S4 Nitrogen-adsorption-desorption results of titanosilicalites after different crystallization time.

	[a]S_{BET}/m^2g^{-1}	[b]Micropore volume/cm^3g^{-1}
1h	478.8	0.195
1.5h	465.7	0.194
2h	459.1	0.191
3h	448.9	0.190

[a]The surface area was determined by BET model. [b]Micropore volume was determined by t-Plot method.

Table S5 The content of acid site in titanosilicalites after different crystallization time.

Crystallization time (h)	Acid sites (mmol/g)[a]
1	6.24×10^{-2}
1.5	5.60×10^{-2}
2	5.09×10^{-2}
3	4.63×10^{-2}

[a]The concentration of acid sites was determined by NH_3-TPD.

Table S6 Mass data of synthesizing titanosilicalites using oil heating derived from Aspen Plus V11

	TEOS	WATER	S4	SIOH	ETHAN	COLD1	S13	SIO2
Temperature, ℃	25.00	25.00	25.00	17.65	71.53	20.00	170.00	170.00
Pressure,Mpa	0.10	0.10	0.10	0.10	0.10	0.10	0.10	0.10
Mass flow,kg/h	3048.00	2100.00	7699.81	3412.37	4287.45	3412.37	2551.81	860.55
**Component (wt%）**								
TEOS	1.00		0.00	0.01	0.00	0.01	0.01	0.00
H4SIO			0.44	0.98	0.01	0.59	0.78	0.01
ETHAN			0.35	0.01	0.63	0.01	0.01	0.00
WATER		1.00	0.20	0.00	0.36	0.15	0.20	0.01
SIO2			0.00	0.00	0.00	0.25	0.00	0.98

Table S7 Mass data of synthesizing titanosilicalites using air heating derived from Aspen Plus V11

	TEOS	WATER	S4	SIOH	ETHAN	COLD1	S13	SIO2
Temperature, ℃	25.00	25.00	25.00	17.93	60.42	20.00	170.00	170.00
Pressure,Mpa	1.00	0.10	1.00	1.00	1.00	1.00	1.00	1.00
Mass flow,kg/h	3048.00	2100.00	7971.91	3183.33	4788.57	3183.33	2823.91	359.37
**Component (wt%）**								
TEOS	1.00		0.00	0.01	0.00	0.01	0.01	0.00
H4SIO	0.00		0.50	0.98	0.17	0.81	0.91	0.02
ETHAN	0.00		0.34	0.01	0.56	0.01	0.01	0.00
WATER	0.00	1.00	0.16	0.01	0.26	0.07	0.08	0.01
SIO2	0.00		0.00	0.00	0.00	0.11	0.00	0.97

Table S8 Technical and Economic Performance of Route 1- Production Cost Analysis (Oil 3 Hours)

	Component	Quantity	Unit	Price yuan/kg	Cost: yuan	USD exchange rate	Cost in US dollars/kg
Raw material	Tetraethyl orthosilicate	0.0305	kg	16[a]	0.488	7	0.0697
	distilled water	0.021	kg	0.5[b]	0.0105	7	0.0015
	Tetrabutyl titanate	0.00025	kg	20[c]	0.005	7	0.0007
	Tetrapropyl ammonium hydroxide	0.017	kg	27[d]	0.459	7	0.0656
Public works	power	0.8	kwh	0.5	0.4	7	0.0571
	steam	0.01	kg	0.3	0.003	7	0.0004
	fuel gas	0.35	Nm3	2.5	0.875	7	0.125
Total cost/kg	zeolite	0.0285	kg		2.2405	7	0.3201
Total cost/t	zeolite	1000	kg		78614.03509	7	11230.58

[a] Price source of tetraethyl orthosilicate: Production of mortar and cement using industrial grade CAS562-90-3 tetraethyl orthosilicate polymer chemistry (baidu.com) (accessed on August 22, 2024).
https://b2b.baidu.com/s?q=78-10-4&from=search&fid=67174400%2C1732792976870&pi=b2b.land.search...7746709640418212

[b] Price source of distilled water: Distilled water laboratory uses industrial grade deionized water large barrels for affordable packaging Mingfeng (baidu.com) (accessed on January 26, 2024).
https://b2b.baidu.com/land?url=https%3A%2F%2Fwww.zhaosw.com%2Fproduct%2Fdetail%2F42862270&query=&lattr=&xzhid=30705153&pi=b2b.shop.prodmenu_rec.2.pcsphm.4424623508562891&category=%E6%9C%BA%E6%A2%B0%E8%AE%BE%E5%A4%87%3B%E6%B0%B4%E5%A4%84%E7%90%86%E8%AE%BE%E5%A4%87%3B%E5%87%80%E6%B0%B4%E8%AE%BE%E5%A4%87&fid=67174400%2C1732792976870&iid=ae07be0320af1bfb1f7893d11fd6ba4e&miniId=8469&jid=2354014883&prod_type=0

[c] Price source of tetrabutyl titanate: Spot supply of industrial grade tetrabutyl titanate with a content of 99% catalyst adhesive and high-purity n-butyl titanate (1688. com) (accessed on August 22, 2024).
https://detail.1688.com/offer/740903508796.html

[d] Price source of tetrapropyl ammonium hydroxide: Xingyan content 99% tetrapropylammonium hydroxide organic synthesis 4499-86-9 (baidu. com) (accessed on January 26, 2024).
https://detail.1688.com/offer/642230586184.html?spm=a26352.13672862.offerlist.173.54fb1e626ropMJ

Table S9 Technical and Economic Performance of Route 2- Production Cost Analysis (Air 20 Hours)

	Component	Quantity	Unit	Price yuan/kg	Cost: yuan	USD exchange rate	Cost in US dollars/kg
Raw material	Tetraethyl orthosilicate	0.0305	kg	16[a]	0.488	7	0.0697
	distilled water	0.021	kg	0.5[b]	0.0105	7	0.0015
	Tetrabutyl titanate	0.00025	kg	20[c]	0.005	7	0.0007
	Tetrapropyl ammonium hydroxide	0.017	kg	27[d]	0.459	7	0.0656
Public works	power	2.52	kwh	0.5	1.26	7	0.18
	steam	3.792	kg	0.3	1.1376	7	0.1625
	Fuel gas	0.35	Nm3	2.5	0.875	7	0.125
Total cost/kg	zeolite	0.04275	kg		4.2351	7	0.6050
Total cost/t	zeolite	1000	kg		99066.67	7	14152.38

[a] Price source of tetraethyl orthosilicate: Production of mortar and cement using industrial grade CAS562-90-3 tetraethyl orthosilicate polymer chemistry (baidu.com) (accessed on August 22, 2024).
https://b2b.baidu.com/s?q=78-10-4&from=search&fid=67174400%2C1732792976870&pi=b2b.land.search...7746709640418212

[b] Price source of distilled water: Distilled water laboratory uses industrial grade deionized water large barrels for affordable packaging Mingfeng (baidu.com) (accessed on January 26, 2024).
https://b2b.baidu.com/land?url=https%3A%2F%2Fwww.zhaosw.com%2Fproduct%2Fdetail%2F42862270&query=&lattr=&xzhid=30705153&pi=b2b.shop.prodmenu_rec.2.pcsphm.4424623508562891&category=%E6%9C%BA%E6%A2%B0%E8%AE%BE%E5%A4%87%3B%E6%B0%B4%E5%A4%84%E7%90%86%E8%AE%BE%E5%A4%87%3B%E5%87%80%E6%B0%B4%E8%AE%BE%E5%A4%87&fid=67174400%2C1732792976870&iid=ae07be0320af1bfb1f7893d11fd6ba4e&miniId=8469&jid=2354014883&prod_type=0

[c] Price source of tetrabutyl titanate: Spot supply of industrial grade tetrabutyl titanate with a content of 99% catalyst adhesive and high-purity n-butyl titanate (1688. com) (accessed on August 22, 2024).
https://detail.1688.com/offer/740903508796.html

[d] Price source of tetrapropyl ammonium hydroxide: Xingyan content 99% tetrapropylammonium hydroxide organic synthesis 4499-86-9 (baidu. com) (accessed on January 26, 2024).
https://detail.1688.com/offer/642230586184.html?spm=a26352.13672862.offerlist.173.54fb1e626ropMJ

The cost analysis reveals that Route 1 (liquid phase) consumes 0.8 kWh of electricity, 0.01 kg of public steam, and 0.35 Nm^3 of combustion gas, resulting in a final mass of titanosilicates of 0.0285 kg. In contrast, Route 2 (gas phase) utilizes 2.52 kWh of electricity, along with 3.792 kg of steam and 0.35 Nm^3 of fuel gas, yielding 0.04275 kg of titanosilicates. The cost breakdown, considering market prices of raw materials and utilities (Table S8 and S9), shows that Route 1 has a higher proportion of raw material costs, particularly associated with TEOS and TPAOH, with fuel gas accounting for 39.05% of total costs. In contrast, Route 2 has a lower raw material cost proportion (< 12%) but higher utility costs, especially electricity (29.75%) due to the extended operation time needed to reach similar TS-1 crystallinity. In summary, producing 0.0285 kg of titanosilicalite in Route 1 costs 0.31 USD, while Route 2 costs 0.59 USD for 0.04275 kg of titanosilicalite. Extrapolated, Route 1 costs 10,980.84 USD per ton, and Route 2 costs 13,837.67 USD.

4. Supplementary Figures

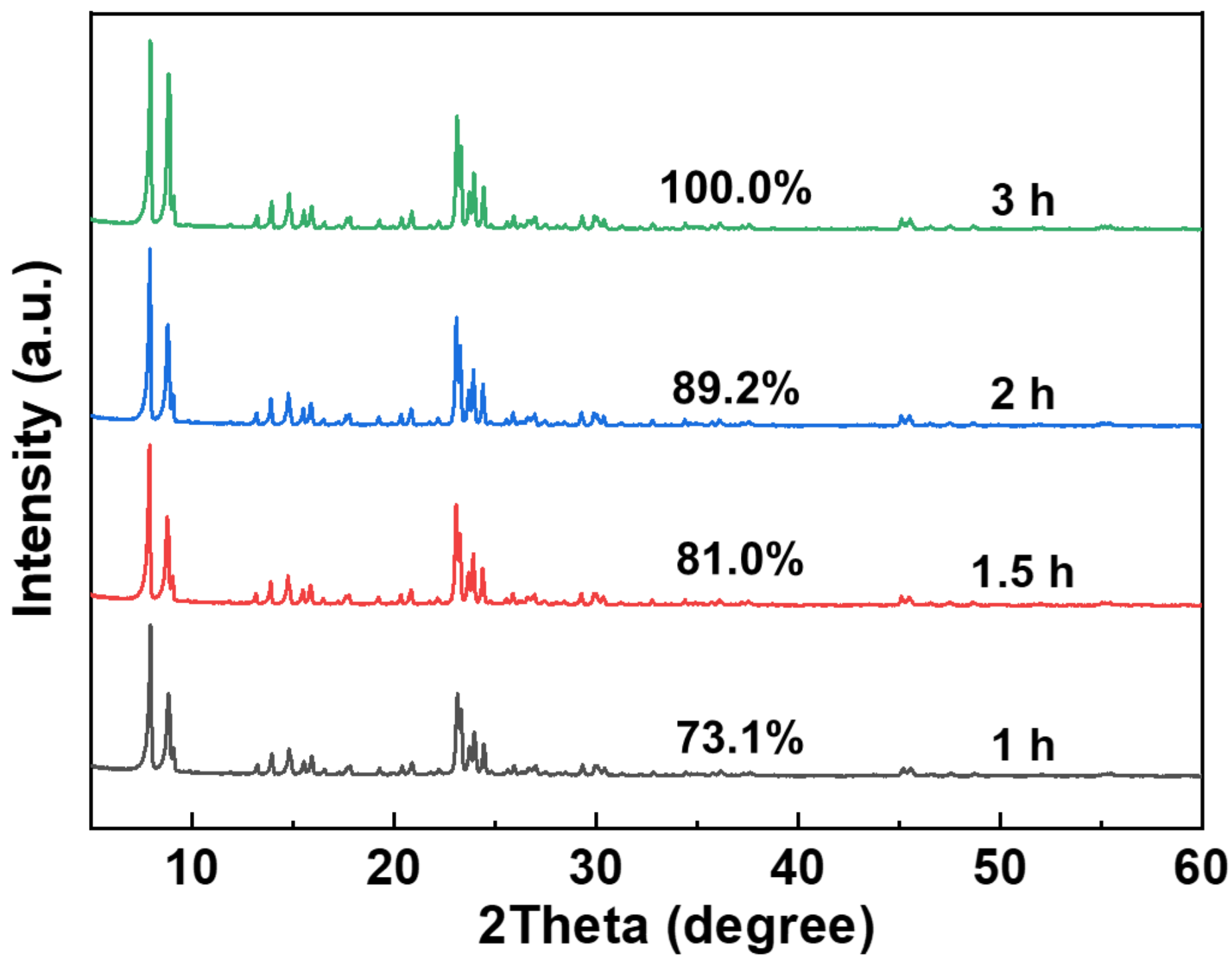

Figure S1 The XRD patterns of titanosilicalites crystallized in the oil (L-TS-1).

All samples show five typical diffraction peaks at 7.9°, 8.8°, 23.1°, 23.3°, and 24.4°which could be attributed to the MFI topological structure.

For the synthesis of titanosilicalite-1 (TS-1), 30.48 g of tetraethyl orthosilicate, 17.00 g of tetrapropylammonium hydroxide (25 wt%), 0.25 g of tetrabutyl titanate, and 21 g of deionized water were mixed directly and under irradiation by ultraviolet for 1h. The obtained sol gel was crystallized at 170 ^{o}C with a specific time. The sol gel was heated by dimethicone oil in a stainless-steel tube with an external diameter of 12 mm, a thickness of 1.5 mm, and a length of 10 cm. After crystallization, the as-synthesized TS-1 after centrifugation and washing was dried at 80 ^{o}C for 8 h and calcinated at 550 ^{o}C for 6 h. The obtained white powders heated by dimethicone oil were referred as L-TS-1.

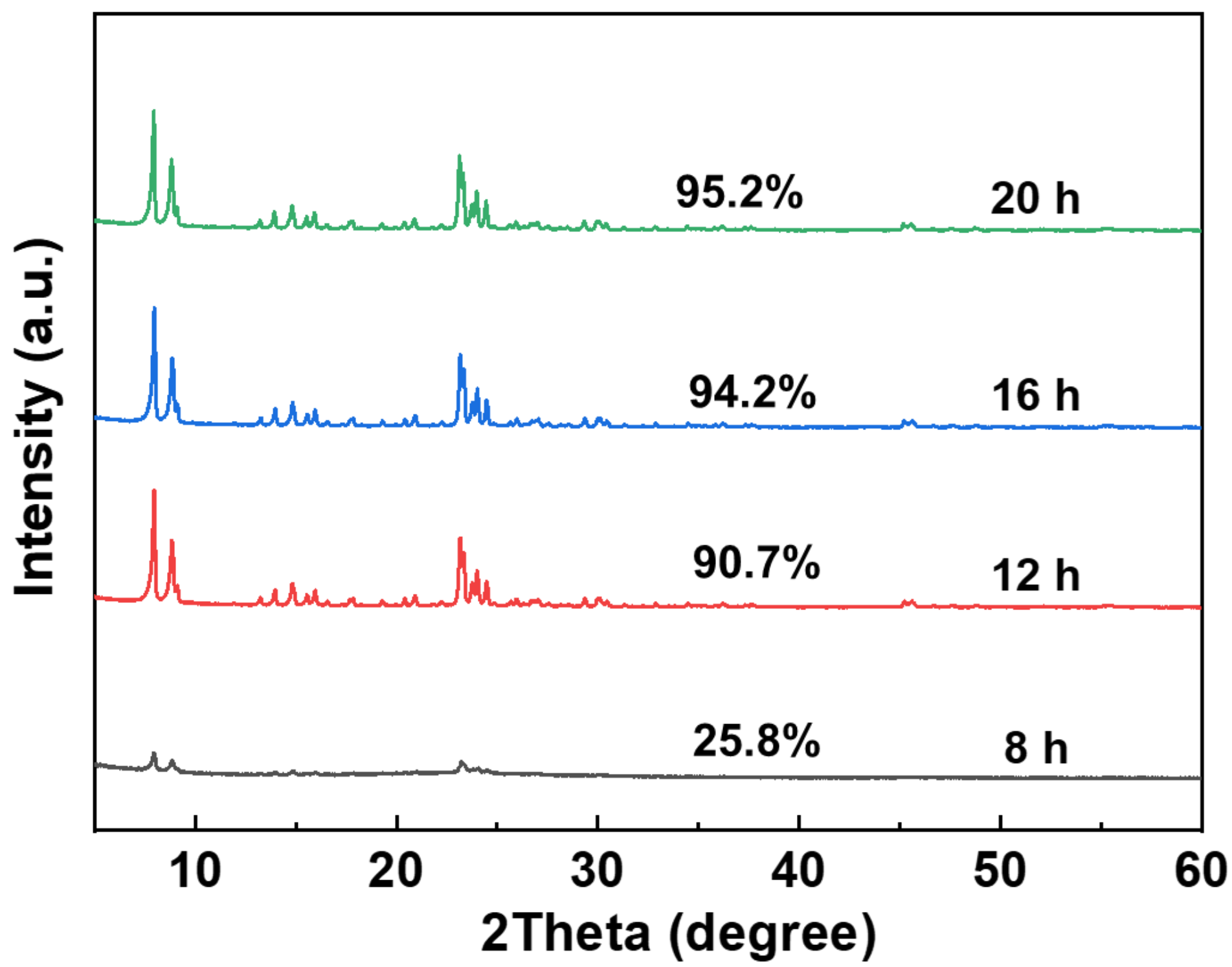

Figure S2 The XRD patterns of titanosilicalites crystallized in the air (G-TS-1).

For the synthesis of titanosilicalite-1 (TS-1), 30.48 g of tetraethyl orthosilicate, 17.00 g of tetrapropylammonium hydroxide (25 wt%), 0.25 g of tetrabutyl titanate, and 21 g of deionized water were mixed directly and under irradiation by ultraviolet for 1h. The obtained sol gel was crystallized at 170 ^{o}C with a specific time. The sol gel was heated by air in a stainless-steel tube with an external diameter of 12 mm, a thickness of 1.5 mm, and a length of 10 cm. After crystallization, the as-synthesized TS-1 after centrifugation and washing was dried at 80 ^{o}C for 8 h and calcinated at 550 ^{o}C for 6 h. The obtained white powders heated by air were referred as G-TS-1.

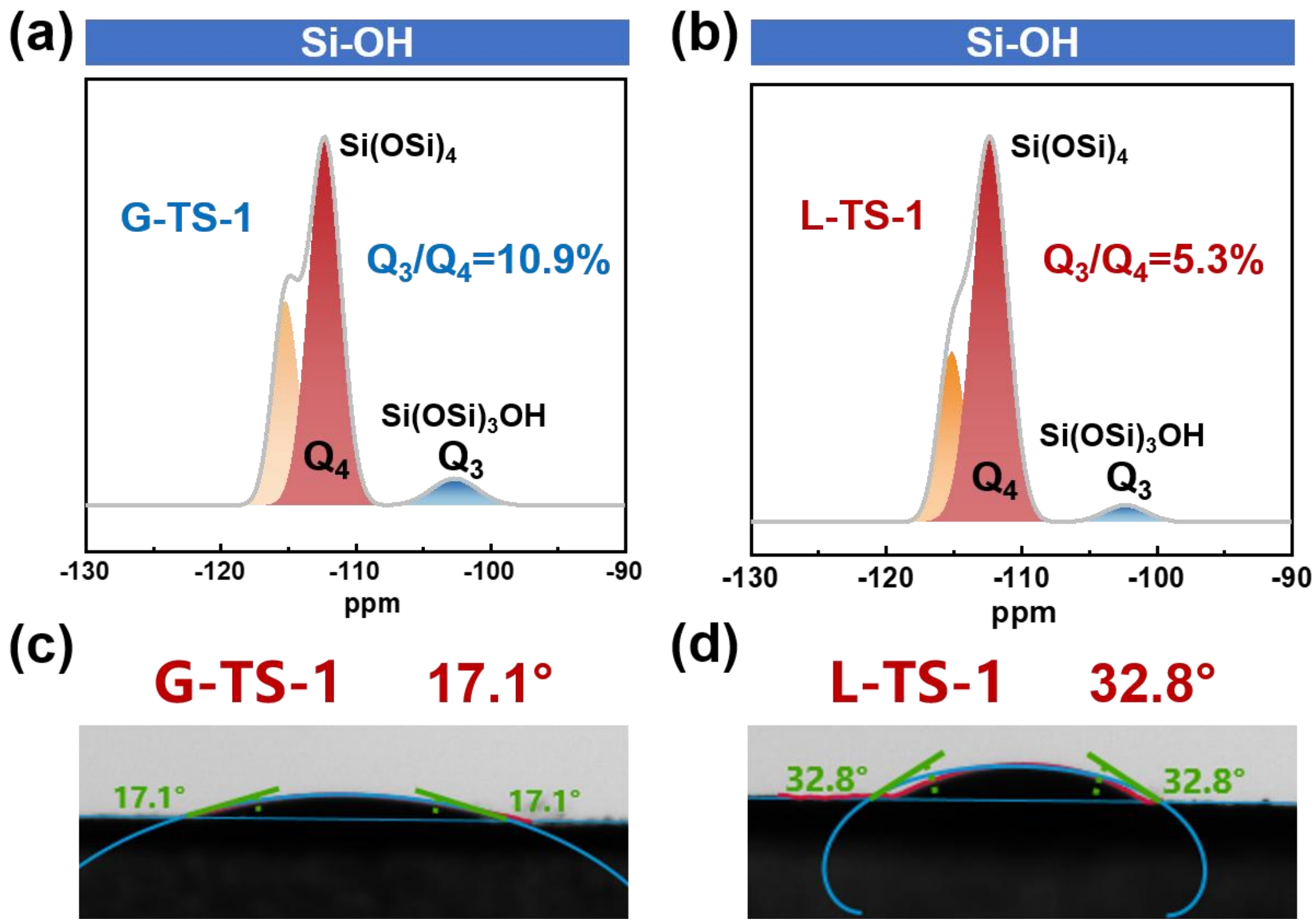

Figure S3 The ^{29}Si MAS NMR spectra of (a) G-TS-1 and (b) L-TS-1 titanosilicalites. The water-droplet contact angles of (c) G-TS-1 and (d) L-TS-1 titanosilicalites.

Water contact-angle measurements further distinguish the surface properties of the two samples, with L-TS-1 showing a larger contact angle than G-TS-1 (32.8° vs. 17.1°). The increased water contact angle of L-TS-1 is in good agreement with the lower abundance of hydroxyl-related defects identified by 2D ^{1}H-^{1}H DQ-SQ MAS NMR, ^{29}Si MAS NMR, ^{1}H MAS NMR, NH_3-TPD, and Py-IR. These observations support that defect removal creates a more hydrophobic local environment, in good agreement with the experimental results showing weakened retention of oxygenated species, enhanced stabilization of Ti-OOH intermediates, and facilitated PO desorption.

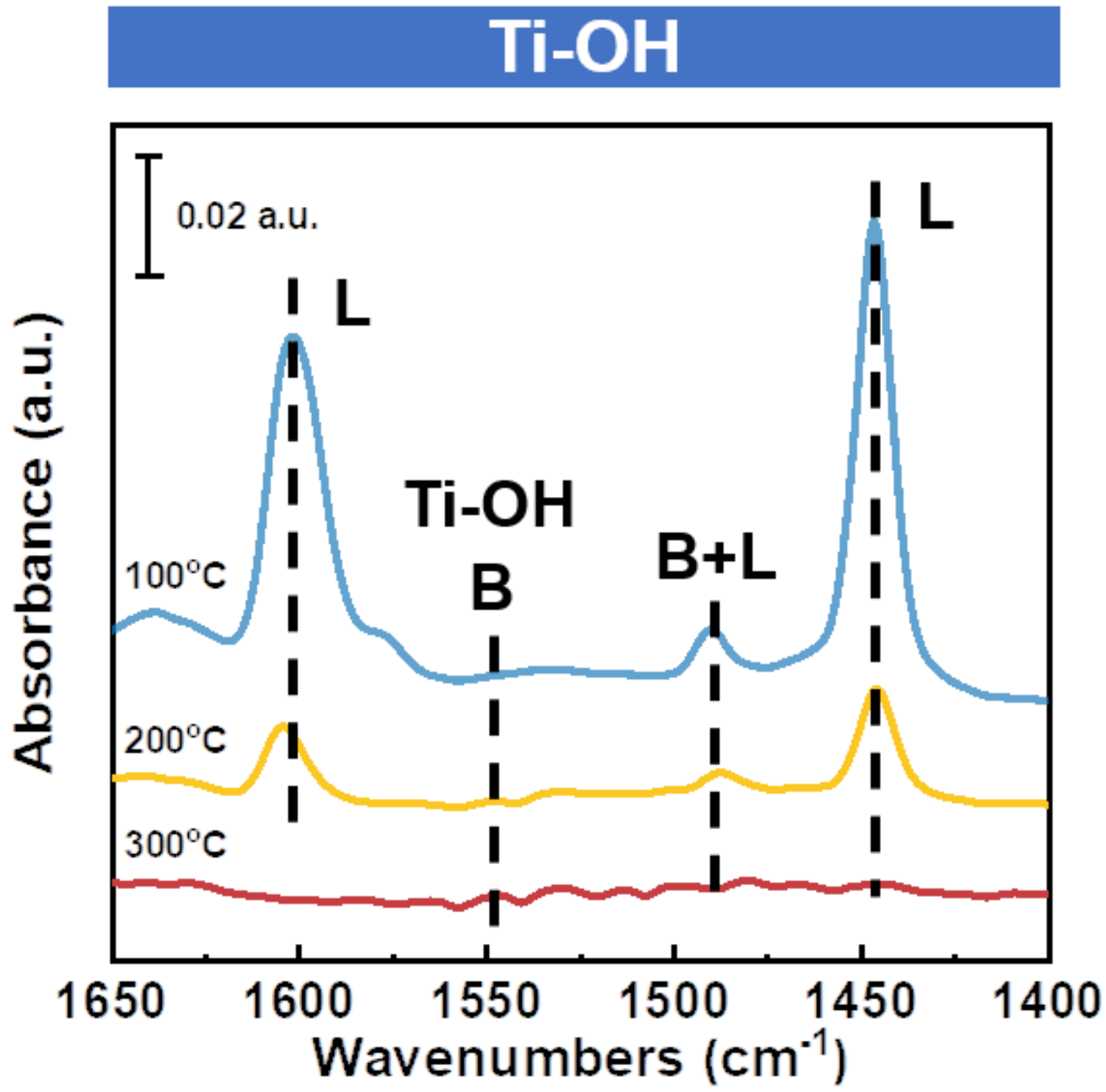

Figure S4 The Py-IR characterization of L-TS-1.

The peaks at 1447 and 1601 cm^{-1} are attributed to Lewis acid sites (LA). Moreover, the peaks at 1550 and 1490 cm^{-1} are ascribed to Brønsted acid sites (BA) and Brønsted together with Lewis acid sites (BA+LA), respectively. In the Figure S4, the intensity of Brønsted and Lewis acid sites gradually decreased with the increase of temperature, and the pyridine was desorbed completely from titanosilicalites at the high temperature of 300 ^{o}C.

The tetrahedrally coordinated Ti species in TS-1 arise from both open and closed Ti sites. Closed Ti sites correspond to $Ti(OSi)_4$ species, while open Ti sites are associated with $Ti(OSi)_3OH$ species. The Brønsted acid sites in TS-1 are linked to the $Ti(OSi)_3OH$ species, which are also classified as tetrahedrally coordinated Ti species.

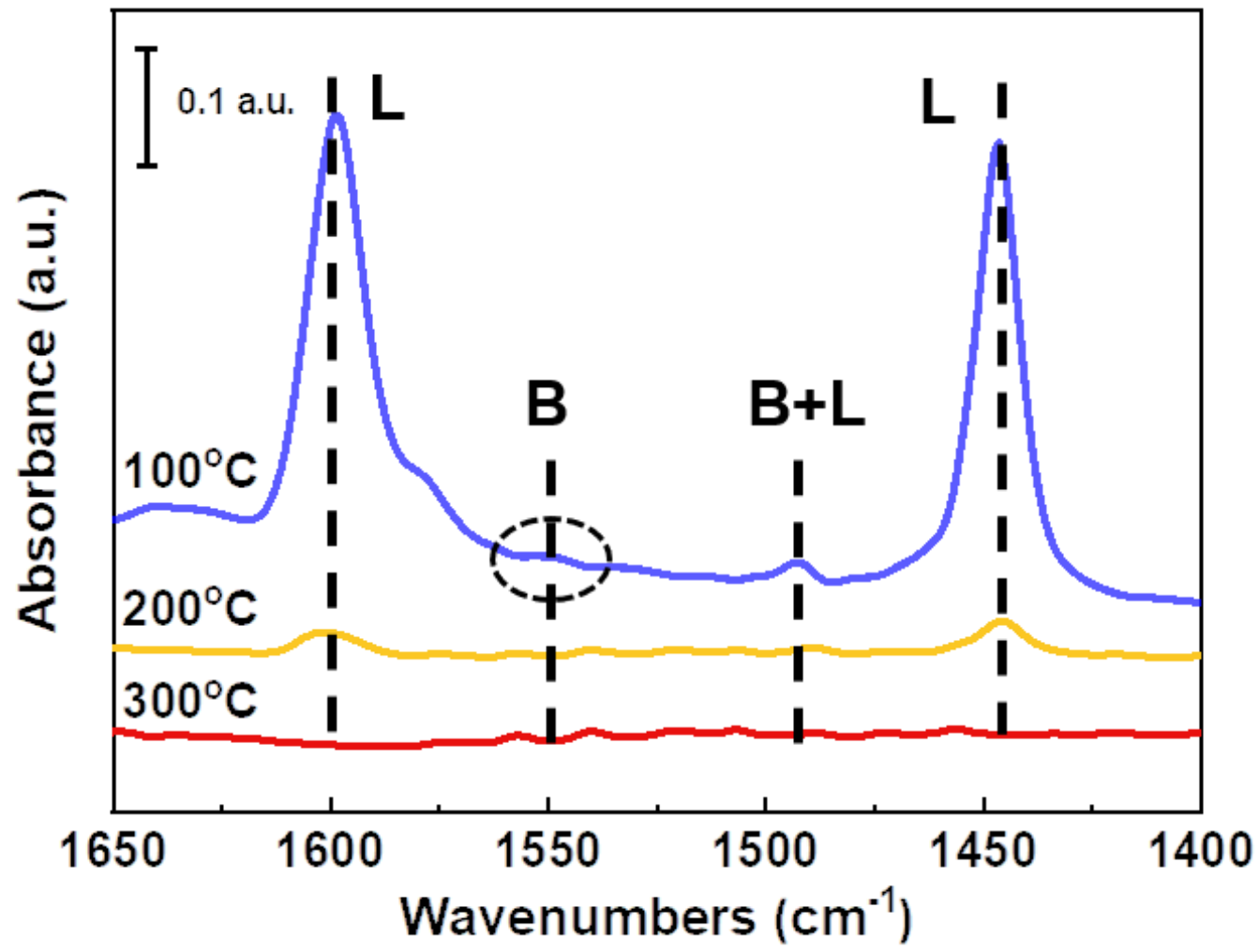

Figure S5 The Py-IR characterization of G-TS-1.

The peaks at 1447 and 1601 cm^{-1} are attributed to Lewis acid sites (LA). Moreover, the peaks at 1550 and 1490 cm^{-1} are ascribed to Brønsted acid sites (BA) and Brønsted together with Lewis acid sites (BA+LA), respectively. In the Figure S5, the intensity of Brønsted and Lewis acid sites gradually decreased with the increase of temperature, and the pyridine was desorbed completely from titanosilicalites at the high temperature of 300 °C. The G-TS-1 showed a weak Brønsted acid peak at 1550 cm^{-1} which could be attributed to small amounts of Ti-OH species.

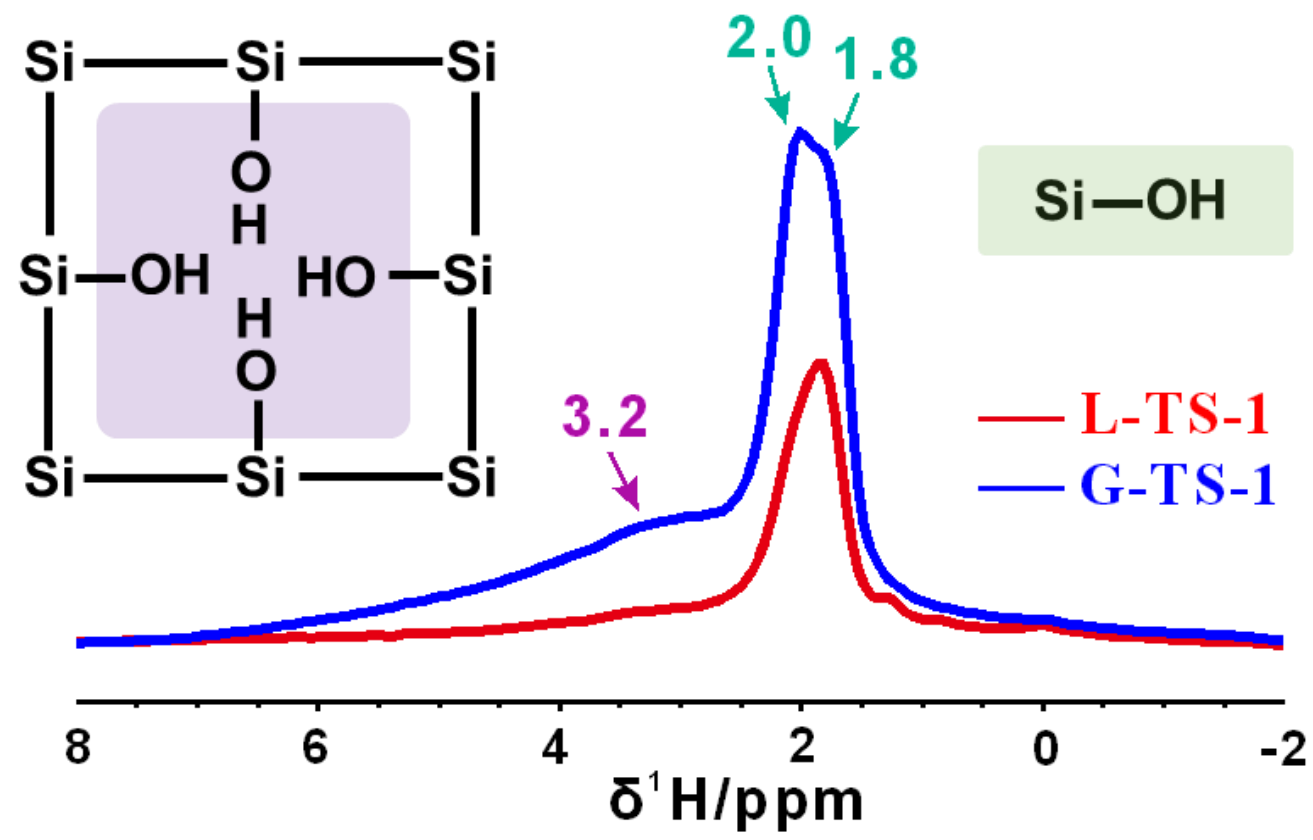

Figure S6 The ^{1}H MAS NMR spectra of L-TS-1 and G-TS-1 titanosilicalites.

To quantitatively probe acid-related defect sites at the atomic level, ^{1}H MAS NMR was employed. In the L-TS-1 sample, signals at 1.8 and 2.0 ppm were attributed to terminal hydroxyl groups. Notably, their concentrations were lower than those observed in the G-TS-1. Additionally, the signal at 3.2 ppm, corresponding to hydrogen-bonded silanols, was also markedly diminished in L-TS-1, indicating effective suppression of these defect species.

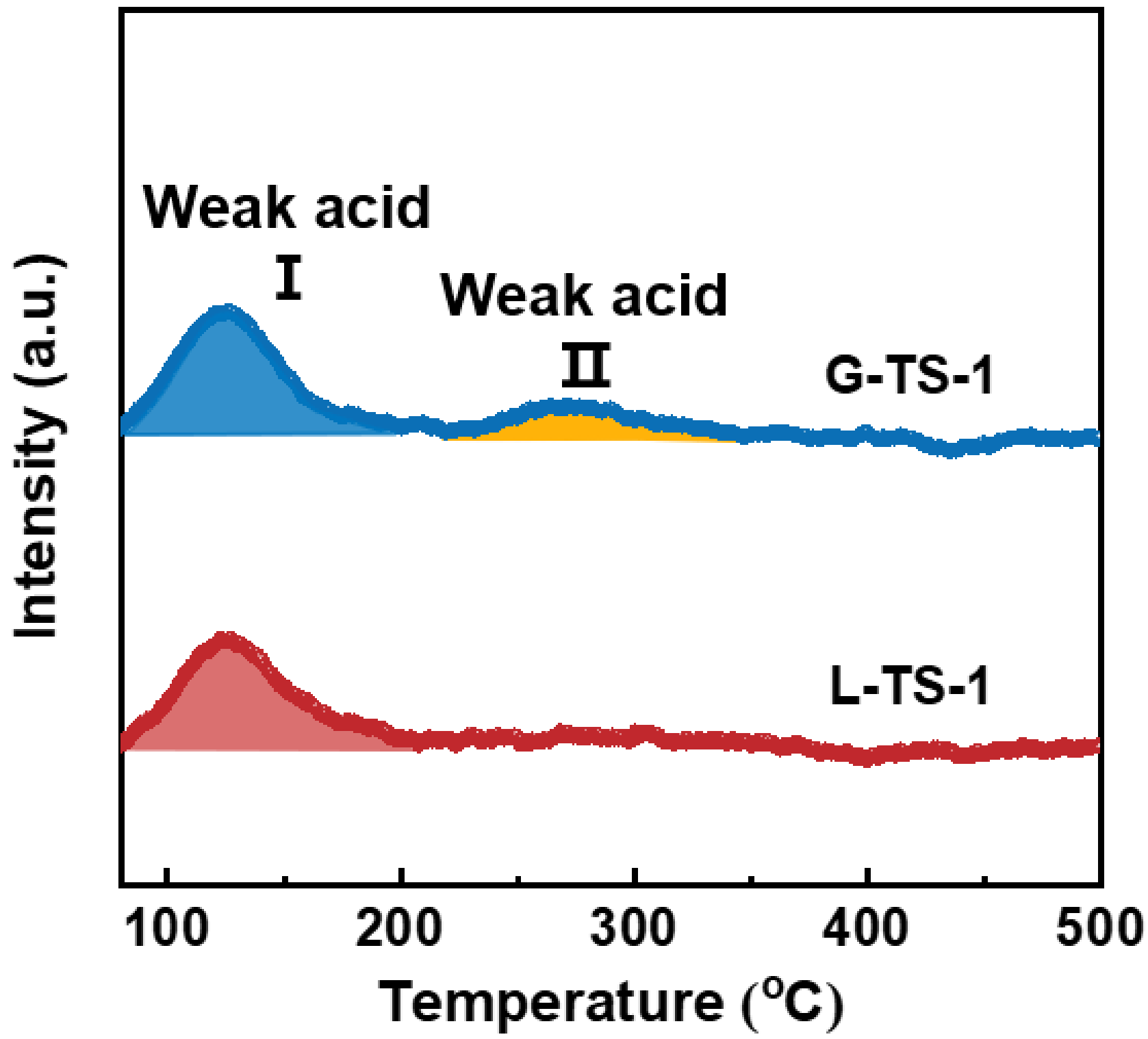

Figure S7 The NH_3-TPD of L-TS-1 and G-TS-1 titanosilicalites.

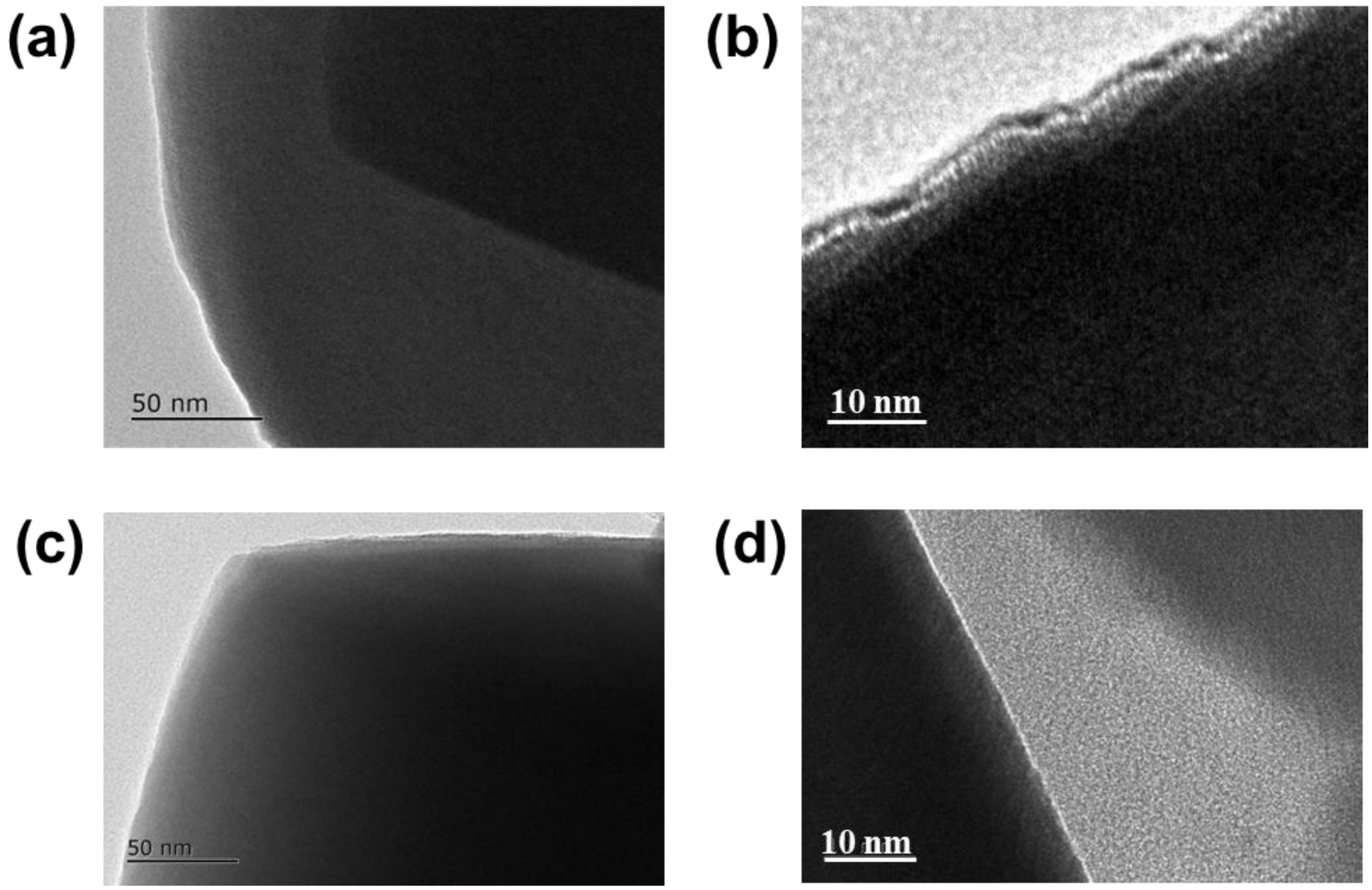

Figure S8 The typical HRTEM images of (a) (b) G-TS-1 and (c) (d) L-TS-1.

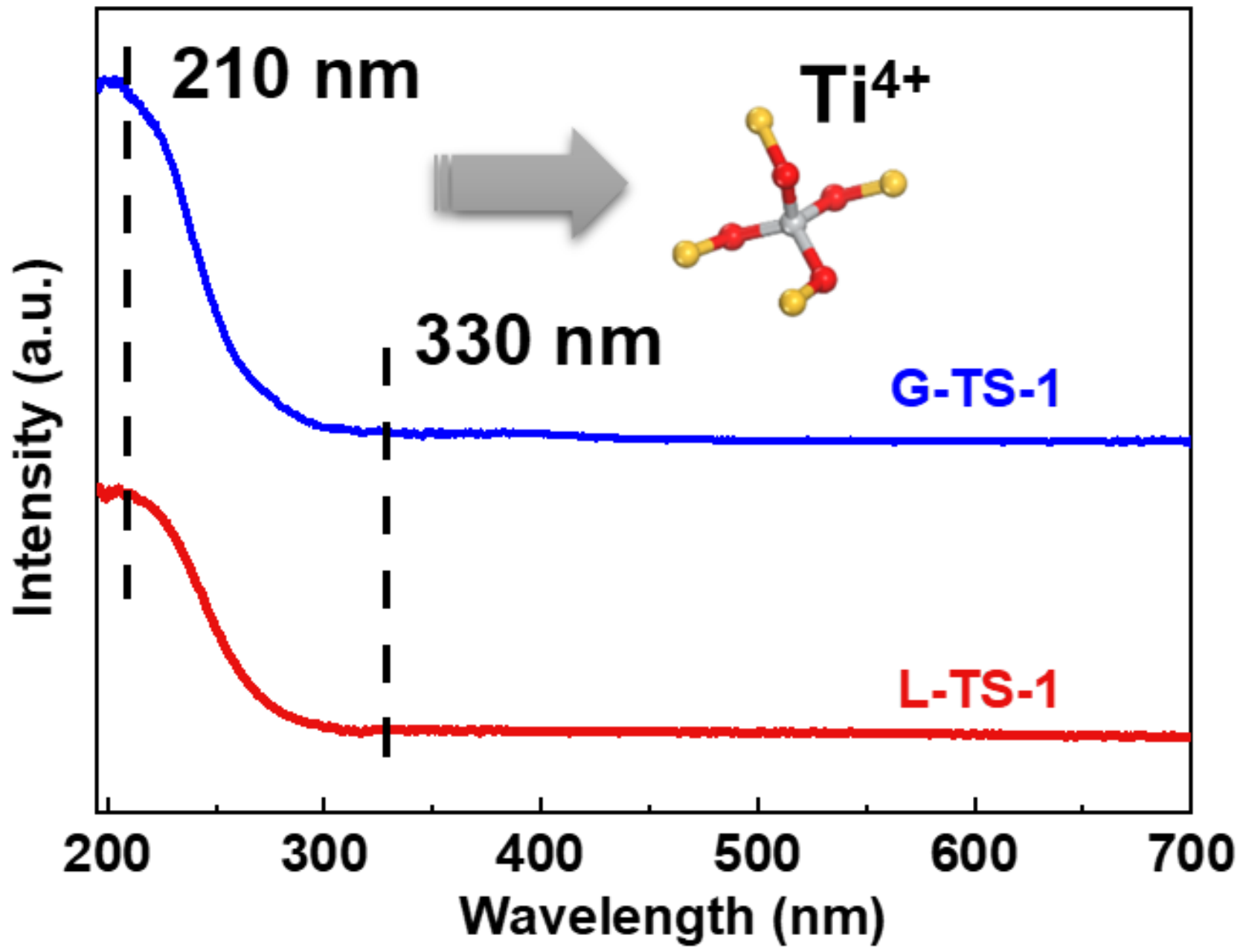

Figure S9 The UV-vis spectra of different titanosilicalites.

The UV-vis spectra showed that the G-TS-1 and L-TS-1 both have tetrahedrally coordinated (TiO_4) Ti species in the framework (~210 nm) without extra-framework anatase species (~330 nm).

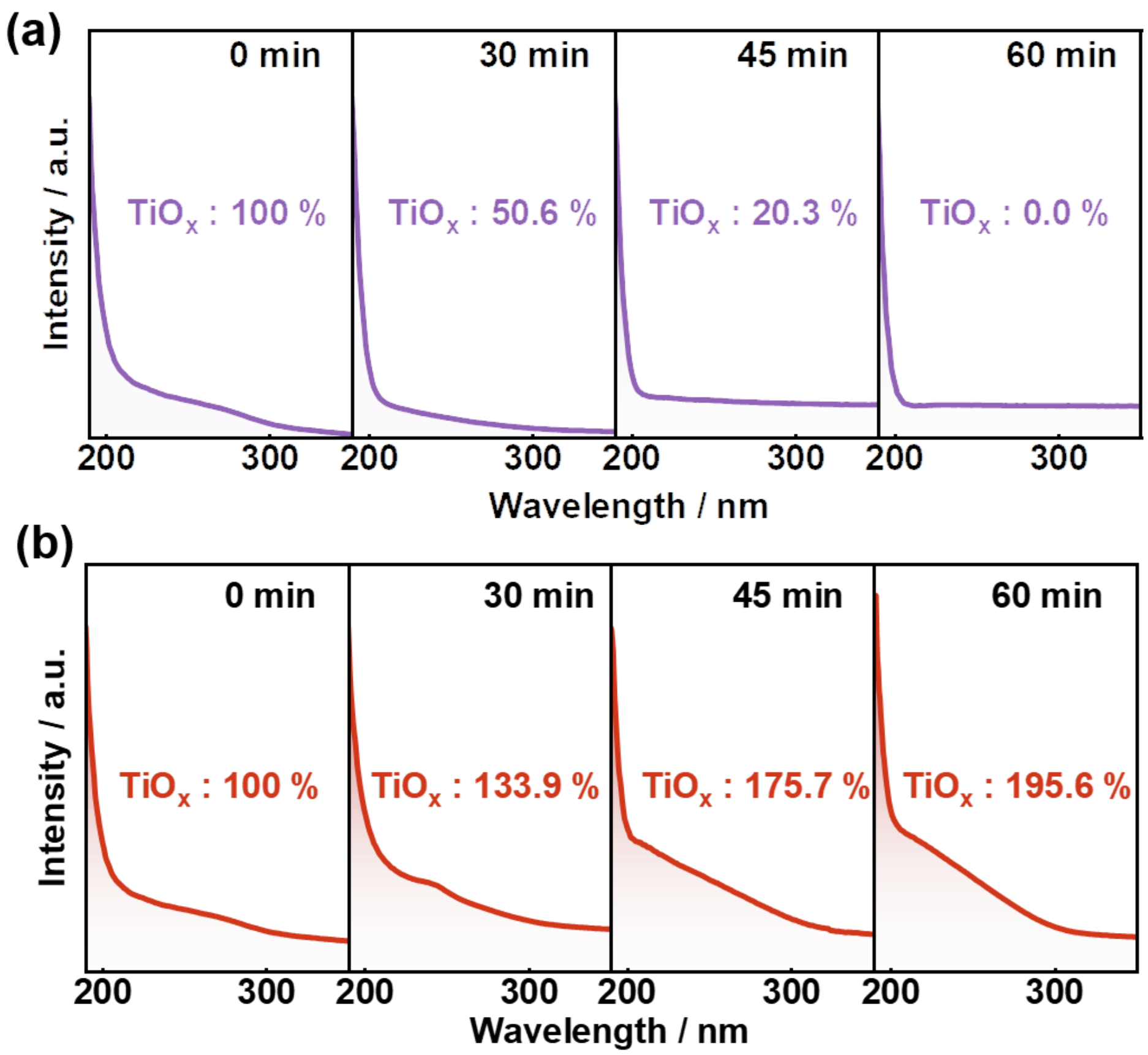

Figure S10 UV-vis spectra of Ti species under (a) UV irradiation and (b) spontaneous hydrolysis conditions.

During the sol-gel synthesis, the dynamic evolution of Ti species in solution under UV irradiation was monitored by UV-vis spectroscopy. When the mixture of Ti and Si precursors were subjected to UV irradiation, the relative fraction of Ti oligomers (band area between 200-400 nm) gradually decreased to 0.0% within 60 min (Figure S10a), preventing polymerization and the formation of extra-framework TiO_2. This behavior is attributed to the deoligomerization of Ti oligomers into corresponding monomers, facilitated by hydroxyl radicals generated under UV irradiation. In contrast, under spontaneous hydrolysis conditions, the relative fraction of Ti oligomers continuously increased from 100.0% to 195.6% over 60 min (Figure S10b), which subsequently promoted further polymerization and the formation of extra-framework TiO_2.

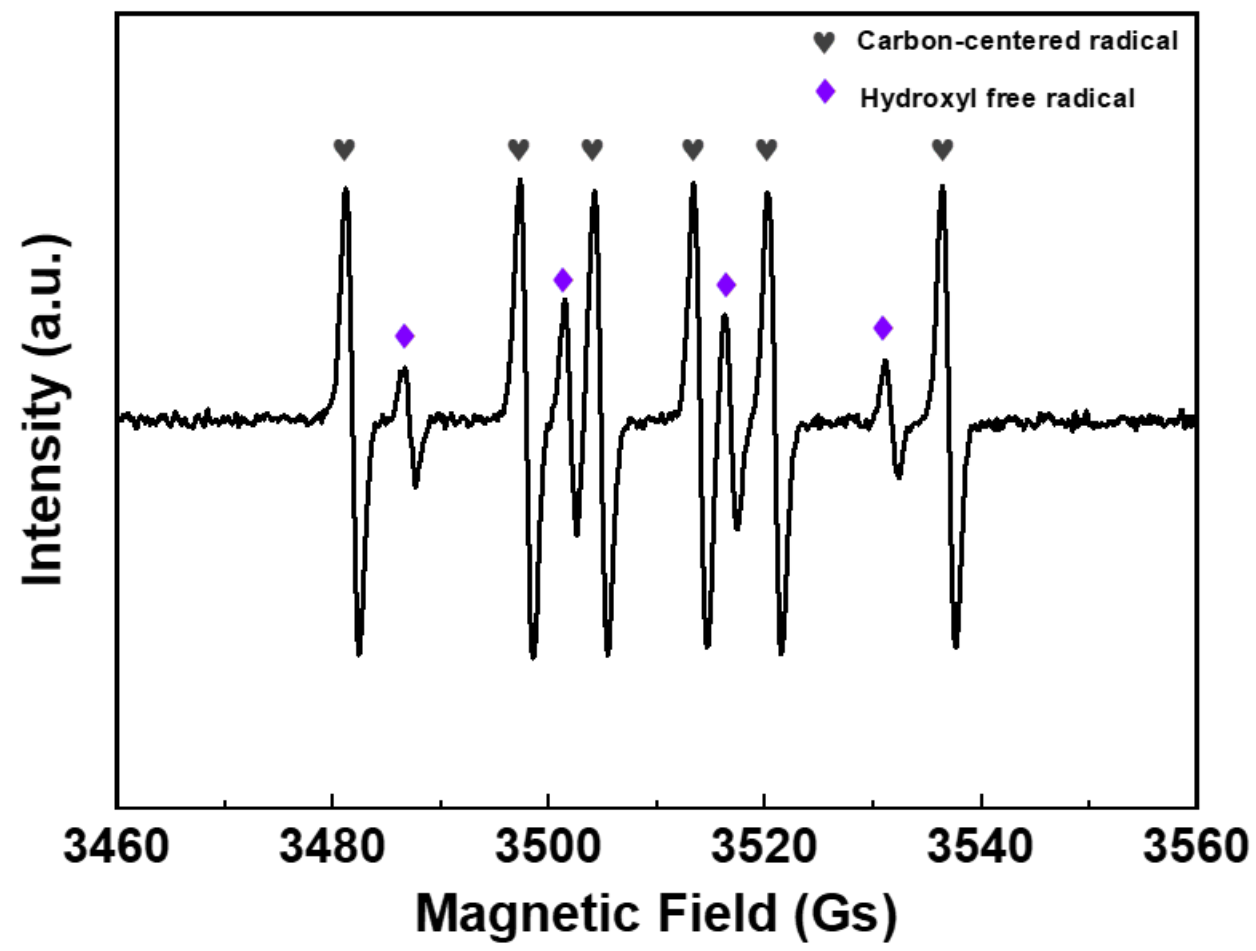

Figure S11 EPR spectra of titanosilicalite synthesis gel containing the spin-trapping agent DMPO under UV irradiation.

The DMPO-hydroxyl free radicals adduct showed a typical 1:2:2:1 quartet pattern with characterized hyperfine coupling constants ($a_N = a_{H\beta} = 15.0$ Gs). The EPR signals with $a_N = 16.0$ Gs and $a_{H\beta} = 22.0$ Gs were attributed to DMPO-carbon-centered radicals adducts, and the generation of carbon-centered radicals was ascribed to the interaction between ·OH and the silicon source (TEOS) during the dissociation process.

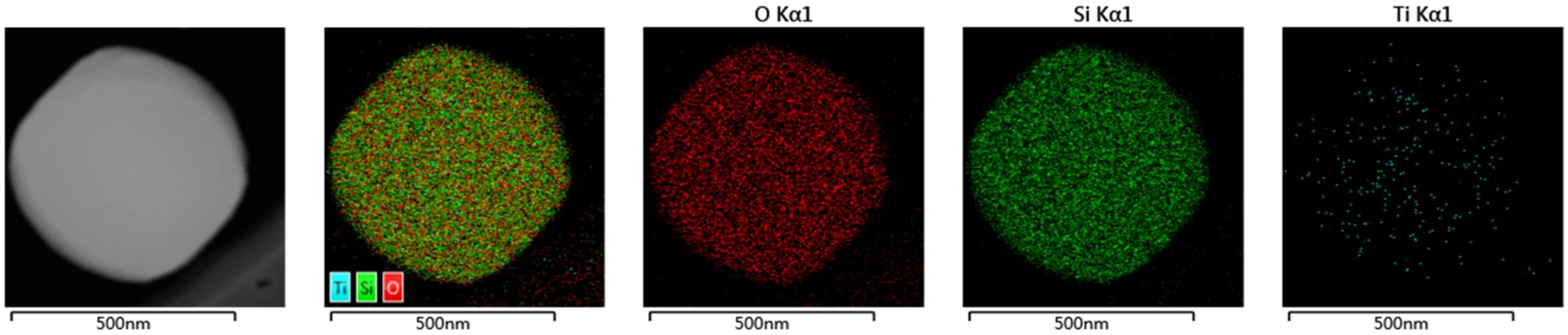

Figure S12 The HAADF-STEM mapping of G-TS-1.

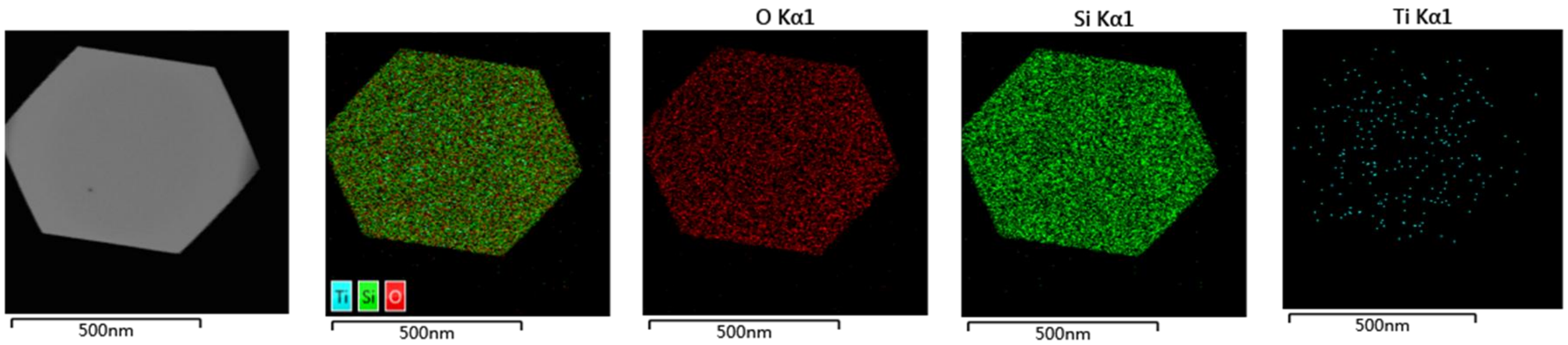

Figure S13 The HAADF-STEM mapping of L-TS-1.

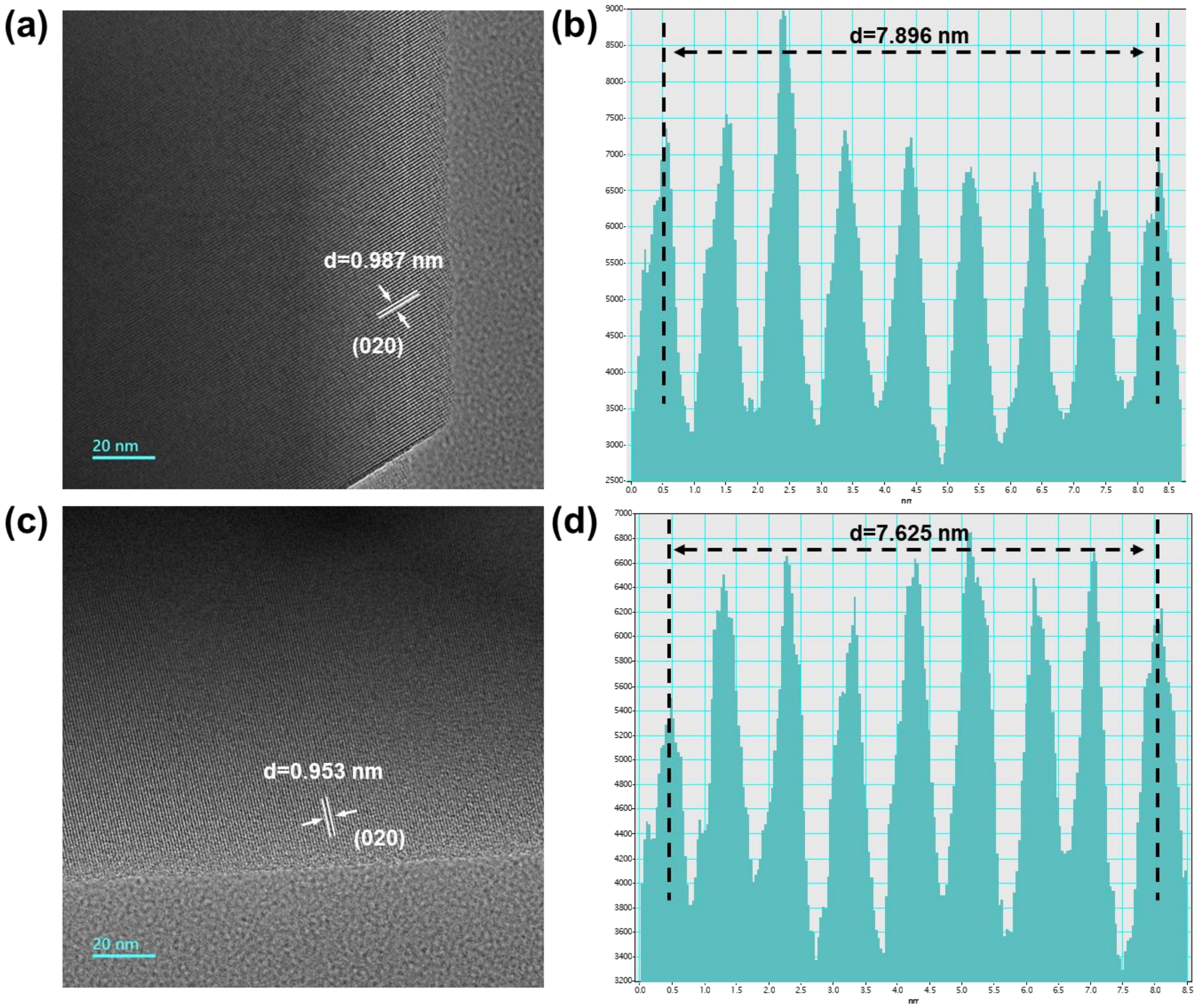

Figure S14 Typical HRTEM images of (a) L-TS-1 and (c) G-TS-1. Line intensity profiles corresponding to (a) and (c) are shown in (b) and (d), respectively.

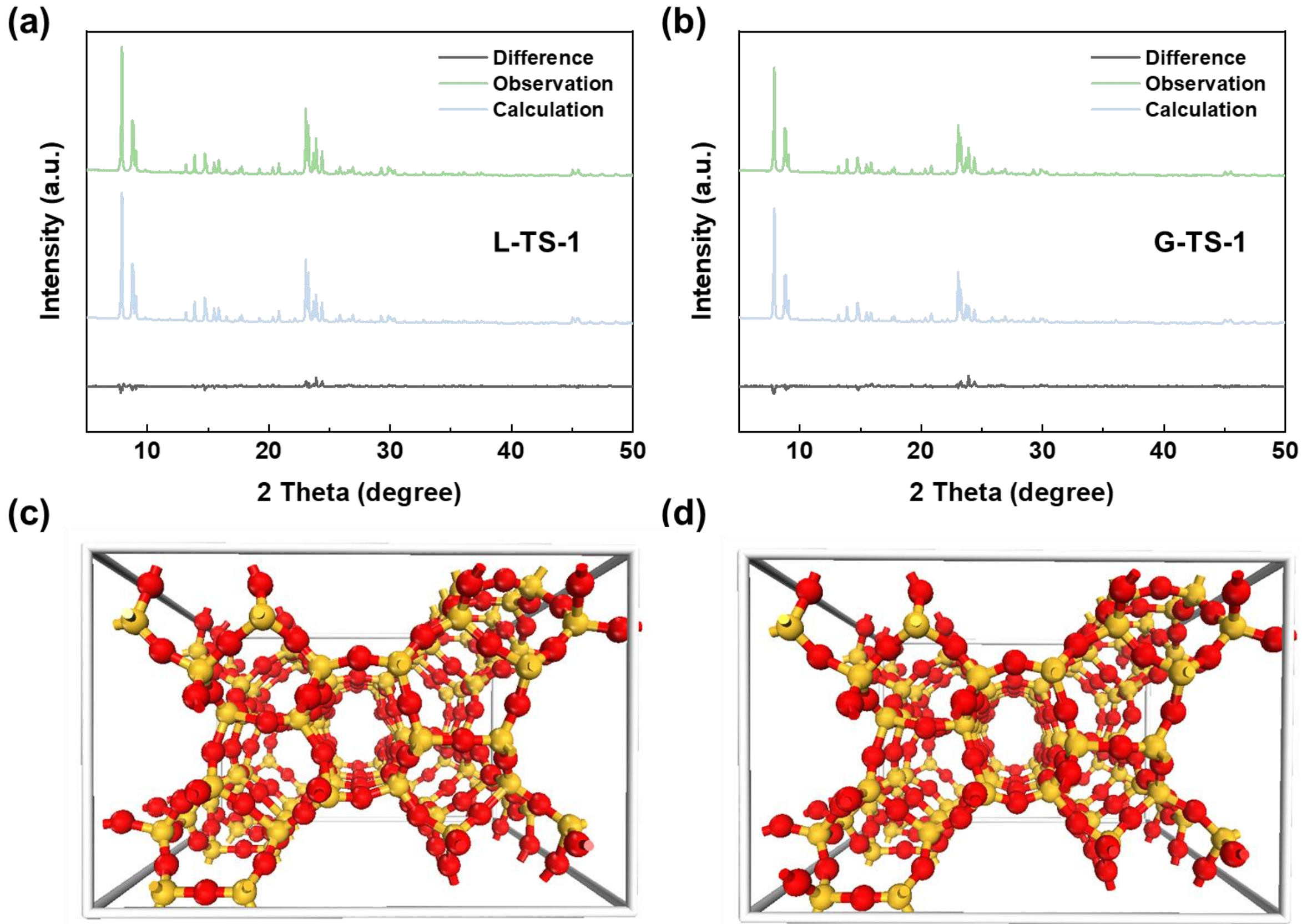

Figure S15 Experimental and calculated XRD patterns of (a) L-TS-1 and (b) G-TS-1 after crystallization at 170 ºC. The unit cell of the (c) L-TS-1 and (d) G-TS-1 calculated by the Rietveld refinement.

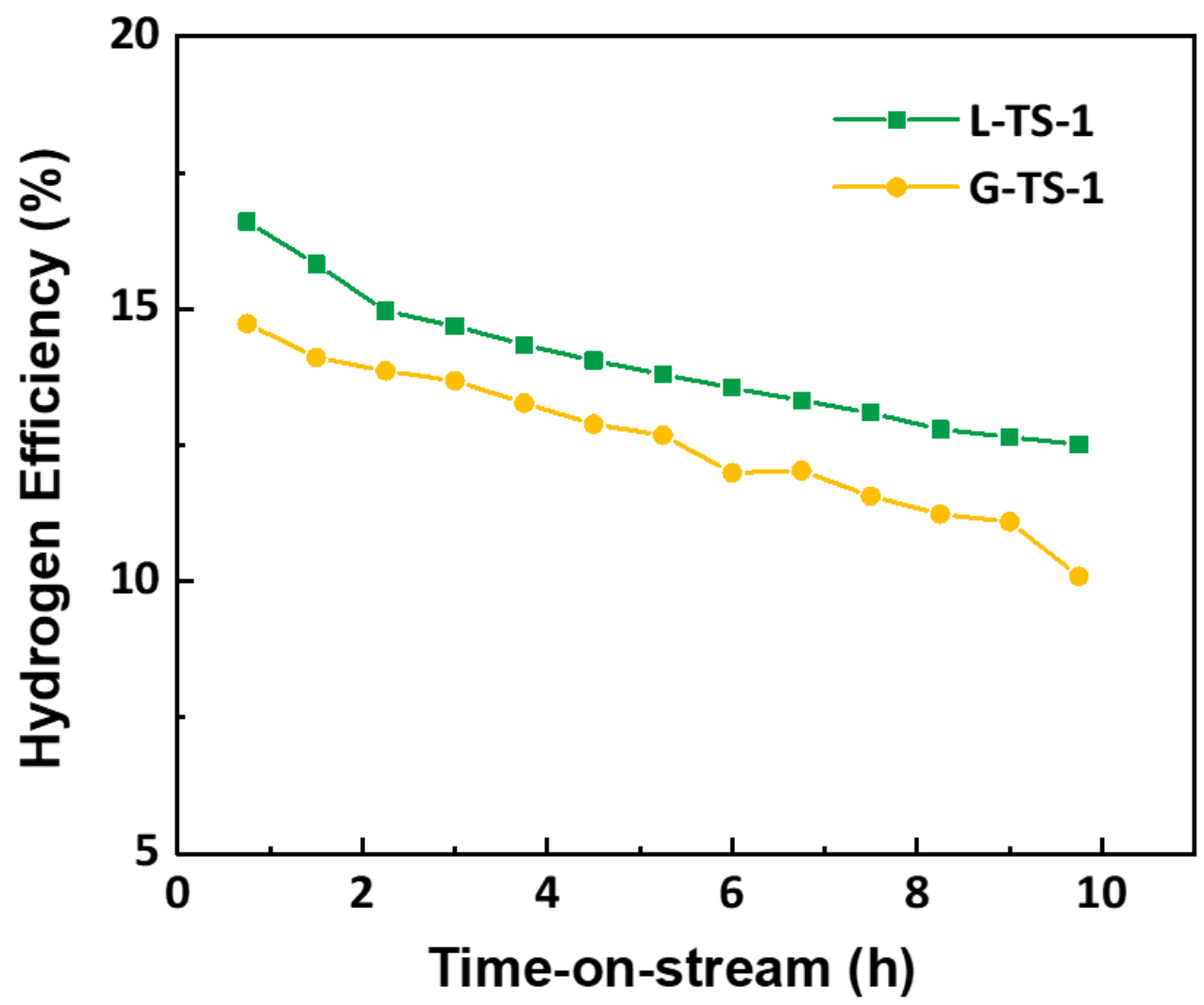

Figure S16 The hydrogen efficiency of different titanosilicalites in the propene epoxidation with H_2 and O_2.

The Au/L-TS-1 and Au/G-TS-1 catalysts were tested in the propene epoxidation with H_2 and O_2 at 200 ºC with a space velocity of 14000 mL•h^{-1}•g$_{Cat}^{-1}$. The reactants contain propene (3.5 mL/min), oxygen (3.5 mL/min), hydrogen (3.5 mL/min), and nitrogen (24.5 mL/min). 0.15 g of catalysts were loaded into the silica tube and further heated from room temperature to 200 ºC with a rate of 1 ºC/min under the atmosphere of feed gas.

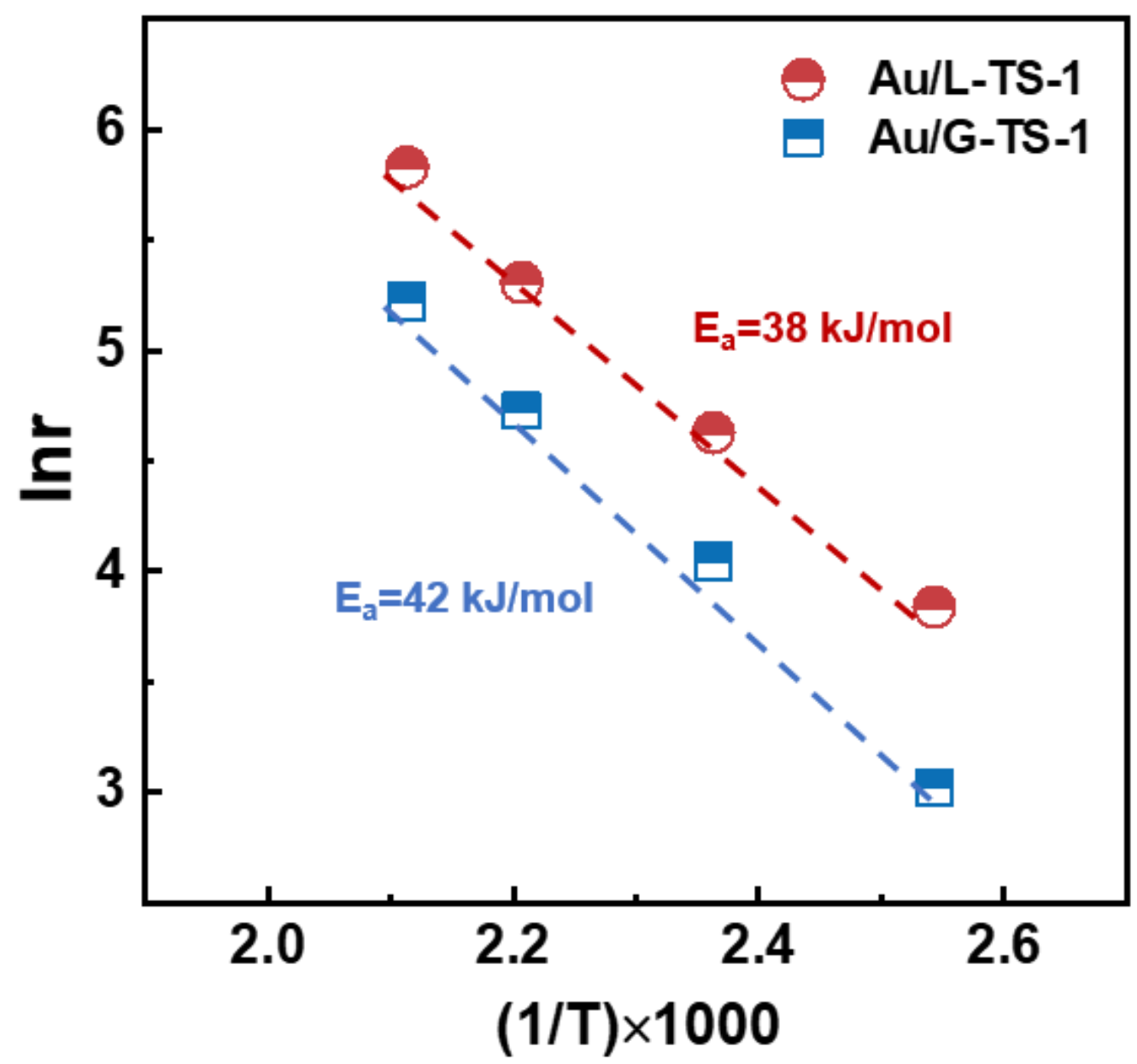

Figure S17 Comparison of activation energies for Au/L-TS-1 and Au/G-TS-1.

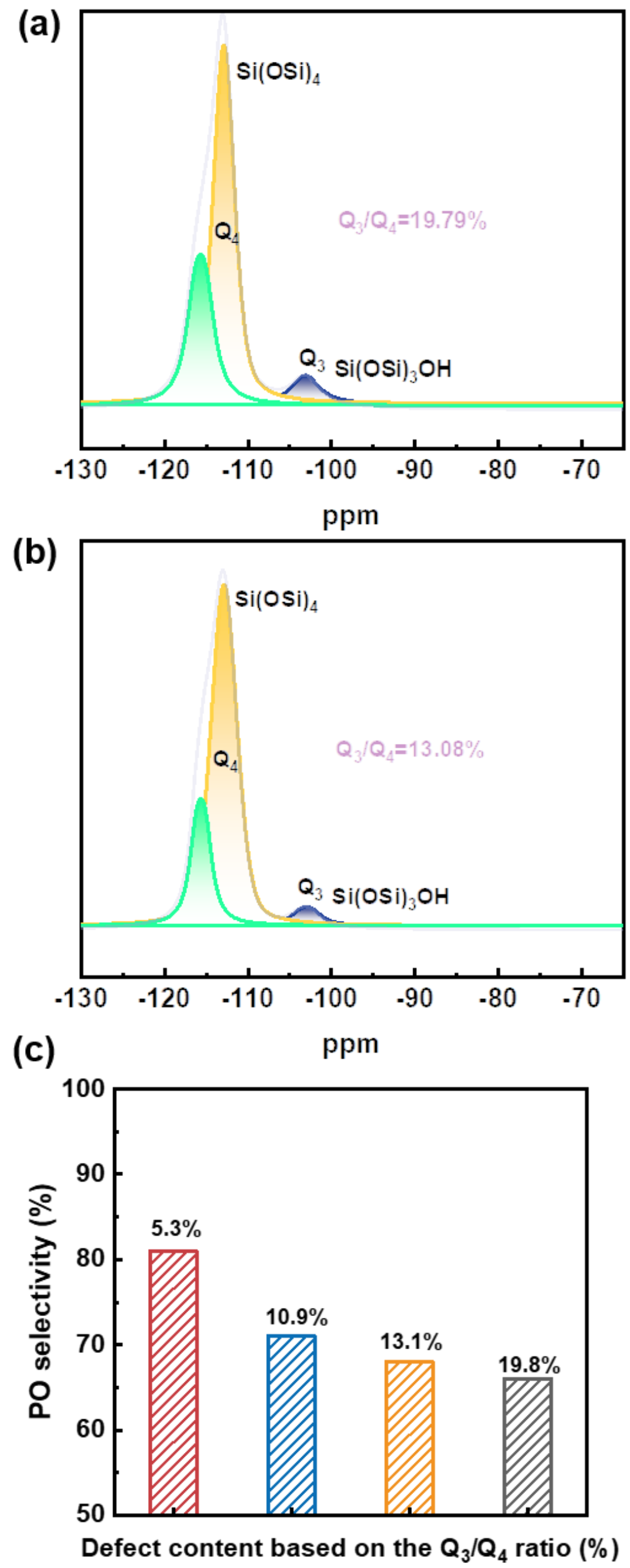

Figure S18 The ^{29}Si MAS NMR spectra of TS-1 synthesized in air after crystallization for (a) 24 h and (b) 36 h. (c) Relationship between defect contents based on the Q_3/Q_4 ratio and PO selectivity of tandem propene epoxidation with H_2 and O_2.

As the defect concentration, determined from the Q_3/Q_4 ratio in the ^{29}Si NMR spectra, increased from 5.3% to 19.8%, the initial PO formation rate progressively decreased from 340 to 145 $g_{PO}h^{-1}kg_{Cat}^{-1}$, accompanied by a decrease in PO selectivity from 81% to 64%. These results further demonstrate that defect healing facilitates PO desorption by weakening hydrogen-bond interactions between PO and hydroxyl defects, thereby suppressing ring-opening and overoxidation side reactions.

The variation in the Q_3/Q_4 ratio agrees well with the trends observed by ^{1}H NMR and NH_3-TPD, consistently confirming the progressive reduction of hydroxyl defects. Nevertheless, the latter two techniques may be influenced by factors such as adsorbed water, hydrogen bonding, NH_3 adsorption/desorption and diffusion, making direct quantitative correlation with catalytic performance less straightforward. Therefore, the Q_3/Q_4 ratio derived from ^{29}Si NMR, which has been widely used as a descriptor of silanol defects in zeolite frameworks, was employed to quantify the relative defect concentration.

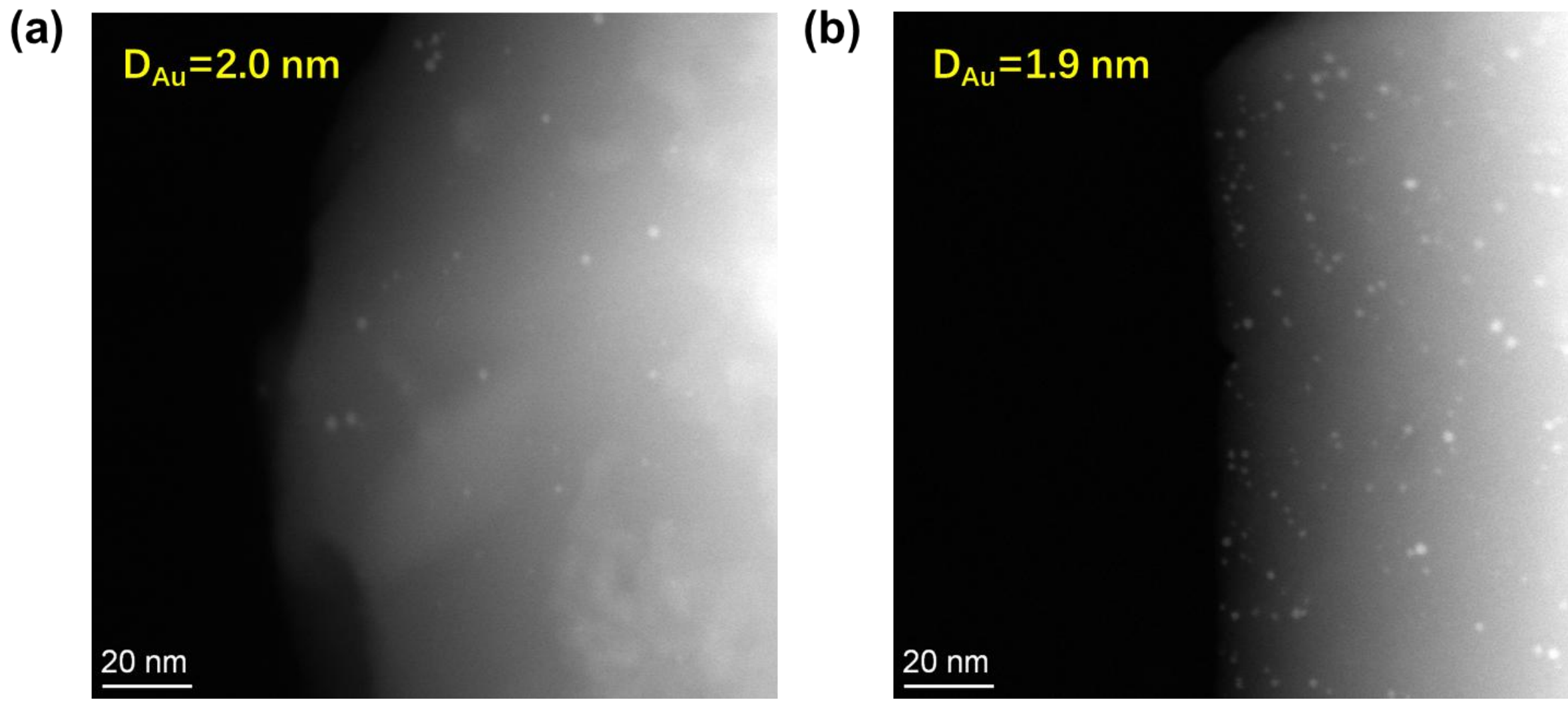

Figure S19 The typical STEM images of (a) Au/G-TS-1 and (b) Au/L-TS-1.

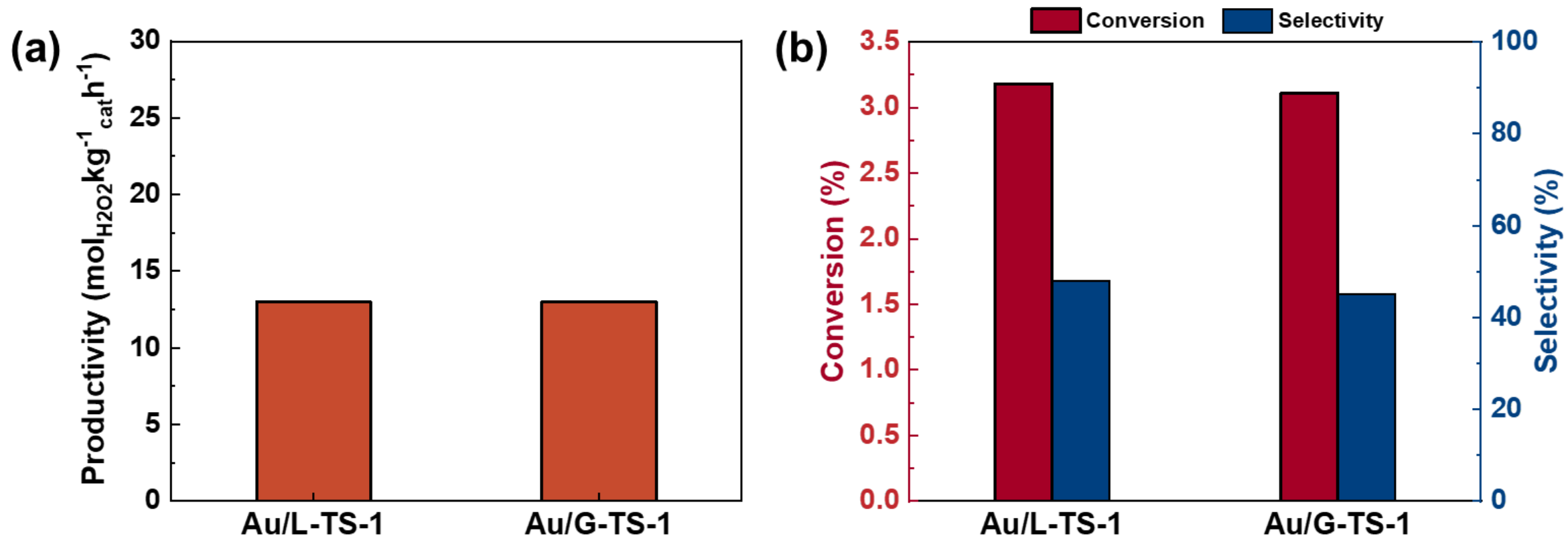

Figure S20 The (a) productivity of H_2O_2, (b) conversion of H_2 and selectivity of H_2O_2 by direct synthesis between H_2 and O_2 on different Au/TS-1 catalysts.

H_2O_2 synthesis conditions: Catalyst (0.01g), H_2O (2.9 g), MeOH (5.6 g), 5% H_2/CO_2 (420 psi), 25% O_2/CO_2 (160 psi), reaction time 0.5 h, reaction temperature 2 °C, stirring speed 1200 rpm.

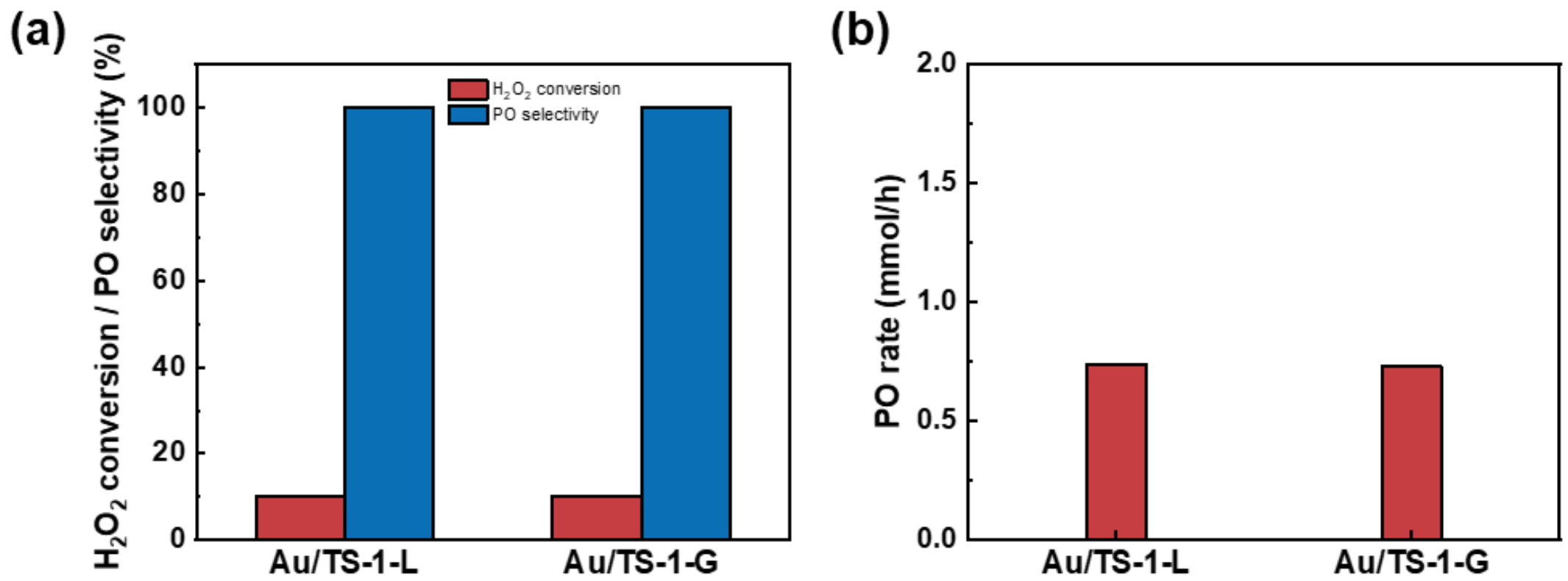

Figure S21 The (a) conversion of H_2O_2, PO selectivity and (b) PO formation rate by ex-situ propene epoxidation on different Au/TS-1 catalysts.

Ex-situ propene epoxidation conditions: Catalyst (0.05g), H_2O_2 (0.8 g), MeOH (8.0g), propene (0.4 MPa), reaction time 1 h, reaction temperature 40 °C, stirring speed 800 rpm.

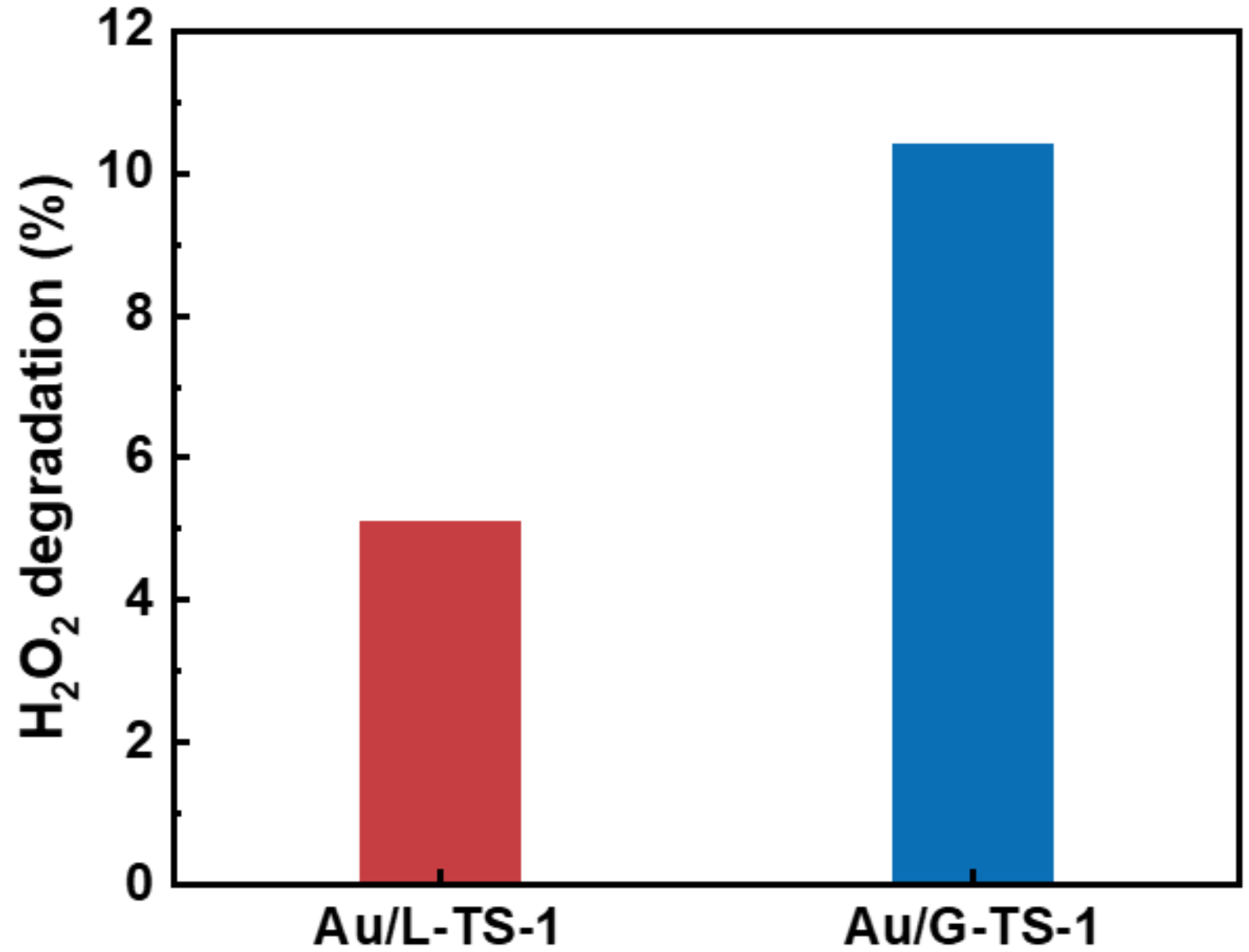

Figure S22 The degradation of H_2O_2 on different Au/TS-1 catalysts.

The degradation of H_2O_2: catalyst (0.2 g), acetonitrile (20 mL), H_2O_2 (30 wt%, 1.6 g), reaction temperature (60 ^oC), reaction time (1 h)

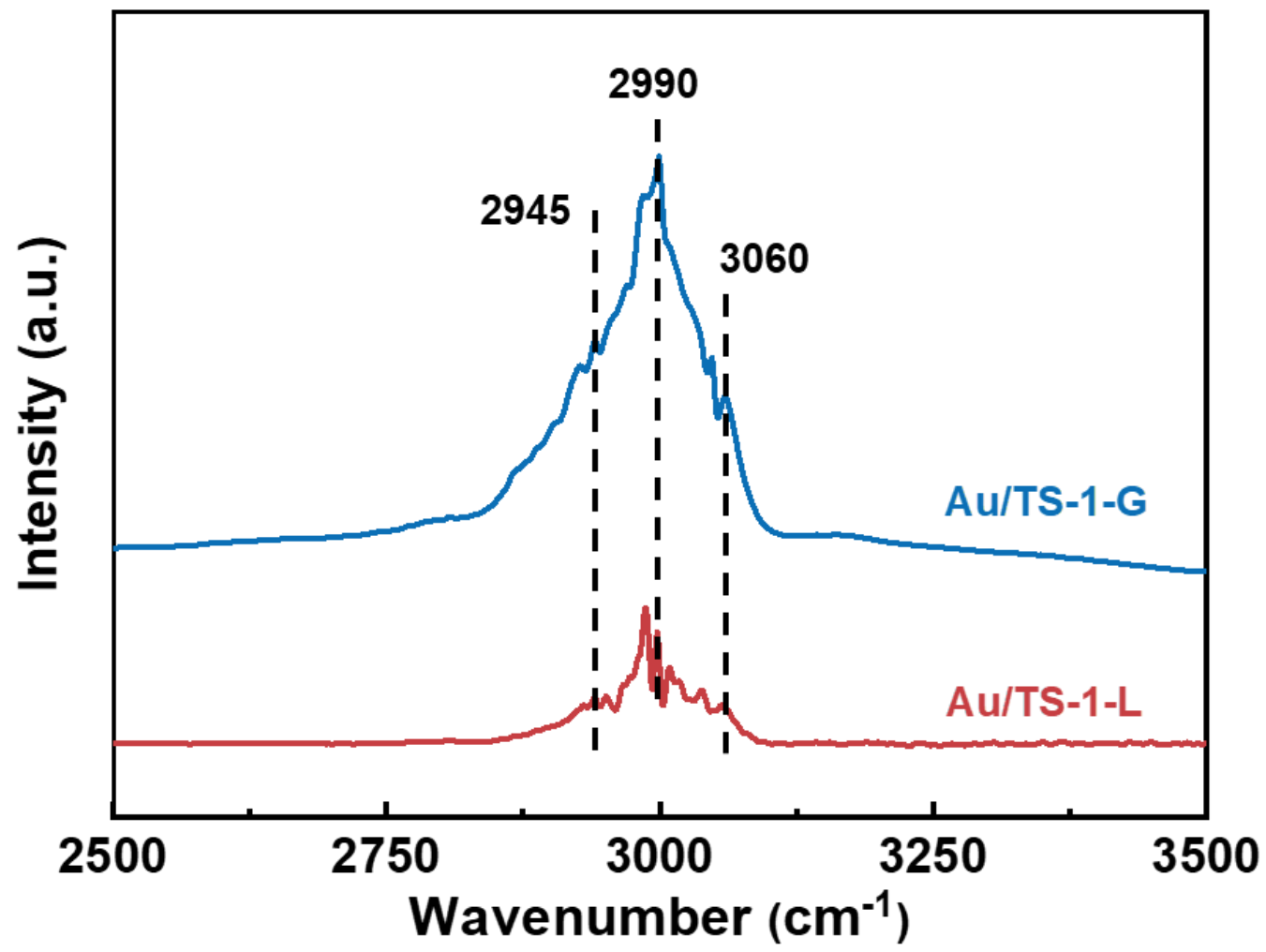

Figure S23 The PO DRIFT on different Au/TS-1 catalysts.

The bands at 2945 and 2990 cm^{-1} are assigned to the C-H stretching vibrations of the bidentate propoxy species, which can result from PO decomposition in the zeolite channels. The band at 3060 cm^{-1} is attributed to the CH_2 group of the adsorbed PO.

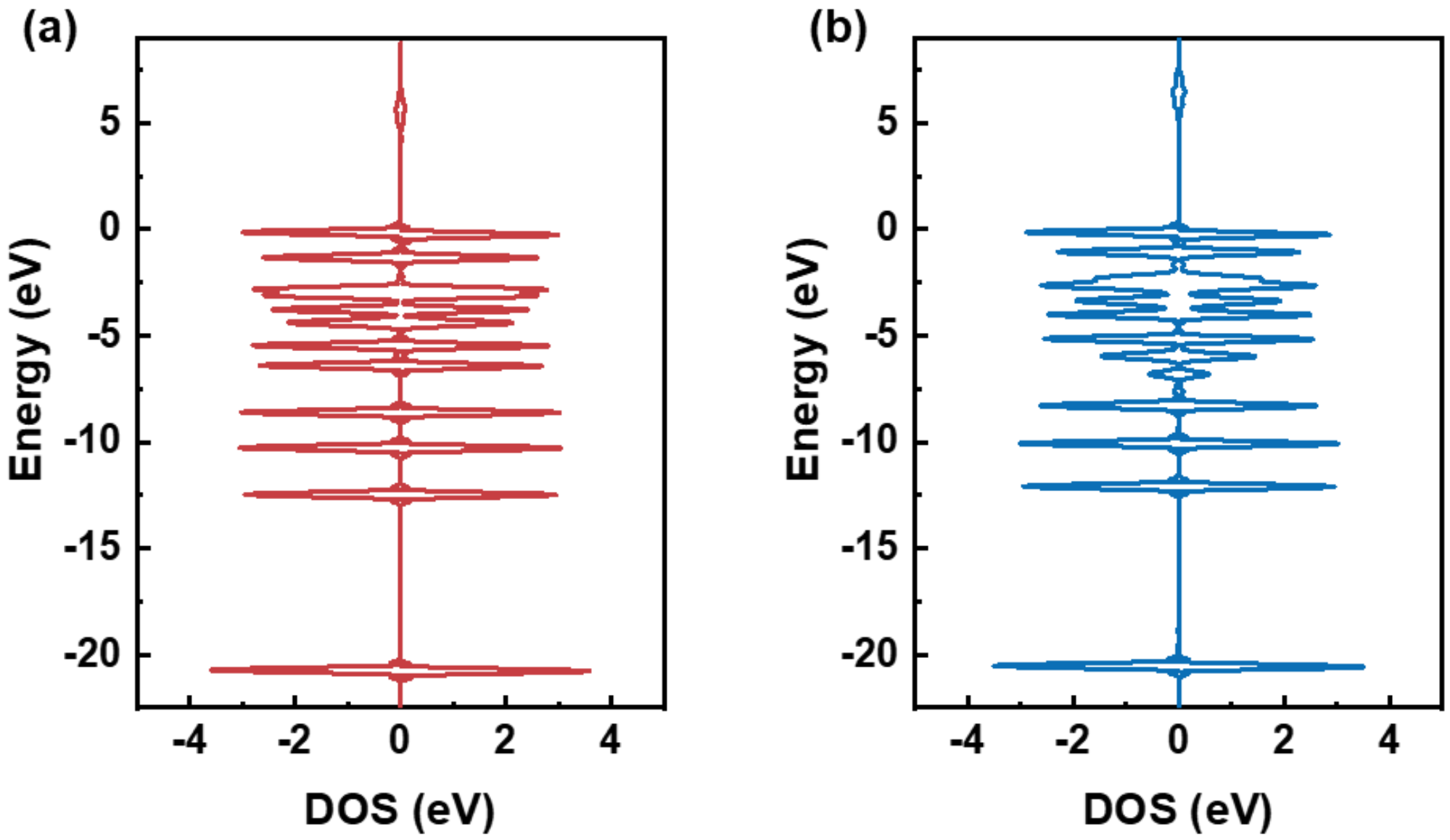

Figure S24 Total density of states (DOS) of PO adsorbed on (a) defect-healed TS-1 and (b) hydroxyl-rich TS-1-OH.

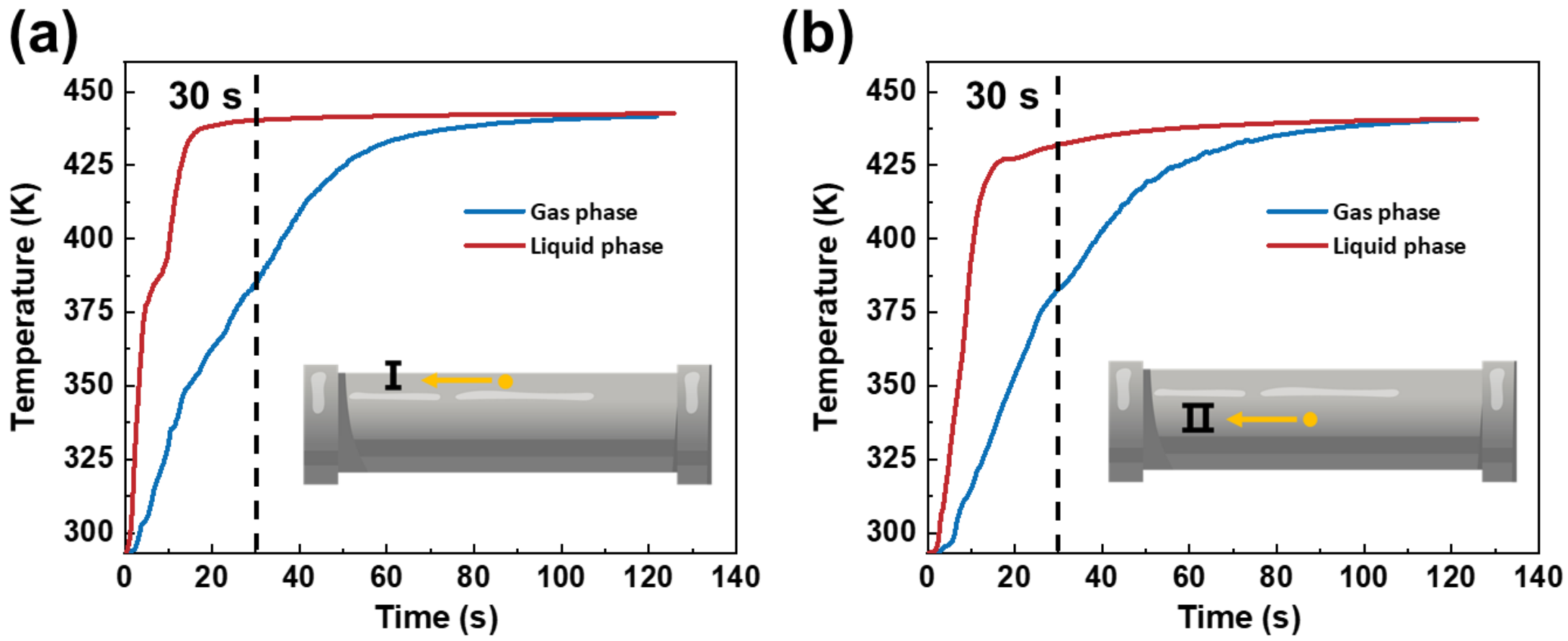

Figure S25 The temperature-time curves in oil (liquid phase) or air (gas phase) medium at different representative points (a) I, (b) II.

When using an oil heat-transfer environment, the temperatures at locations I, II and III reached 432~443 K in 30 s. In contrast, the temperatures at position I, II and III are much lower (i.e., 383~402 K) at this timepoint when using air as the heat-transfer environment, with the lower heating rate in air attributed to the relatively lower heat transfer efficiency compared to that when using oil.

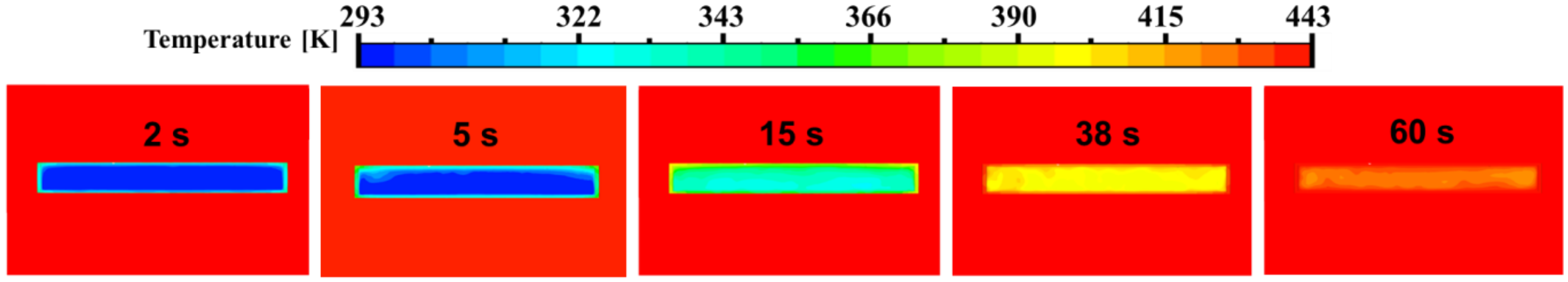

Figure S26 The two-dimensional diagram of temperature over time for the cross section of the tube in heat transfer media of air.

The temperature in the tube was gradually increased from the wall to the centre during the heat transfer process.

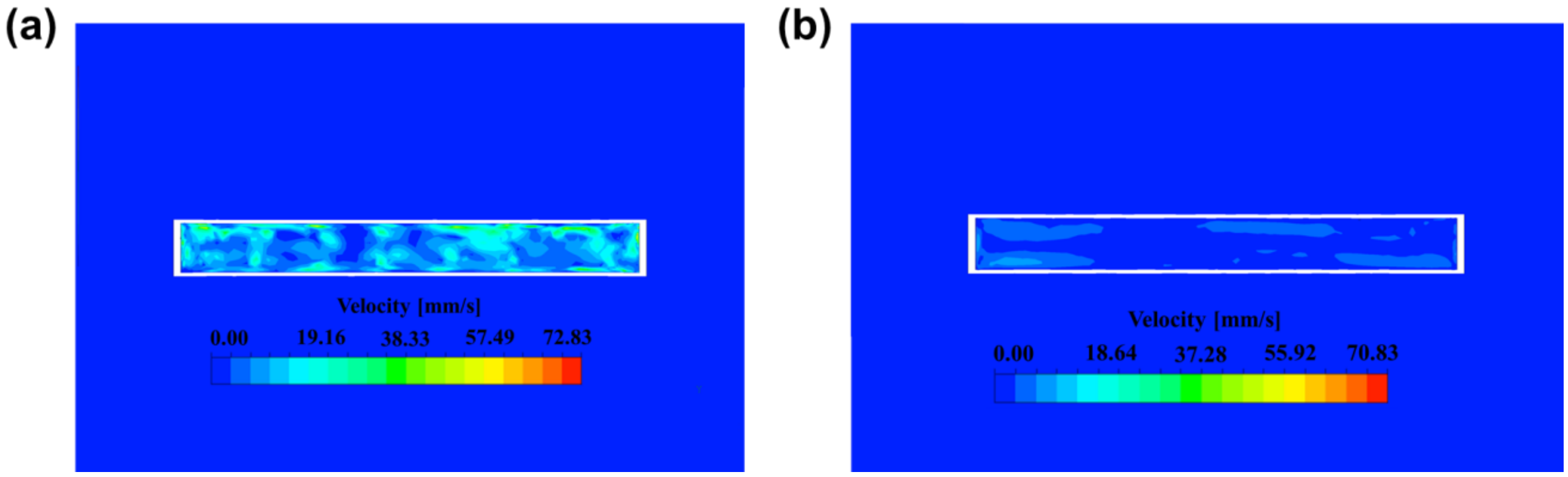

Figure S27 The two-dimensional diagram of flow velocity after heating 5 s for the cross section of the tube in the heat transfer media of (a) oil and (b) air.

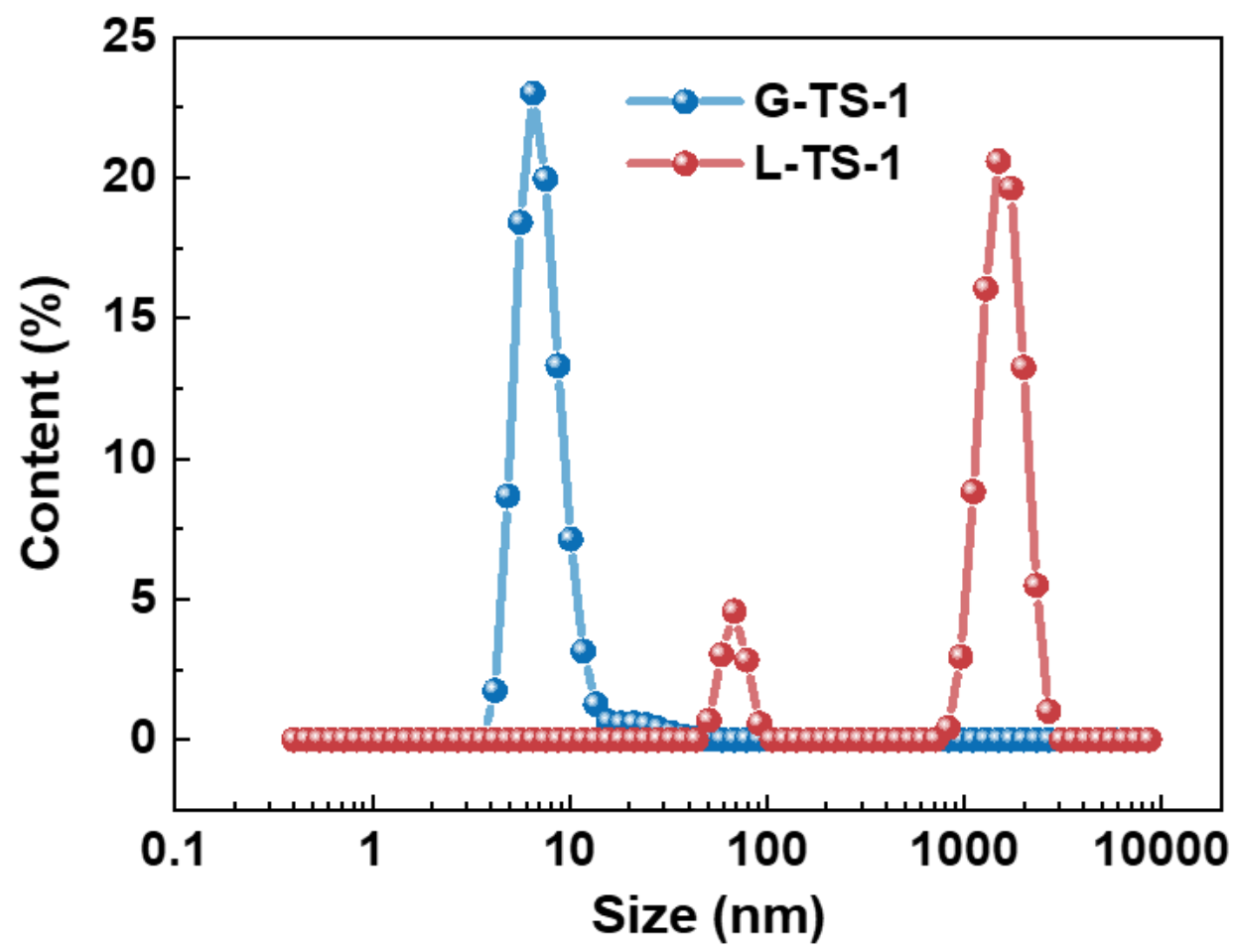

Figure S28 The dynamic light scattering-particle size distributions (DLS-PSD) of G-TS-1 and L-TS-1 at the crystallization time of 30 minutes.

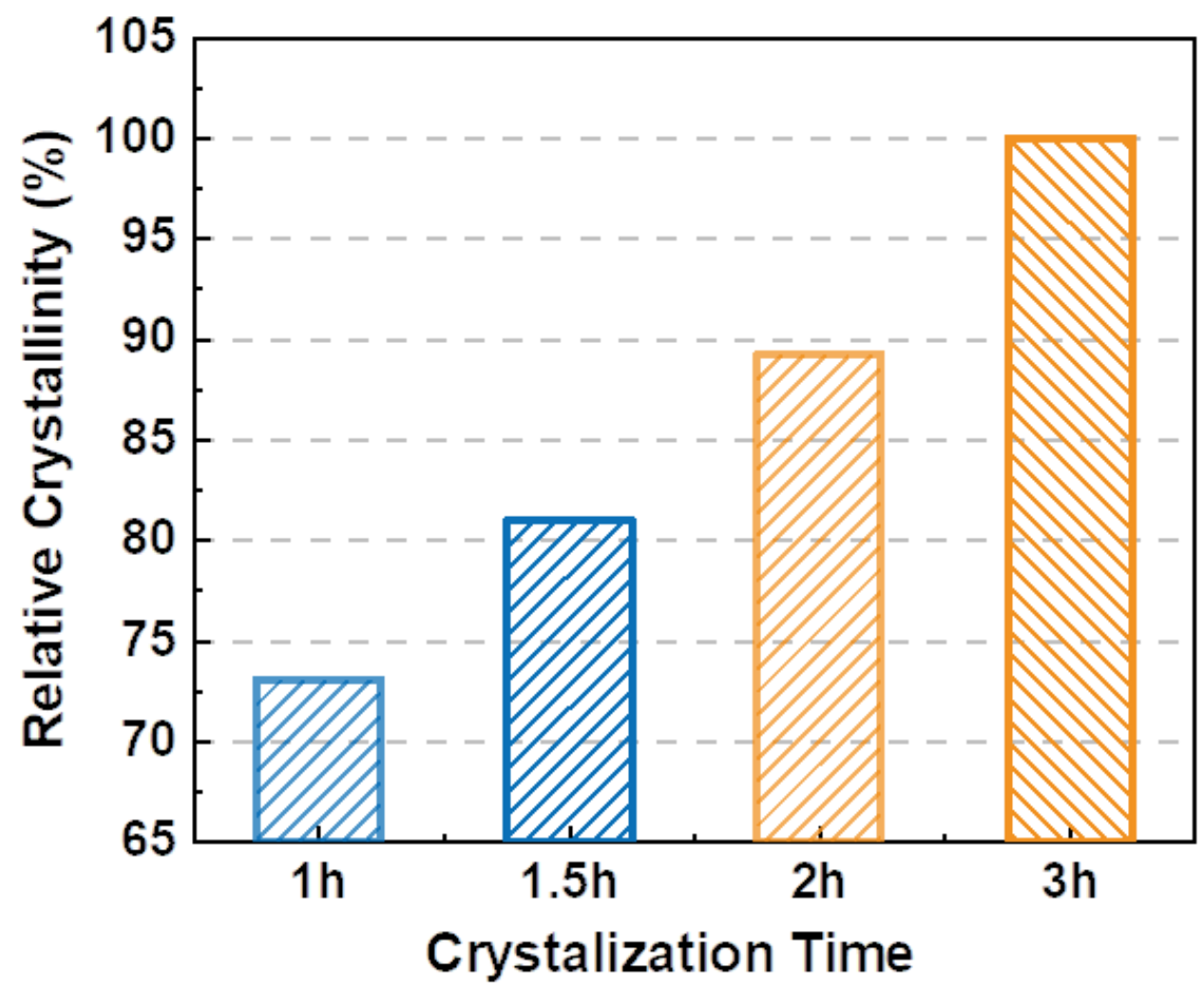

Figure S29 The relative crystallinity of titanosilicalites crystallized at different time in the heat transfer medium of oil.

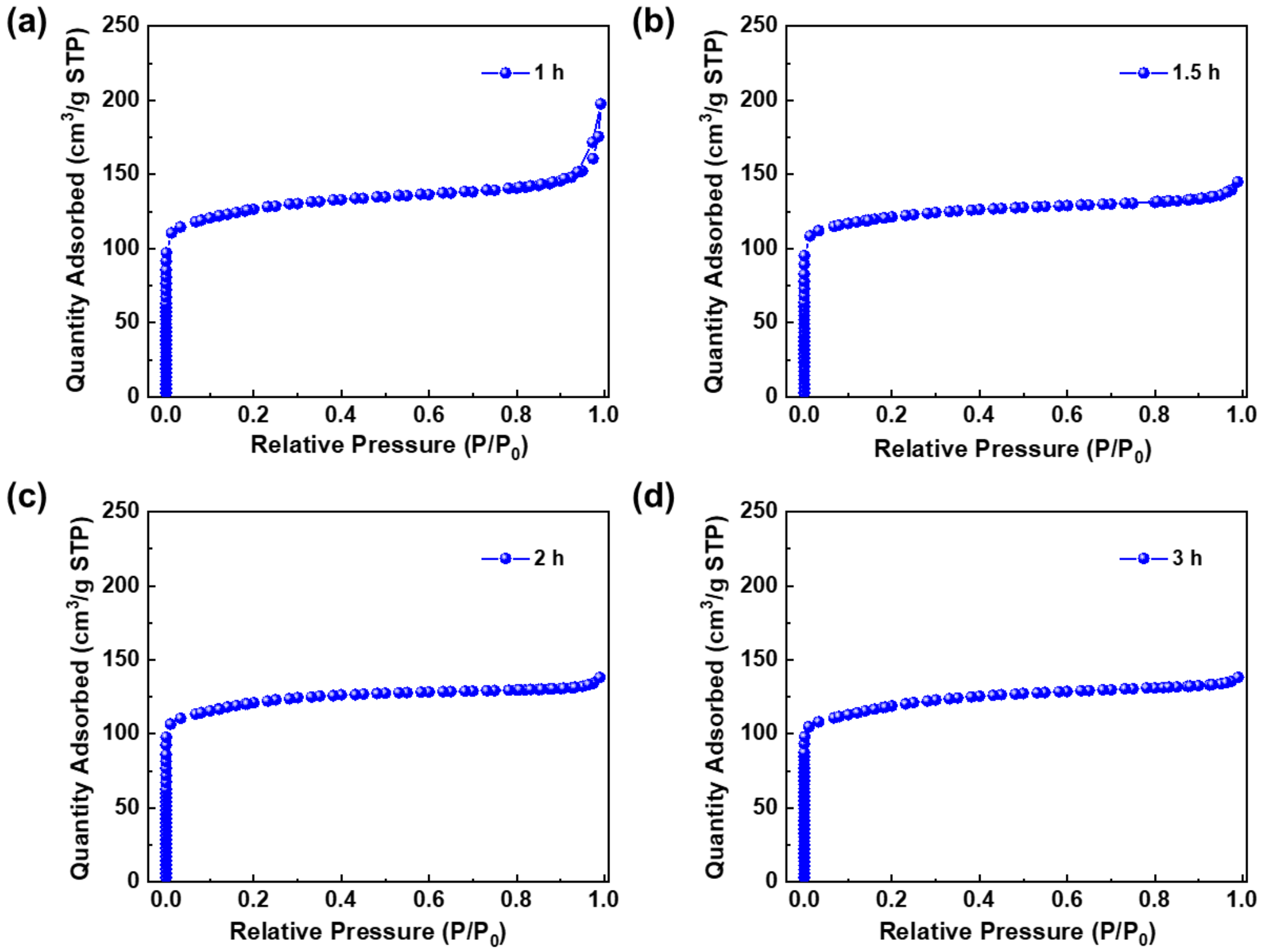

Figure S30 The nitrogen adsorption and desorption isotherms of titanosilicalites crystallized at (a) 1 h, (b) 1.5 h, (c) 2 h and (d) 3 h in the heat transfer medium of oil.

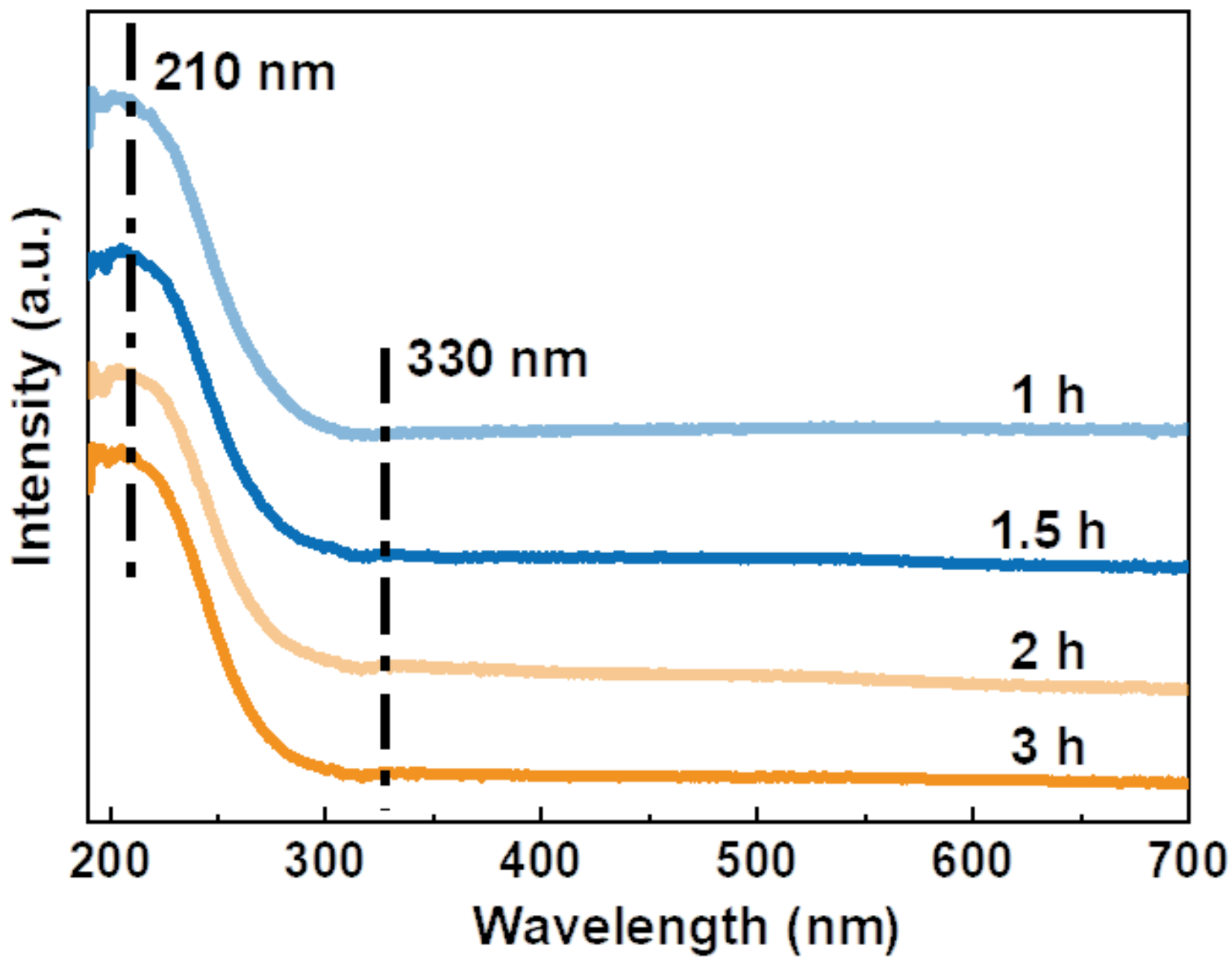

Figure S31 The UV-vis spectra of titanosilicalites crystallized at different time in the heat transfer medium of oil.

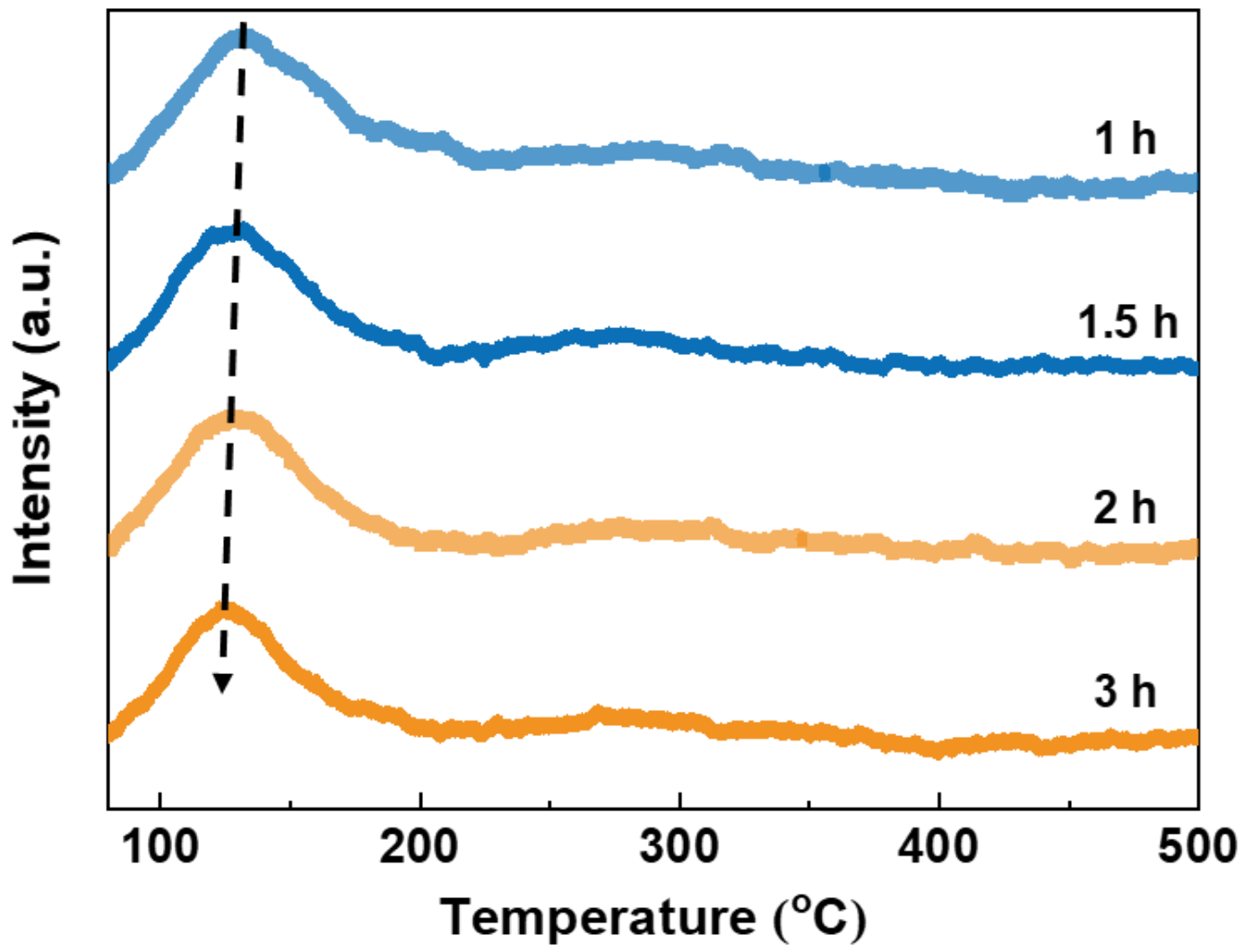

Figure S32 The NH_3-TPD patterns of titanosilicalites crystallized at different time in the heat transfer medium of oil.

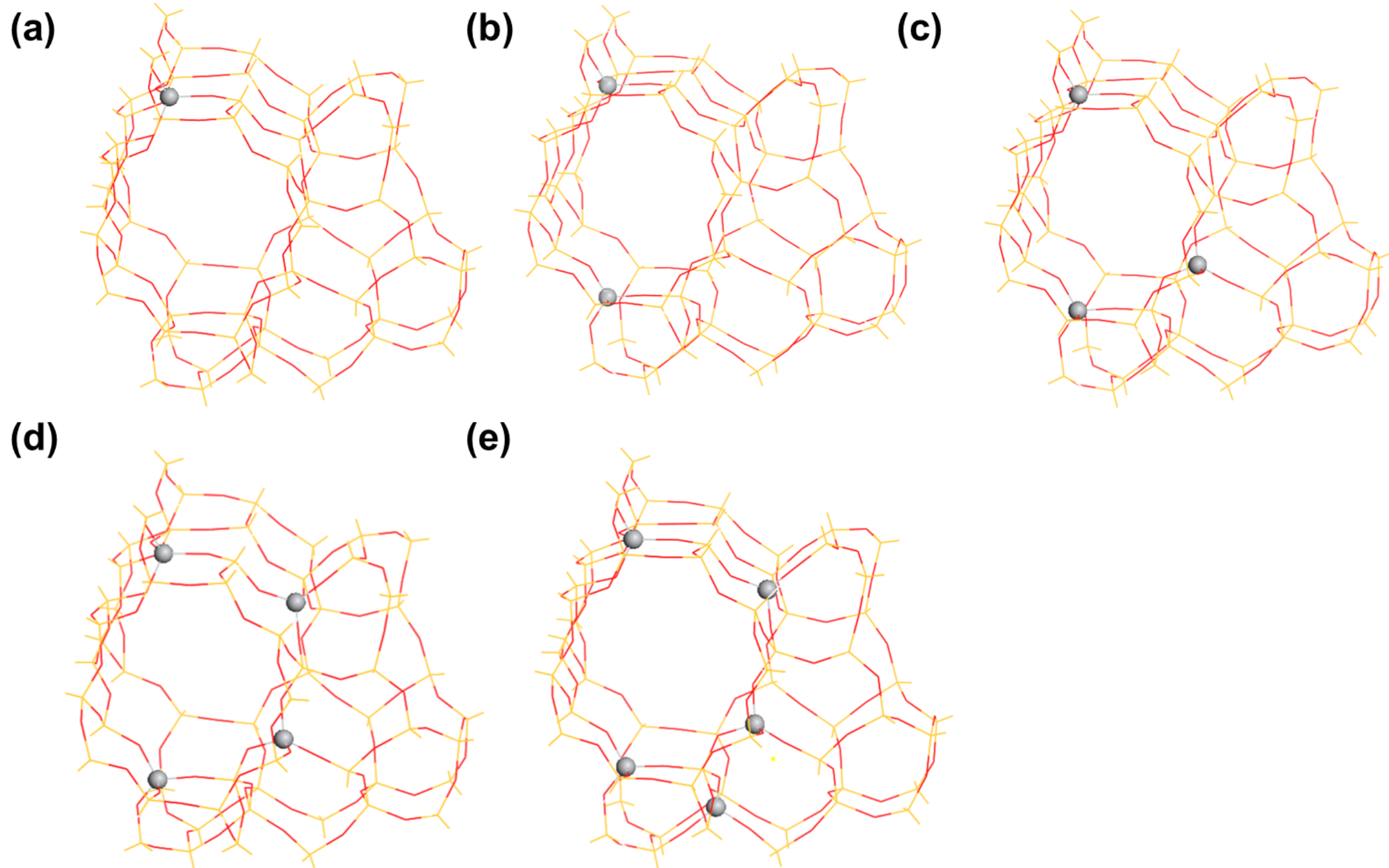

Figure S33 The MFI cluster with (a) one, (b) two, (c) three, (d) four and (e) five Ti atoms in the framework.

The Ti-rich core implies that as many as two Ti atoms may be incorporated per unit cell. DFT calculations support the energetic favorability of this configuration. Furthermore, the negative formation energy for incorporating a single Ti atom confirms that Ti insertion into the MFI framework is thermodynamically preferred.

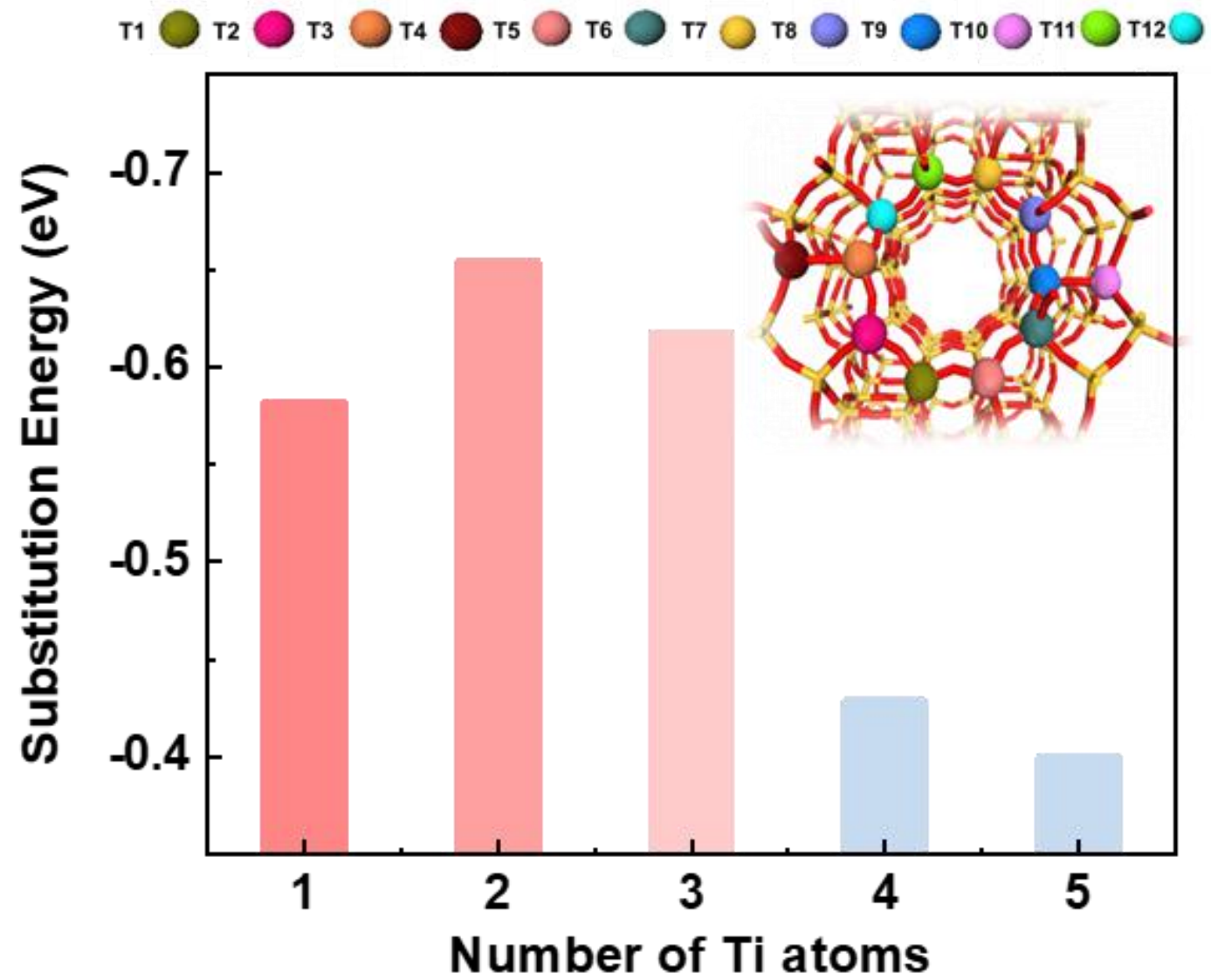

Figure S34 The substitution energy of different numbers of Ti atoms in the MFI framework. The inset shows the 12 inequivalent T sites of Si atoms in the MFI framework, represented by different colored spheres.

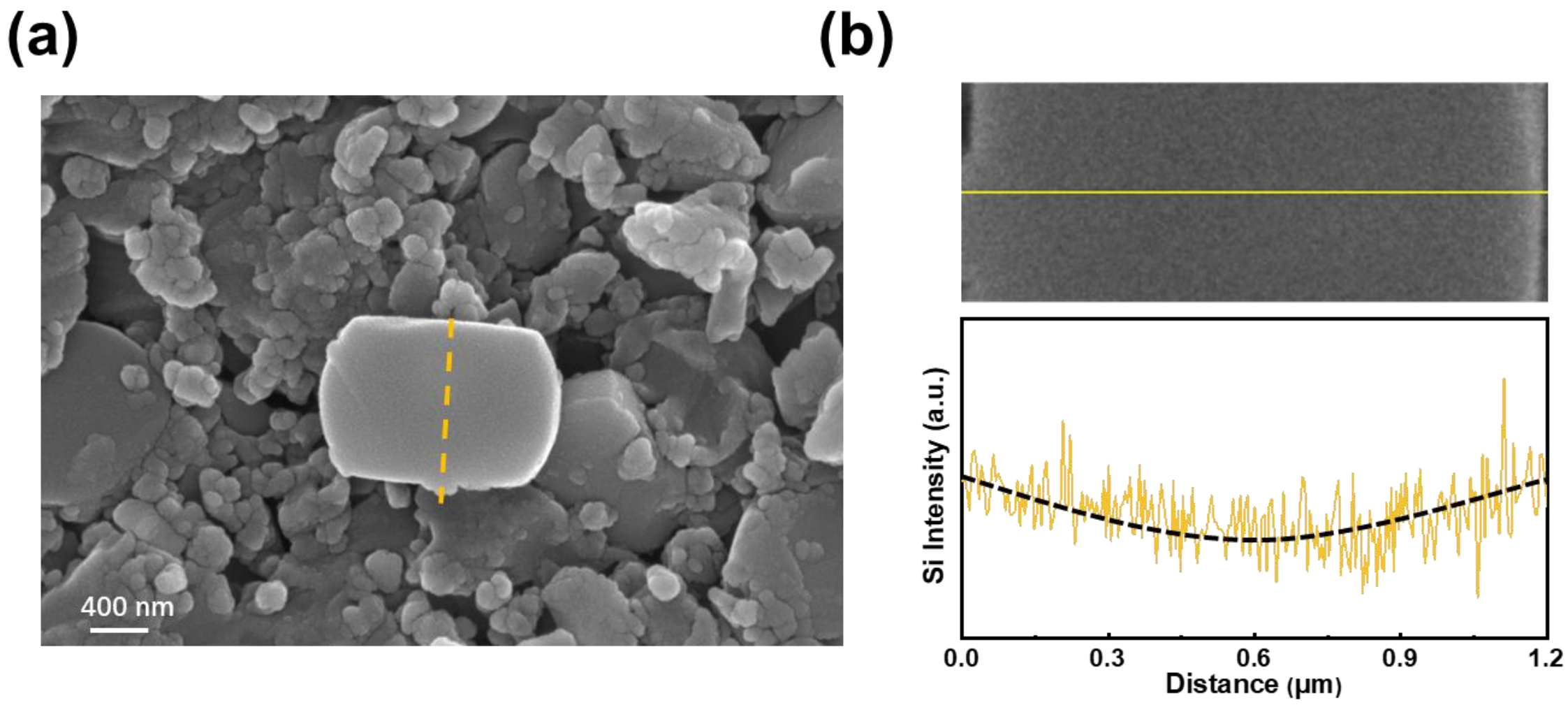

Figure S35 (a) The cross-section SEM images of titanosilicalites after crystallization of 3 h. (b) The SEM-EDS line-scan analysis of titanosilicalites after crystallization of 3 h.

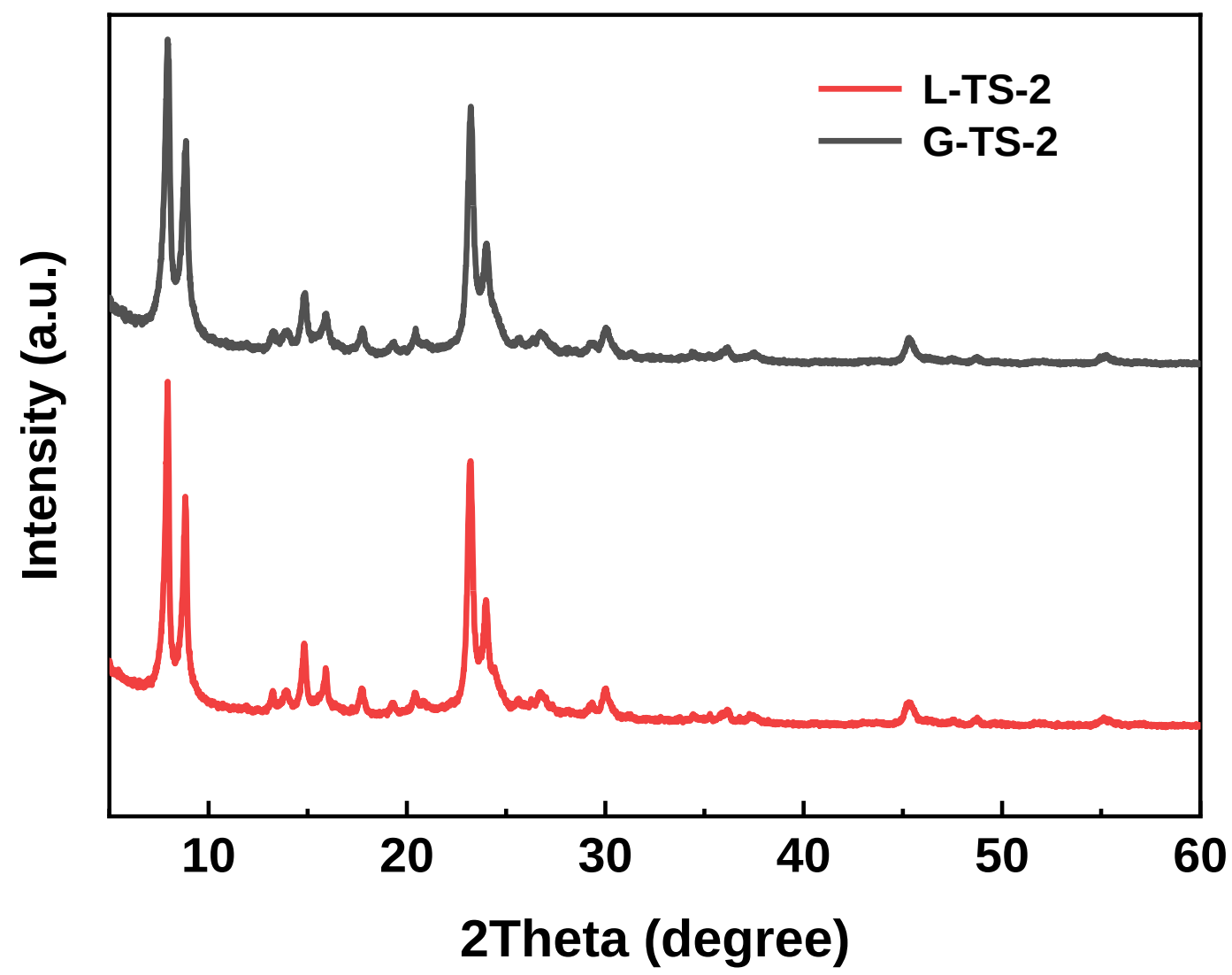

Figure S36 The XRD patterns of different titanosilicalite-2 (L-TS-2 crystallized in the heat transfer medium of oil at 170 ºC for 3 h, G-TS-2 crystallized in the heat transfer medium of air at 170 ºC for 72 h).

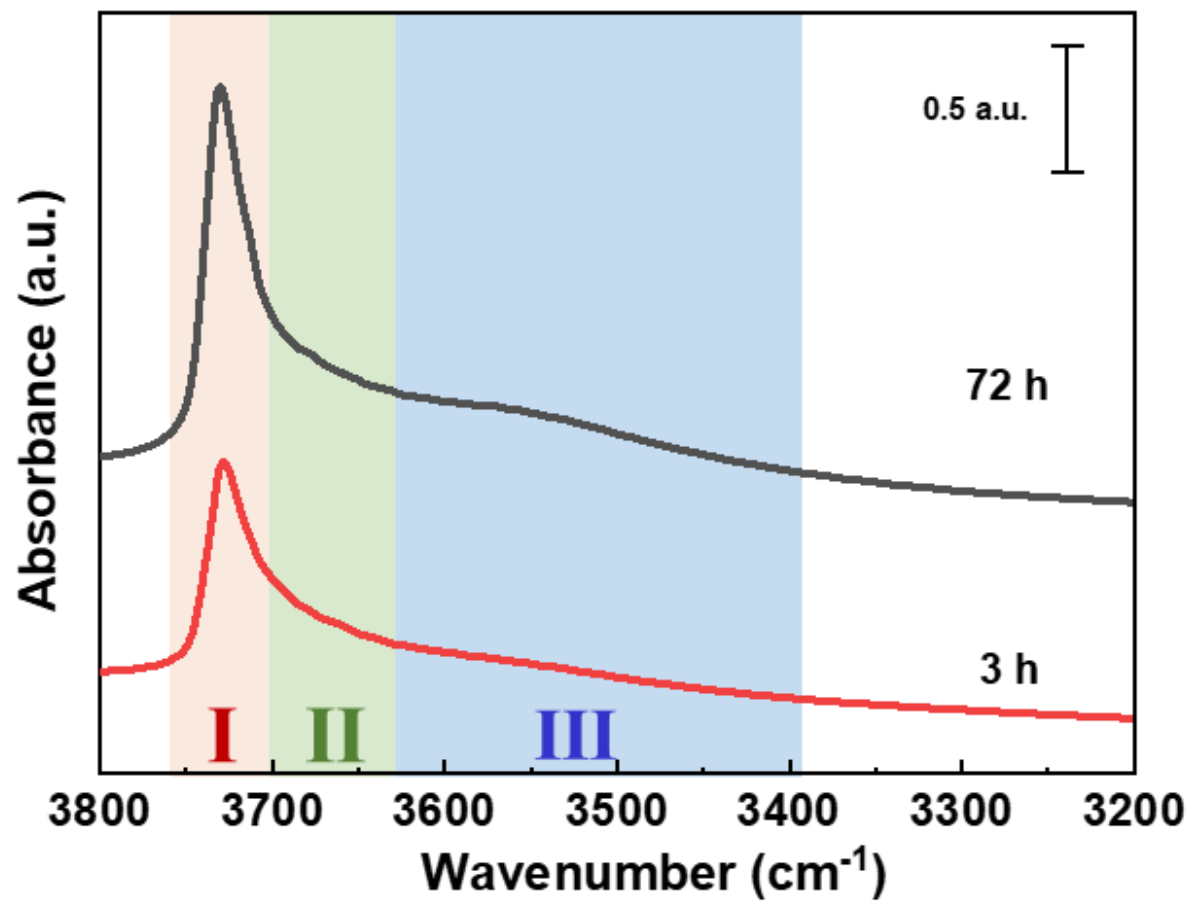

Figure S37 The FT-IR spectra of titanosilicalite-2 (L-TS-2 crystallized in the heat transfer medium of oil at 170 ^{o}C for 3 h, G-TS-2 crystallized in the heat transfer medium of air at 170 ^{o}C for 72 h) in the hydroxyl stretching region.

The hydroxyl stretching region I, II and III were attributed to the isolated external silanols, internal silanols, and hydrogen-bonded silanol nets. The results show that the L-TS-2 has less hydrogen-bonded silanol nets that G-TS-2.

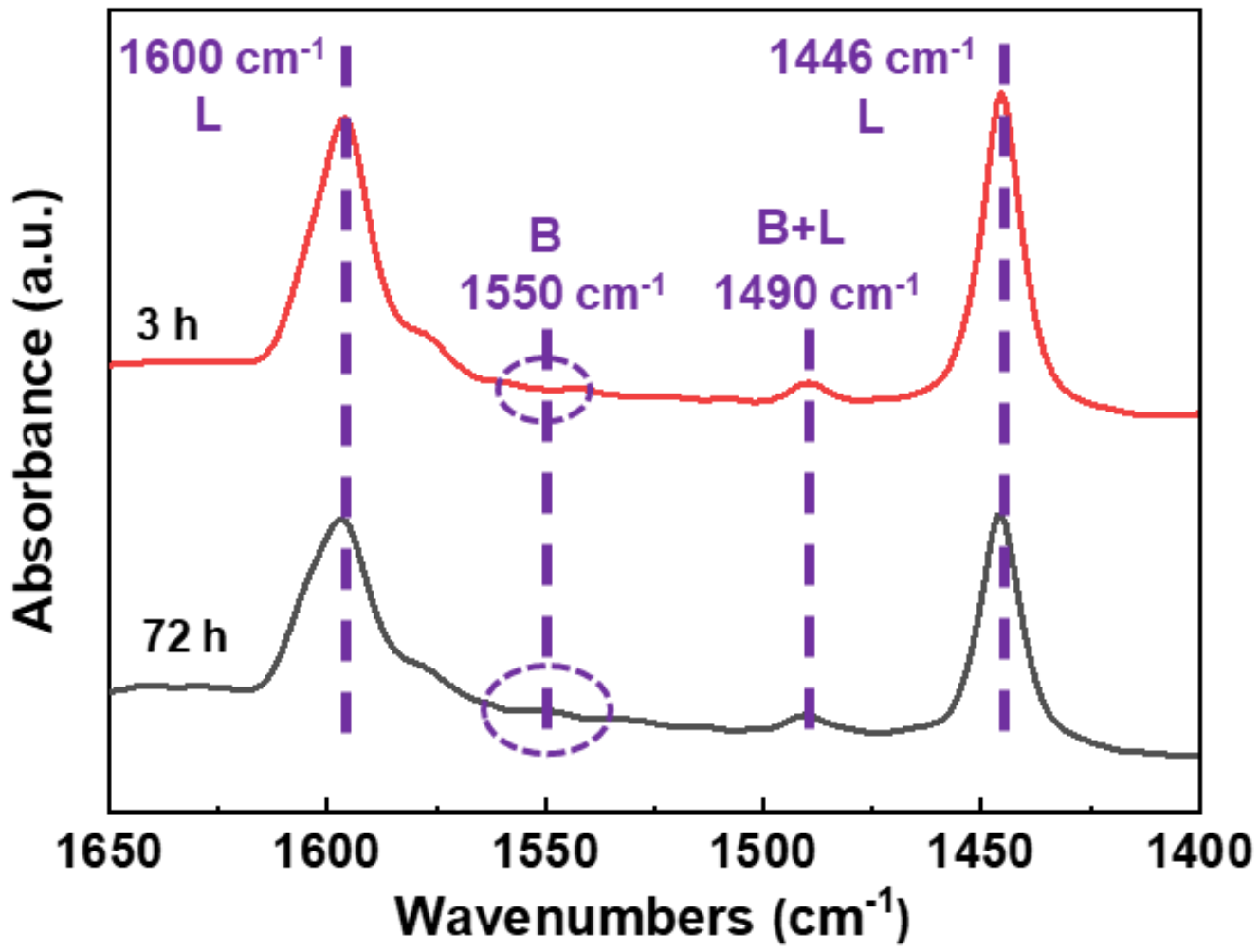

Figure S38 The Py-IR spectra of titanosilicalite-2 (L-TS-2 crystallized in the heat transfer medium of oil at 170 °C for 3 h, G-TS-2 crystallized in the heat transfer medium of air at 170 °C for 72 h).

The peaks at 1447 and 1601 cm^{-1} are attributed to Lewis acid sites (L). Moreover, the peaks at 1550 and 1490 cm^{-1} were ascribed to Brønsted acid sites (B) and Brønsted and Lewis acid sites (B+L), respectively. Both titanosilicalites show intense peaks of Lewis acid sites while only the G-TS-2 shows a weak Brønsted acid peak at 1550 cm^{-1} which could be attributed to the presence of small amounts of Ti-OH species.

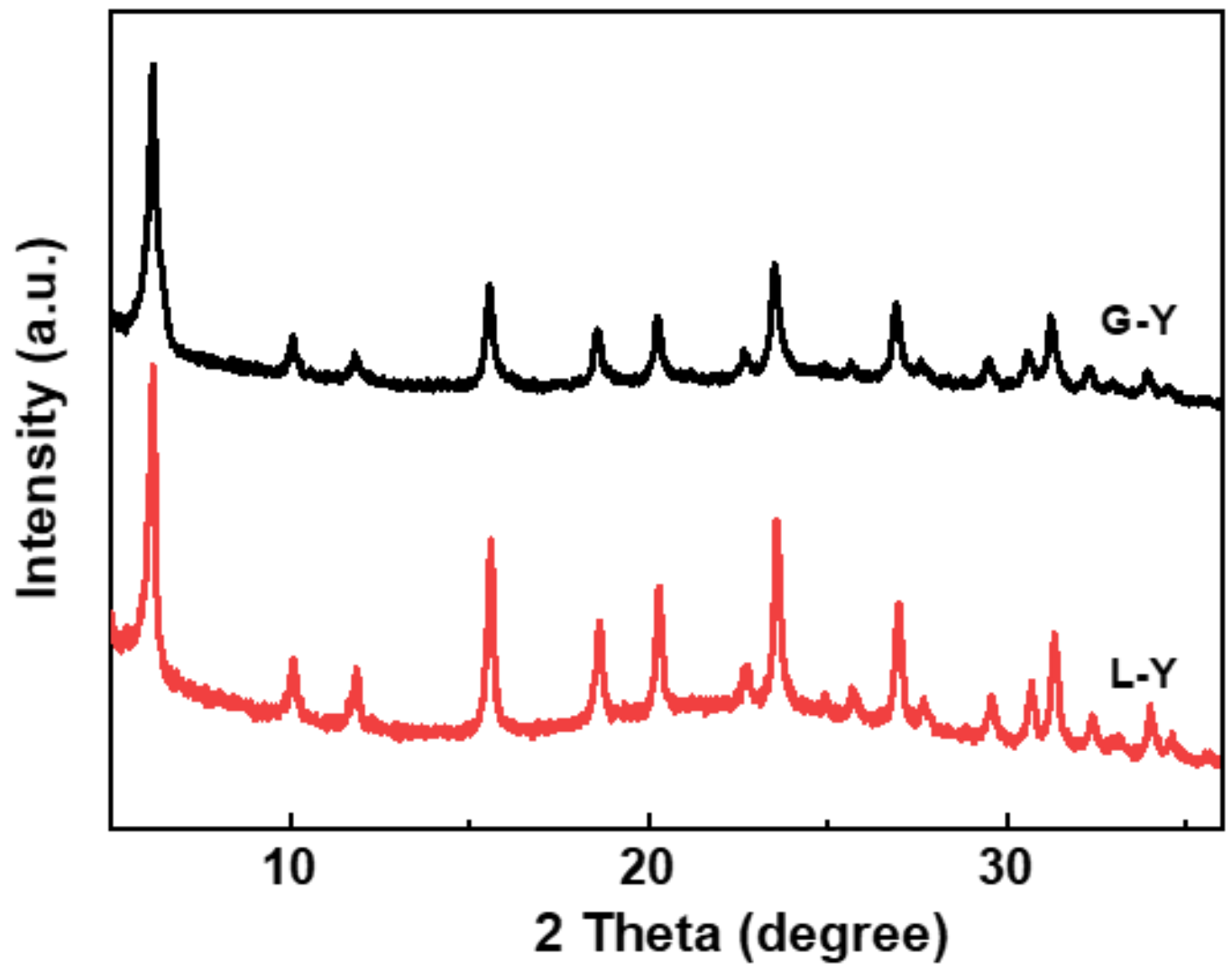

Figure S39 The XRD patterns of different Y zeolites (L-Y crystallized in the heat transfer medium of oil at 100 ^{o}C, G-Y crystallized in the heat transfer medium of air at 100 ^{o}C).

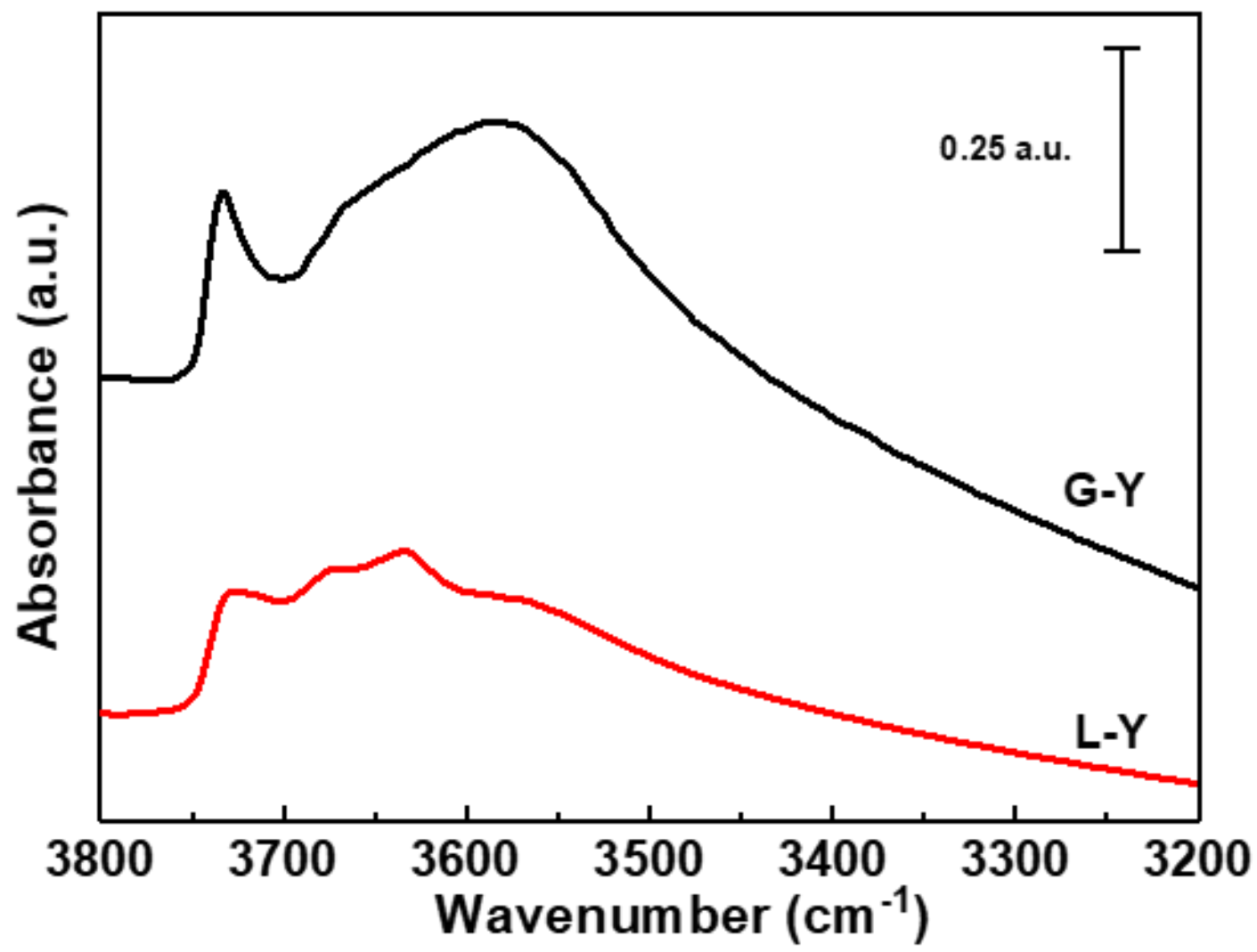

Figure S40 The FT-IR spectra of Y zeolites (L-Y crystallized in the heat transfer medium of oil at 100 ºC, G-Y crystallized in the heat transfer medium of air at 100 ºC) in the hydroxyl stretching region.

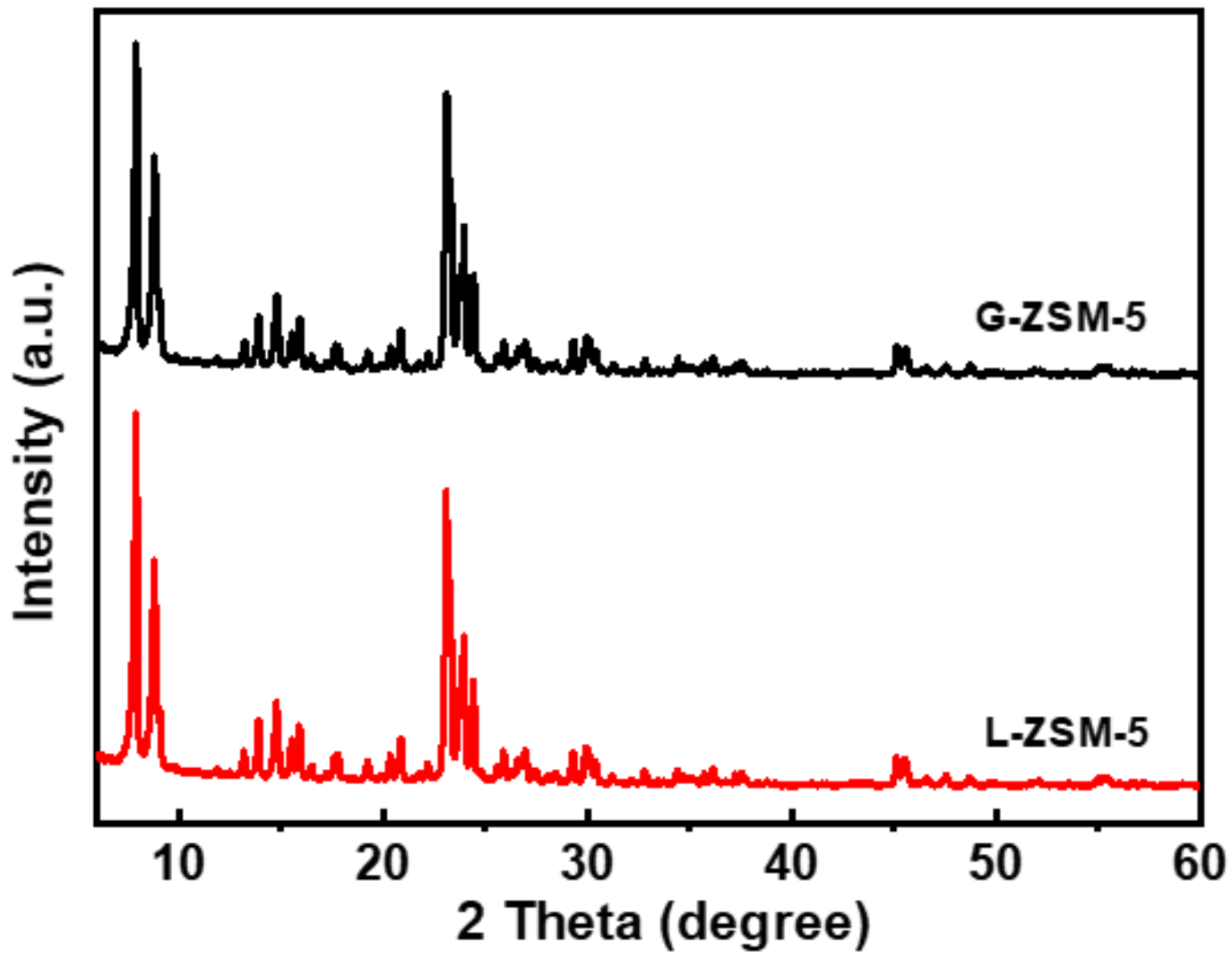

Figure S41 The XRD patterns of different ZSM-5 zeolites (L-ZSM-5 crystallized in the heat transfer medium of oil at 170 ºC, G- ZSM-5 crystallized in the heat transfer medium of air at 170 ºC).

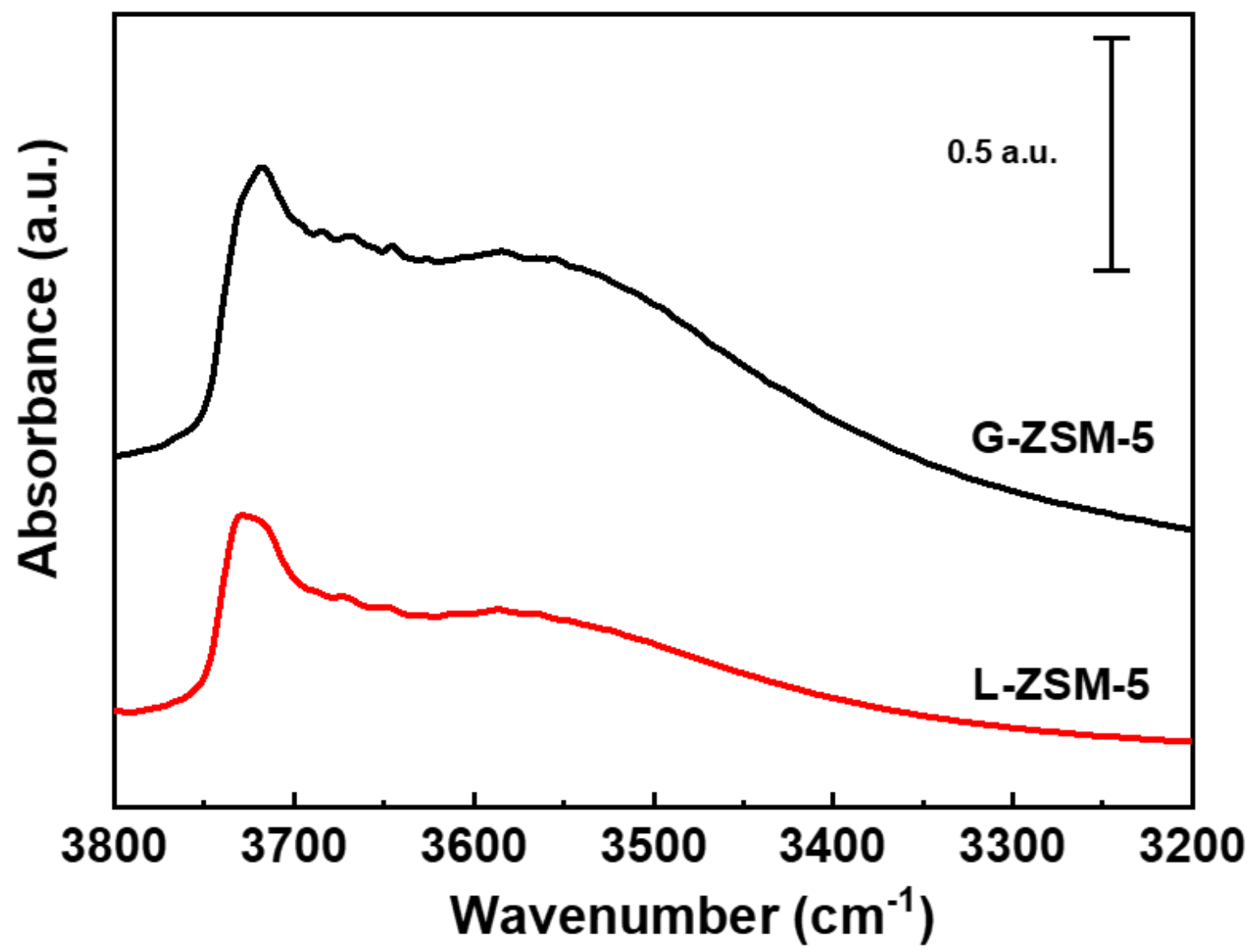

Figure S42 The FT-IR spectra of ZSM-5 zeolites (L-ZSM-5 crystallized in the heat transfer medium of oil at 170 ^oC, G- ZSM-5 crystallized in the heat transfer medium of air at 170 ^oC) in the hydroxyl stretching region.

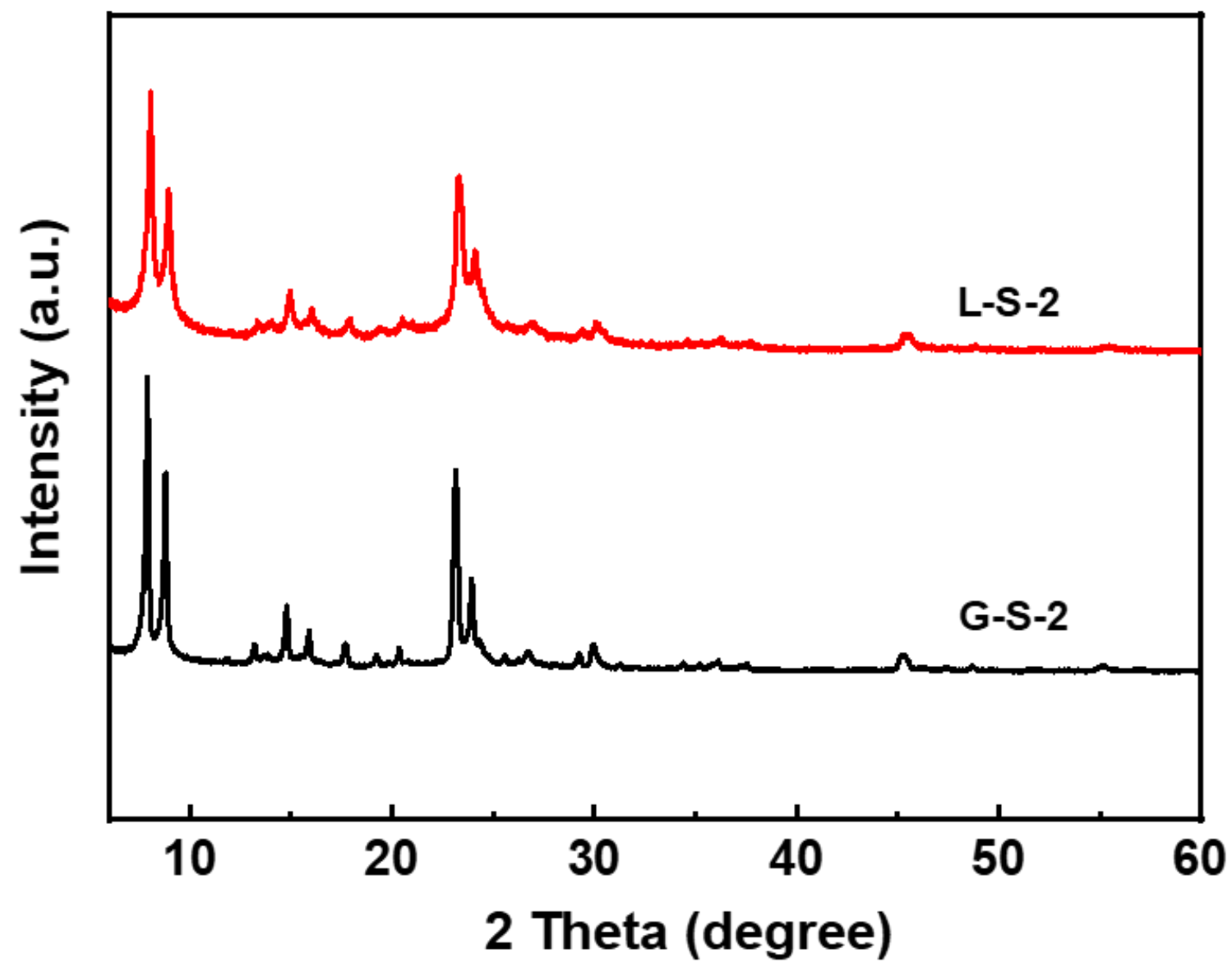

Figure S43 The XRD patterns of different silicalite-2 (L-S-2 crystallized in the heat transfer medium of oil at 170 ^{o}C, G-S-2 crystallized in the heat transfer medium of air at 170 ^{o}C).

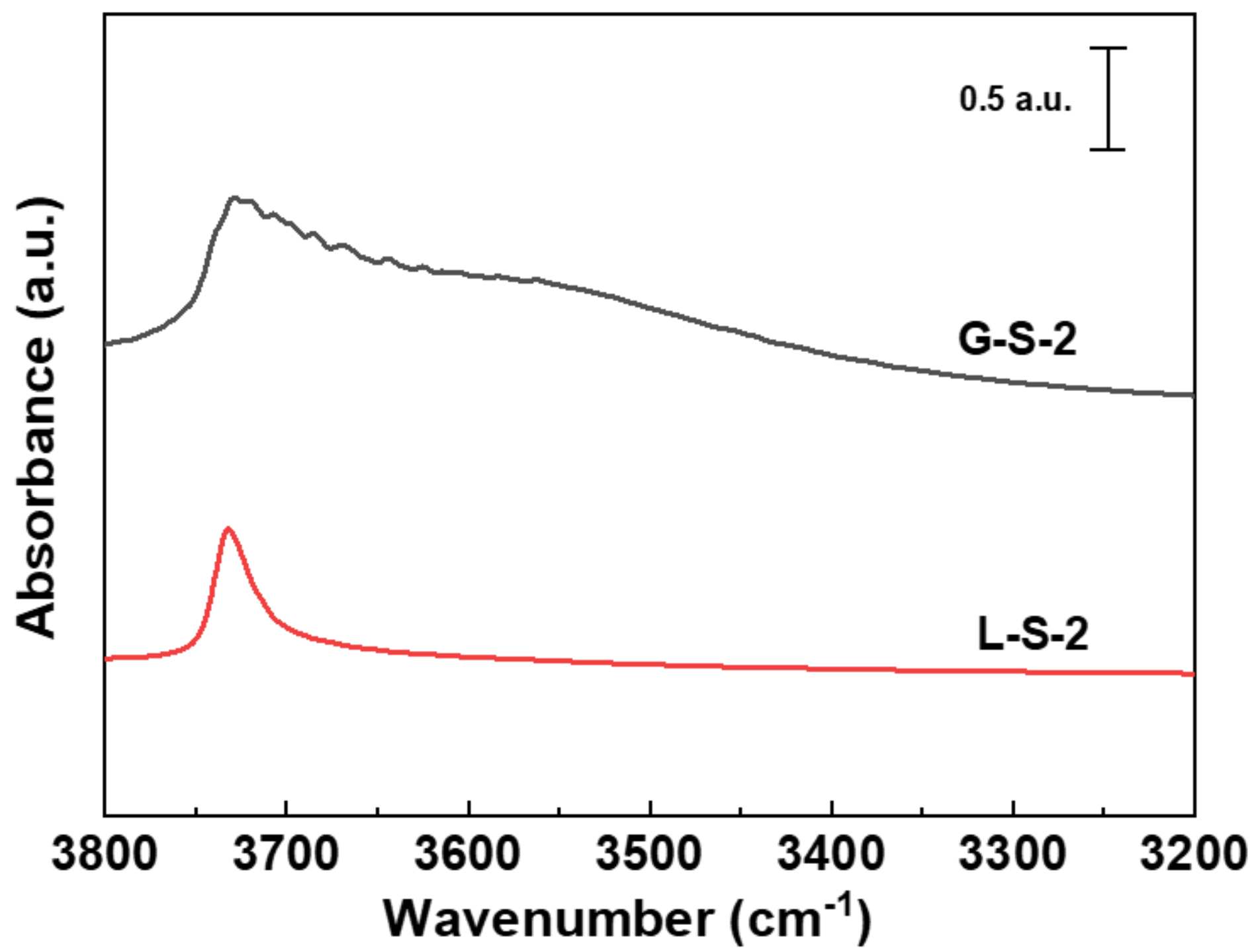

Figure S44 The FT-IR spectra of silicalite-2 (L-S-2 crystallized in the heat transfer medium of oil at 170 ºC, G-S-2 crystallized in the heat transfer medium of air at 170 ºC) in the hydroxyl stretching region.

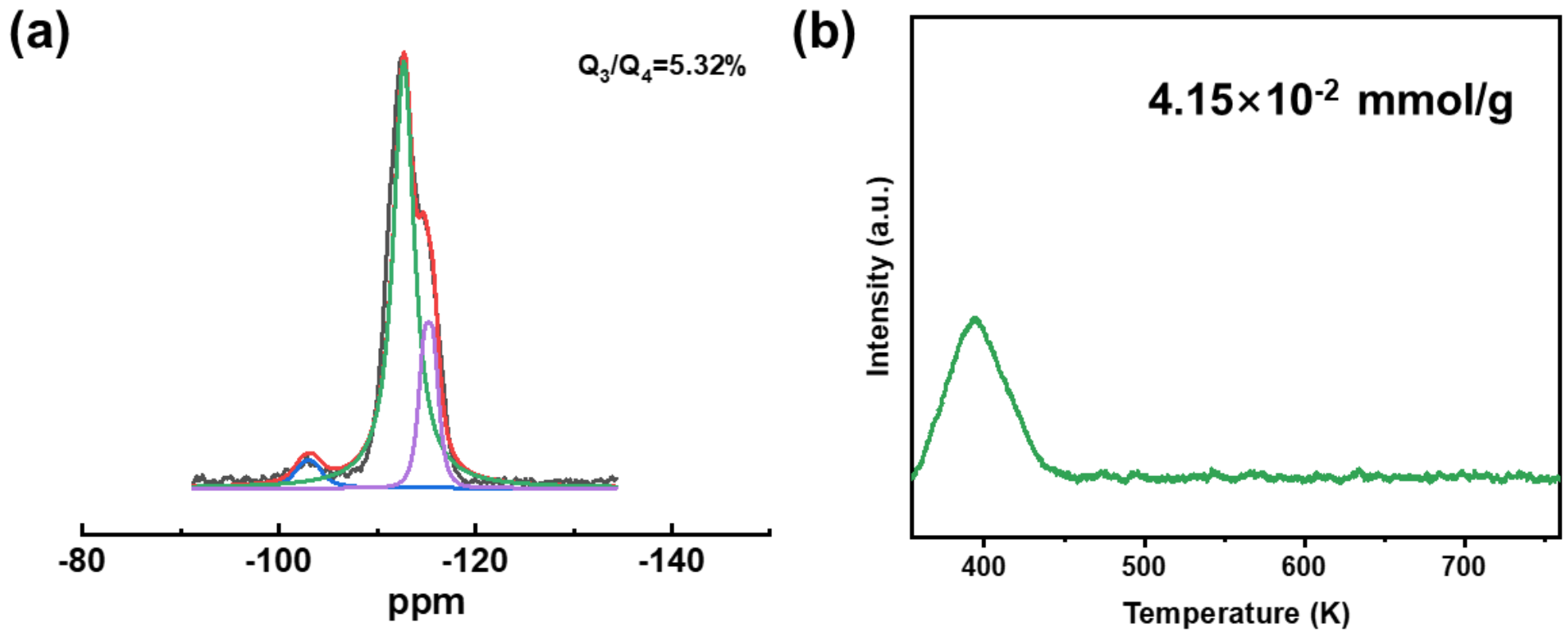

Figure S45 The (a) ^{29}Si MAS NMR and (b) NH_3-TPD of the scale-up titanosilicalite.

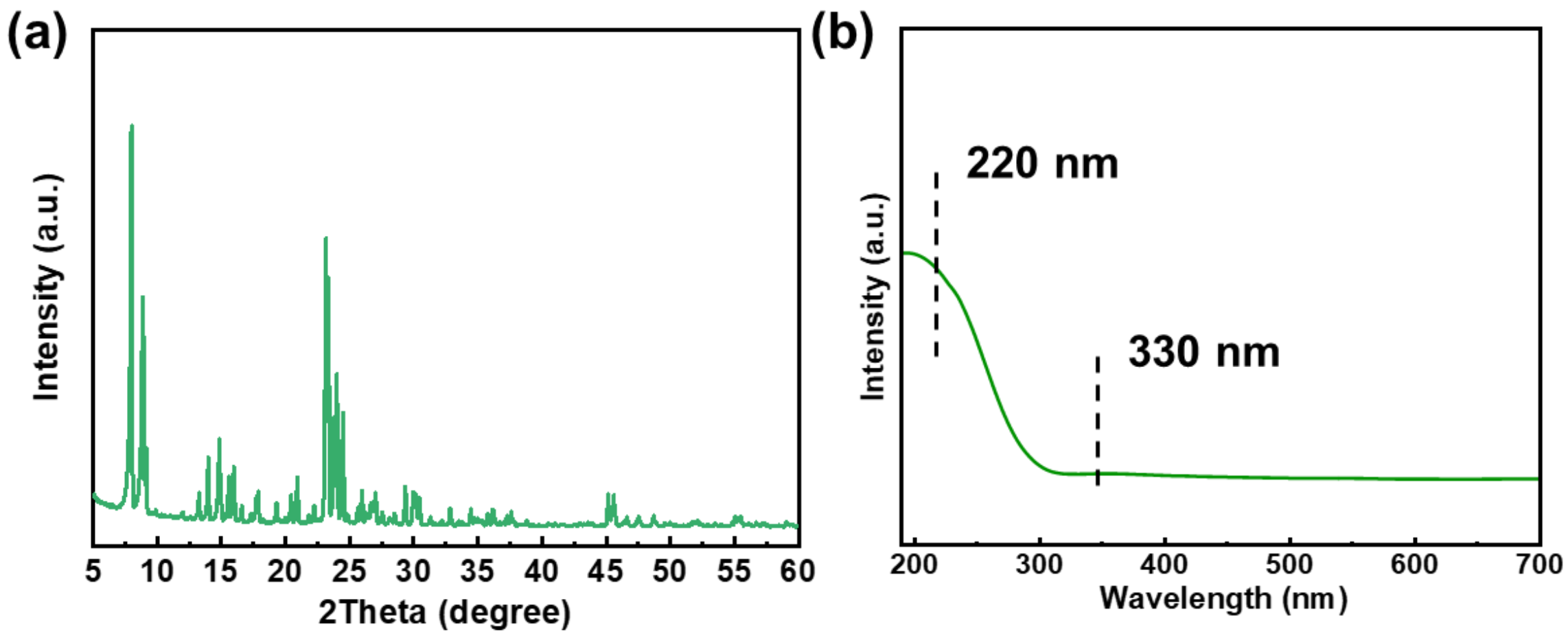

Figure S46 The (a) XRD and (b) UV-vis spectrum of the scale-up titanosilicalite.

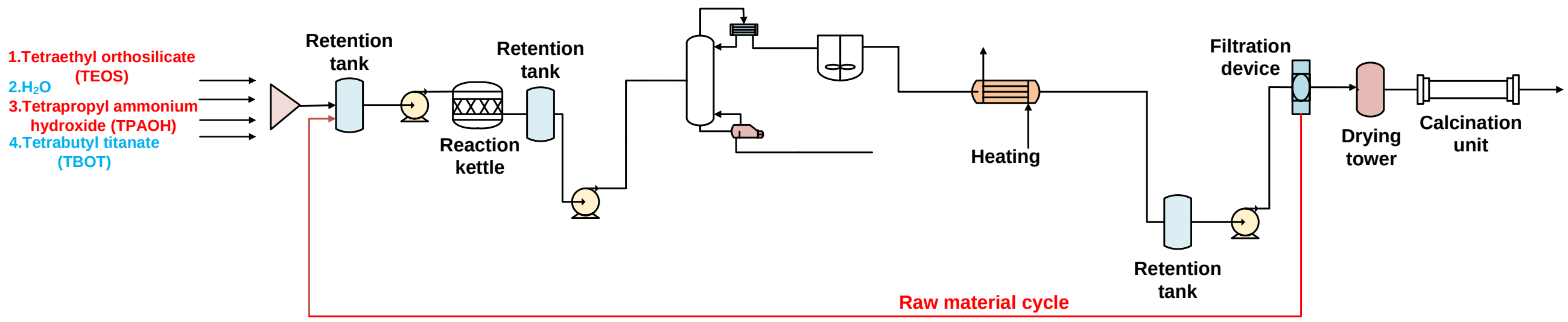

Figure S47 Flow diagrams of two routes for synthesizing TS-1 based on oil or air heating.

A comparative techno-economic and carbon-emission analysis of two TS-1 synthesis routes, the liquid-phase (Route 1) and gas-phase (Route 2) thermal environment, was performed using Aspen Plus, targeting TS-1 samples with comparable crystallinity but distinct crystallization times and yields. The process involves mixing raw materials (TEOS, TBOT and TPAOH) with water, followed by reaction, distillation, crystallization, filtration, and calcination.

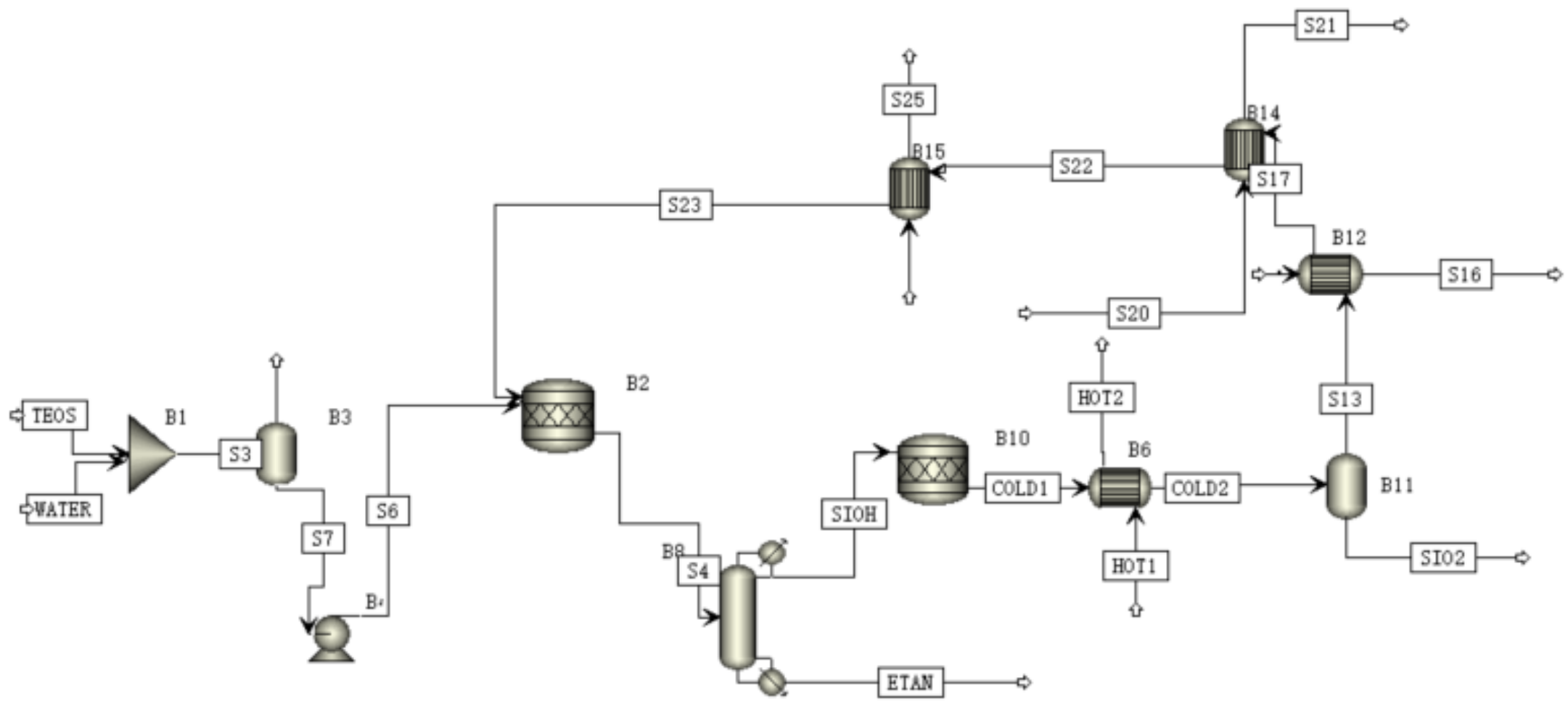

Figure S48 The detailed process simulation flowsheet of synthesizing titanosilicalites by Aspen Plus V11.

Tetraethyl orthosilicate (TEOS), tetrabutyl titanate and tetrapropylammonium hydroxide are introduced through pipelines and mixed with water. Initially, these raw materials are stored in retention tanks before being added to the reactor. In the reactor, TEOS, tetrabutyl titanate and tetrapropylammonium hydroxide react with water. After the reaction, the products are transferred via pipelines to new tanks for temporary storage. Subsequently, these products enter a distillation column, where ethanol is separated from silicon hydroxide at the column bottom. The purified products from TEOS hydrolysis are collected at the column top. The product is then pumped to an oil or air heating unit for crystallization. The heated material enters a filtration unit to remove unwanted byproducts or unreacted raw materials. A portion of the product is recycled back to the retention tank and reintroduced into the reactor, improving raw material utilization. Finally, the dried product is sent to a calcination unit, where high-temperature processes induce specific changes, completing the synthesis of the desired final material.

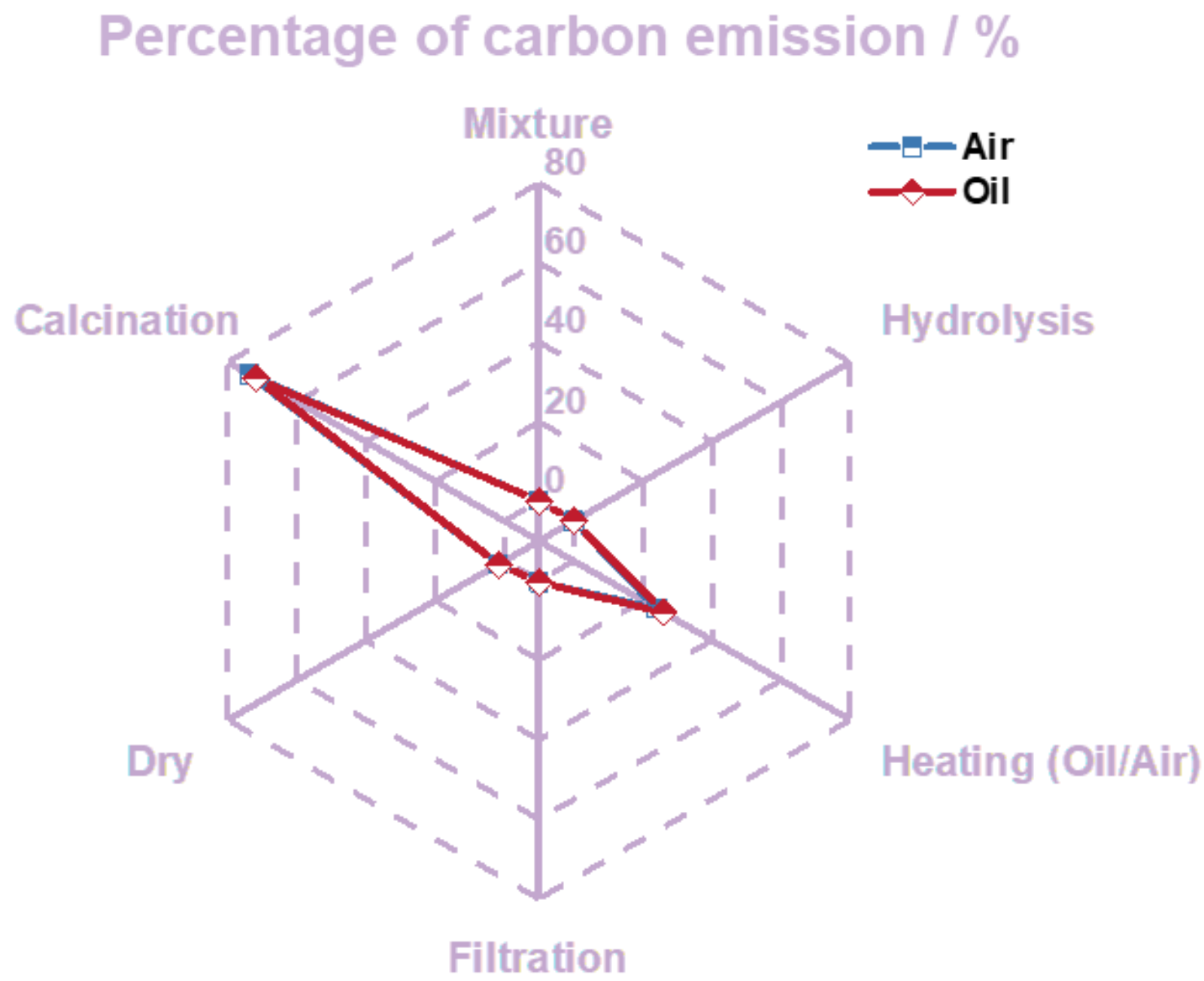

Figure S49 Comparison of carbon emission percentages for different steps in two different routes.

Mixing and hydrolysis exhibit minimal carbon emissions, approximately 0.05% each, followed by drying, which accounts for around 1.7%. The processes with the highest carbon emissions are the heating and calcination. In Route 1, calcination contributes to 71.83% of the carbon emissions, while in Route 2, it comprises 73.63%.

Regarding carbon emissions, both routes have similar emission ratios for mixing, hydrolysis, and drying, but calcination contributes the most (71.83% for Route 1, 73.63% for Route 2). The elevated proportion of carbon emissions in the calcination process is primarily attributed to the necessity of high-temperature heating, which requires substantial heat from the heat source and consequently results in increased carbon emissions. In addition, Route 1 produces significantly lower carbon emissions than Route 2, 868 kg versus 1874 kg per ton of titanosilicalite, owing to substantially accelerated TS-1 crystallization.
